# *Anthoceros* genomes illuminate the origin of land plants and the unique biology of hornworts

**DOI:** 10.1038/s41477-020-0618-2

**Published:** 2020-03-13

**Authors:** Fay-Wei Li, Tomoaki Nishiyama, Manuel Waller, Eftychios Frangedakis, Jean Keller, Zheng Li, Noe Fernandez-Pozo, Michael S. Barker, Tom Bennett, Miguel A. Blázquez, Shifeng Cheng, Andrew C. Cuming, Jan de Vries, Sophie de Vries, Pierre-Marc Delaux, Issa S. Diop, C. Jill Harrison, Duncan Hauser, Jorge Hernández-García, Alexander Kirbis, John C. Meeks, Isabel Monte, Sumanth K. Mutte, Anna Neubauer, Dietmar Quandt, Tanner Robison, Masaki Shimamura, Stefan A. Rensing, Juan Carlos Villarreal, Dolf Weijers, Susann Wicke, Gane K.-S. Wong, Keiko Sakakibara, Péter Szövényi

**Affiliations:** 1 Boyce Thompson Institute, Ithaca, NY USA; 2 Plant Biology Section, Cornell University, Ithaca, NY USA; 3 Advanced Science Research Center, Kanazawa University, Ishikawa, Japan; 4 Department of Systematic and Evolutionary Botany, University of Zurich, Zurich, Switzerland; 5 Department of Plant Sciences, University of Cambridge, Cambridge, UK; 6 LRSV, Université de Toulouse, CNRS, UPS Castanet-Tolosan, Toulouse, France; 7 Department of Ecology and Evolutionary Biology, University of Arizona, Tucson, AZ USA; 8 Faculty of Biology, Philipps University of Marburg, Marburg, Germany; 9 Centre for Plant Sciences, Faculty of Biological Sciences, University of Leeds, Leeds, UK; 10 Instituto de Biología Molecular y Celular de Plantas, CSIC-Universidad Politécnica de Valencia, Valencia, Spain; 11 Shenzhen Branch, Guangdong Laboratory for Lingnan Modern Agriculture, Genome Analysis Laboratory of the Ministry of Agriculture, Agricultural Genomics Institute at Shenzhen, Chinese Academy of Agricultural Sciences, Shenzhen, China; 12 Institute for Microbiology and Genetics, Department of Applied Bioinformatics, Georg-August University Göttingen, Göttingen, Germany; 13 Institute of Population Genetics, Heinrich Heine University Düsseldorf, Düsseldorf, Germany; 14 School of Biological Sciences, University of Bristol, Bristol, UK; 15 Department of Microbiology and Molecular Genetics, University of California, Davis, CA USA; 16 Department of Plant and Microbial Biology, University of Zurich, Zurich, Switzerland; 17 Laboratory of Biochemistry, Wageningen University & Research, Wageningen, the Netherlands; 18 Nees Institute for Biodiversity of Plants, University of Bonn, Bonn, Germany; 19 Graduate School of Integrated Sciences for Life, Hiroshima University, Hiroshima, Japan; 20 BIOSS Centre for Biological Signalling Studies, University of Freiburg, Freiburg, Germany; 21 LOEWE Center for Synthetic Microbiology (SYNMIKRO), University of Marburg, Marburg, Germany; 22 Department of Biology, Laval University, Quebec City, Quebec Canada; 23 Smithsonian Tropical Research Institute, Balboa, Panamá; 24 Institute for Evolution and Biodiversity, University of Muenster, Münster, Germany; 25 Department of Biological Sciences, Department of Medicine, University of Alberta, Edmonton, Alberta Canada; 26 BGI-Shenzhen, Shenzhen, China; 27 Department of Life Science, Rikkyo University, Tokyo, Japan; 28 Zurich-Basel Plant Science Center, Zurich, Switzerland

**Keywords:** Genetics, Plant sciences, Genomics, Evolution

## Abstract

Hornworts comprise a bryophyte lineage that diverged from other extant land plants >400 million years ago and bears unique biological features, including a distinct sporophyte architecture, cyanobacterial symbiosis and a pyrenoid-based carbon-concentrating mechanism (CCM). Here, we provide three high-quality genomes of *Anthoceros* hornworts. Phylogenomic analyses place hornworts as a sister clade to liverworts plus mosses with high support. The *Anthoceros* genomes lack repeat-dense centromeres as well as whole-genome duplication, and contain a limited transcription factor repertoire. Several genes involved in angiosperm meristem and stomatal function are conserved in *Anthoceros* and upregulated during sporophyte development, suggesting possible homologies at the genetic level. We identified candidate genes involved in cyanobacterial symbiosis and found that *LCIB*, a *Chlamydomonas* CCM gene, is present in hornworts but absent in other plant lineages, implying a possible conserved role in CCM function. We anticipate that these hornwort genomes will serve as essential references for future hornwort research and comparative studies across land plants.

## Abstract (web summary)

Analyses of three high-quality genomes of *Anthoceros* hornworts place hornworts as a sister clade to the lineage including liverworts and mosses, and provide insights into the unique biological features of hornworts.

## Main

Land plants evolved from a charophycean algal ancestor 470–515 million years ago^1^ and contributed to the greening of the terrestrial environment. The extant land plants consist of vascular plants and three bryophyte lineages—mosses, liverworts and hornworts. While the phylogeny of land plants has been debated, recent evidence indicates that bryophytes are monophyletic with hornworts a sister clade to Setaphyta (liverworts and mosses)^2–6^.

The evolution of land plants is underlined by the rise of morphological, molecular and physiological innovations. Tracing the evolutionary origins of these key innovations is prone to errors due to uncertainty in reconstructing the most recent common ancestor (MRCA) of land plants. More than 400 million years of independent evolution of the three bryophyte lineages have provided ample time for evolutionary changes to happen and the availability of model systems for only two bryophyte lineages—mosses (*Physcomitrella patens*)^7^ and liverworts (*Marchantia polymorpha*)^8^—makes inferences even more difficult. Hornworts, as the earliest diverging lineage in bryophytes, are crucial to infer character evolution and reveal the nature of the MRCA of bryophytes and that of land plants.

Hornworts uniquely possess a combination of traits that connect them with both green algae and other land plant lineages^9^. For instance, most hornworts have a single chloroplast per cell with a pyrenoid capable of carrying out a carbon-concentrating mechanism (CCM)^10^. Such pyrenoid-based CCMs cannot be found in any other land plants but frequently occur in algae^11^. Conversely, hornwort sporophytes are long-lived and moderately independent of gametophytes, which have been assumed to be features linking them to vascular plants^12^. Furthermore, hornwort sporophytes bear stomata that may be homologous with those of vascular plants^13^.

In addition to having characteristics exclusively shared with algae or with other land plants, hornworts also have a wide range of distinctive biological features. For example, the presence of a basal sporophytic meristem and asynchronous meiosis are unique to hornworts^14^. Moreover, hornworts are among the very few plants that have a symbiotic relationship with nitrogen-fixing cyanobacteria^15^ and one particular hornwort species, *Anthoceros punctatus*, has been used as a model system to study plant–cyanobacteria interactions^16^.

Detailed genomic information on hornworts is essential not only to understand the evolutionary assembly of land plant-specific traits, but also to substantiate the full potential of hornworts as a model for studying the genetic basis of cyanobacterial symbiosis and pyrenoid-based CCMs. Here, we provide three high-quality genome assemblies and their annotations for the genus *Anthoceros*. We use these data to refine our inferences on the nature of the land plant MRCA and to gain new insights into hornwort biology.

## Genome assembly and annotation

**Fig. 1:**
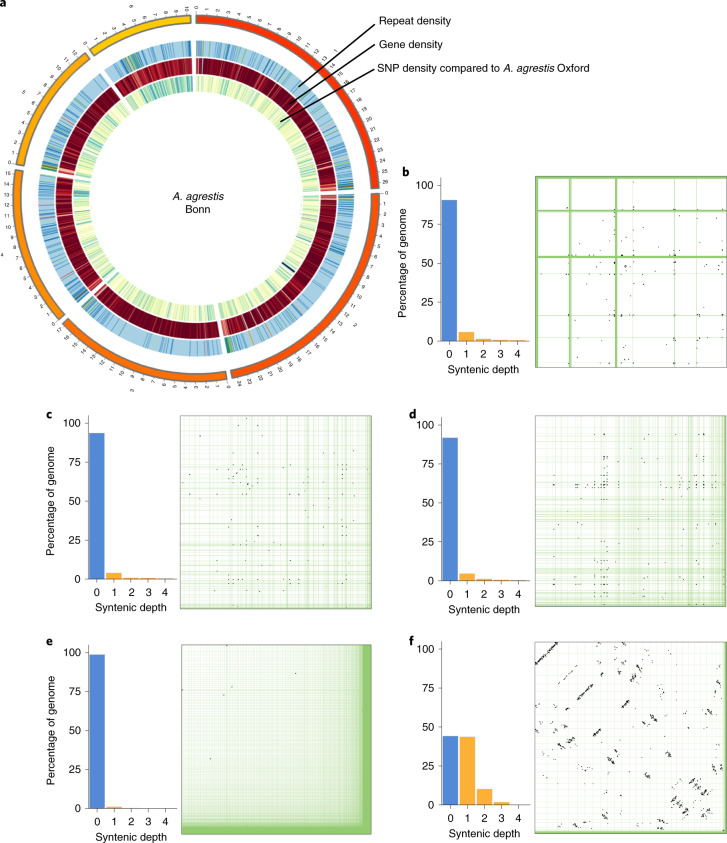
Genome organizations in the *Anthoceros* genomes. **a**, Circos plot of *A. agrestis* Bonn showing the densities of repeats, genes and single nucleotide polymorphisms (SNPs) with the *A. agrestis* Oxford genomes. **b**–**e**, *Anthoceros* genomes lack whole genome duplication. No self-synteny can be found in the three *Anthoceros* genomes (*A. agrestis* Bonn (**b**), *A. agrestis* Oxford (**c**) and *A. punctatus* (**d**)) nor in *M. polymorpha* (**e**). **f**, *P. patens*, on the other hand, shows a clear 1:1 and some 1:2 syntenic relationship, suggesting paleopolyploidy. In **b**–**f**, the bar graphs show the proportion of the genome at different self syntenic levels, with the dot-plots on the right.

We assembled three hornwort genomes from *Anthoceros agrestis* (Bonn and Oxford strains) and *A. punctatus*. For *A. agrestis* Bonn, a combination of short- and long-read data with Chicago and Hi-C libraries resulted in a chromosomal-scale assembly with the six largest scaffolds containing 95% of the assembled genome (*A. agrestis* has six chromosome pairs; Fig. 1 and Supplementary Fig. 1). For *A. agrestis* Oxford strain and *A. punctatus*, we used Oxford Nanopore sequencing to obtain high-quality assemblies composed of roughly 200 contigs with N50 over 1.7 megabase pairs (Mb) (Table 1). The three genomes are highly collinear with a greater collinearity found between the two *A. agrestis* strains (Supplementary Fig. 2 and Supplementary Table 1). The collinearity, BUSCO (Benchmarking Universal Single-Copy Orthologs) (Supplementary Fig. 3) and read mapping statistics (Supplementary Tables 2 and 3), show that the three genomes are of high quality and accuracy.

**Table 1:** Assembly statistics of the three hornwort genomes

	Estimated genome size (Mb)	Assembled genome size (Mb)	Contig/scaffold number	Contig/scaffold N50 length	Assembly approach
*A. agrestis* Bonn	122–132	116.9	1577/322	155.5 kb/17.3 Mb	Illumina + Nanopore + Hi-C
*A. agrestis* Oxford	123–135	122.9	153/–	1.8 Mb/–	Nanopore + Illumina
*A. punctatus*	128–150	132.8	202/–	1.7 Mb/–	Nanopore + Illumina

The total assembly length varied between 117 and 133 Mb, which is consistent with the size estimates based on *k*-mer analysis (Table 1) but slightly larger than those from flow cytometry^17,18^. Although these genomes are among the smallest of land plants, their repetitive and transposable element contents are considerable (36–38%). Similar to other plant genomes, the most abundant repeats are long terminal repeat elements (>20%) followed by a large number of unclassified repeats and DNA elements. The genome size variation among the three strains can be largely attributed to the differences in repeat content (Supplementary Fig. 4 and Supplementary Table 4). A combination of ab initio, evidence-based and comparative gene prediction approaches resulted in 24,700–25,800 predicted protein-coding genes (Supplementary Table 5). For *A. agrestis* we also created a pan genome combining genome assemblies and gene annotations of the two strains (Bonn and Oxford) in a non-redundant way (see Methods, Supplementary Table 5 and Supplementary notes). The three hornwort genomes show a high gene density compared to other land plants (Supplementary Table 6). All three genomes and their annotations can be accessed, browsed, searched and downloaded from ref. ^19^.

## *Anthoceros* displays unusual centromere structure

The chromosomal-level assembly of *A. agrestis* Bonn revealed some peculiarities in the hornwort genome structures. In particular, we could not locate the typical vascular plant centromeric regions, which are usually composed of highly duplicated tandem repeats of 100–1,000 base pairs (bp)^20^. In *A. agrestis* Bonn, tandem repeats with a unit size over 30 bp gave rise to only very short arrays, and these repeats do not show a clear spatial clustering (Supplementary Fig. 5). While gene density does fluctuate along the scaffolds, extensive regions with low gene density typical for centromeric regions of vascular plants were missing. Similarly, we could not identify stretches of scaffolds having an elevated repeat content (Fig. 1 and Supplementary Fig. 4), other than the putative telomeric regions. In other words, hornwort centromeres may not be characterized by a higher repeat density compared to other parts of the genome (see Supplementary Notes). Similar genome organizations were also discovered in the *P. patens* genome where genes and repeats are evenly distributed along the chromosomes^21^. While it is tempting to suggest that this genomic organization may be a shared feature of bryophyte genomes, we nevertheless cannot rule out the possibility that the bona fide centromeres were not sequenced or assembled properly despite the long-read and Hi-C data. Future work using immunolabelling is necessary to confirm this suggestion.

## Phylogenomic evidence for the monophyly of bryophytes

**Fig. 2:**
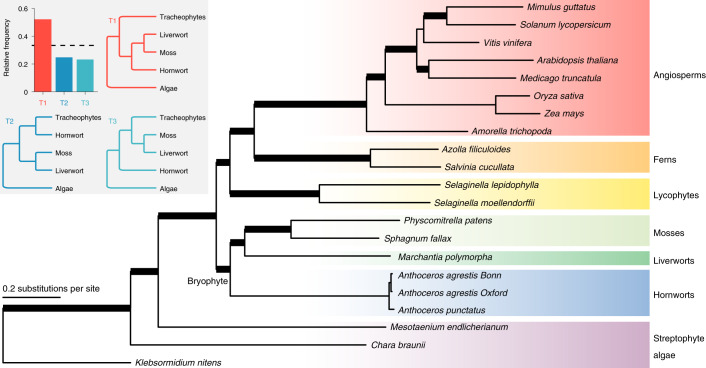
Land plant phylogeny inferred from 742 mostly single-copy genes. The monophyly of bryophytes is supported. The topology shown here is based on the maximum likelihood tree from the concatenated amino acid dataset. Thickened branches received maximal (100) bootstrap and SH-aLRT supports from both the concatenated nucleotide and amino acid datasets, as well as maximal posterior probabilities (1.0) from the Astral species-tree analysis (based on both nucleotide and amino acid gene trees). The inset shows the quartet frequencies among the 742 gene trees supporting monophyletic bryophytes (T1) versus two alternative placements of hornworts (T2 and T3). The dotted line shows the one-third threshold.

To investigate the phylogenetic position of hornworts, we compiled 742 mostly single-copy genes from 21 genomes spanning major lineages of land plants and streptophyte algae. Monophyly of bryophytes is maximally supported in all our analyses, regardless of the data types (nucleotide or amino acid), tree inference methods (concatenation- or coalescent-based) and support measures (bootstrap or SH-aLRT or local posterior probability) (Fig. 2). In addition, over 50% of the gene-tree quartets are consistent with hornworts being a sister clade to liverworts and mosses (Fig. 2). Our results add to the growing evidence^2–6^ supporting two monophyletic groups of land plants: bryophytes and tracheophytes (vascular plants).

## Limited collinearity across bryophyte and vascular plant genomes

A previous study on the moss *P. patens* genome implied that regions showing collinearity between moss and some angiosperms may represent conserved collinear blocks since the MRCA of land plants^21^. However, comparing bryophytes to vascular plants, shared ancestral gene blocks could not be identified, rather that the collinear regions with vascular plants were unique to each of the bryophyte genomes (Supplementary Fig. 6 and Supplementary Table 7). The most genomic blocks collinear with at least one other land plant were found in the moss, followed by the liverwort and hornwort genomes (Supplementary Fig. 6). Within bryophytes, no collinear segment conserved across all three lineages was found, although there were genomic regions exclusively collinear between each of two bryophyte genomes (Supplementary Fig. 6). In general, there was more collinearity between the liverwort and the moss than between the hornwort and the liverwort/moss genomes. The numbers of such collinear regions, however, were small compared to those detected across vascular plants (Supplementary Fig. 6). Altogether, these findings imply that the deep divergence of the moss, hornwort and liverwort genomes may have led to limited collinearity among bryophytes, as well as between bryophytes and vascular plants.

## Absence of large-scale genome duplication in *Anthoceros*

Whole-genome duplications (WGD) have played an important role in shaping plant evolution and possibly underlie several adaptive radiations^22^. A previous study, based on the number of synonymous substitutions per synonymous site (K_S_) divergence in transcriptomic datasets, suggested that hornworts may not have experienced any WGD event^21^, similar to *M. polymorpha*^8^ and *Selaginella moellendorffii*^23^. Our Ks plots on the annotated *Anthoceros* genes similarly show no sign of WGD (Supplementary Fig. 7). To further corroborate this, we investigated patterns of intragenomic synteny in the three hornwort genomes, as well as the published *M. polymorpha* and *P. patens* genomes for comparison. We found very little self-synteny in the hornwort genomes (Fig. 1b–d), providing strong evidence for the lack of WGD in *Anthoceros*. The high proportion of the genomes that are not syntenic is comparable to that in *M. polymorpha* (Fig. 1e). On the other hand, *P. patens* shows a clear 1:1 (and some 1:2) self-syntenic relationship (Fig. 1f), which is consistent with the earlier report and indicative of two rounds of WGD^21^.

## Small repertoire of TAPs

**Fig. 3:**
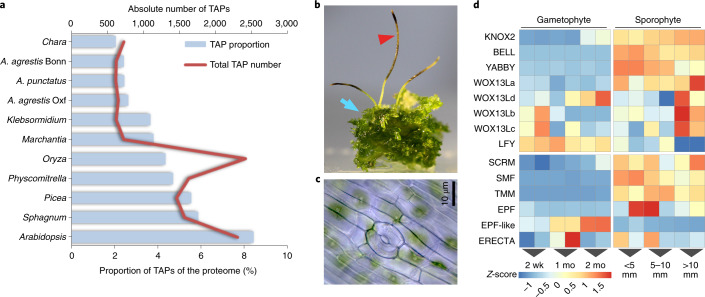
TAPs and sporophyte development. **a**, The *Anthoceros* genomes have the smallest TAP repertoire among land plants. **b**, Sporophytes (red arrowhead) and gametophytes (blue arrow) of *A. agrestis* Bonn. **c**, Stomata of *A. agrestis* Bonn. **d**, Gene expression profiles across different developmental stages in *A. agrestis* Bonn (*n* = 12 biologically independent samples; two-sided test for differential expression, false-discovery rate ≤0.05 and log_2_-fold-change ≥2). wk, week; mo, month.

We found that 2.4–2.6% of the proteomes of the three *Anthoceros* genomes were annotated as transcription-associated proteins (TAPs) (Fig. 3a and Supplementary Table 8). Compared to other land plants^24^, this is on the very low end of the spectrum both in terms of proportion and absolute number. Furthermore, about two-thirds (56) of the hornwort TAP families were smaller in size than in *M. polymorpha*. Given such a minimal TAP repertoire, hornworts can serve as an excellent baseline model to study the evolution and diversification of transcriptional networks. Despite its streamlined nature, some TAPs were only found in hornworts and vascular plants but not in the other two bryophyte genomes, with the most notable example being *YABBY* (Supplementary Table 8 and Supplementary Note). Such TAPs probably evolved in the MRCA of land plants but were lost in the mosses and liverworts. We also detected TAP families that were present in all streptophytes but lost either in the hornwort genomes (for example, SRS transcription factor, TF) or in *M. polymorpha* (for example type I MADS-box TF). Altogether, our findings suggest a dynamic TAP family turnover in the early evolution of land plants with multiple independent losses in different bryophyte lineages.

## Genes related to sporophyte development

While hornworts have a gametophyte-dominant life cycle like other bryophytes, their sporophyte generation (Fig. 3b) shows several unique features^25^. First, after fertilization, the zygote division in hornworts is longitudinal, whereas zygotes in all other land plants undergo transverse division. Second, the hornwort sporophyte maintains a basal sporophytic meristem producing cells that continuously differentiate into mature tissues towards the tip. A common origin of indeterminate sporophyte development in hornworts and vascular plant shoot apical meristem (SAM) has been hypothesized^25^. Lastly, hornwort sporophytes have stomata (Fig. 3c) similar to mosses and vascular plants, and the basic regulation may be shared across all stomatous lineages of land plants^26^. Nevertheless, firm evidence supporting the homology of meristems as well as stomata is scarce. Here, we found that multiple genes critical for flowering plant SAM and stomata function have homologues in the hornwort genomes and are preferentially expressed in the sporophyte phase.

Class 1 *Knotted1*-like homeobox (KNOX1) genes regulate sporophytic meristem activity in both *P. patens* and vascular plants^27^, while Class 2 *Knotted1*-like homeobox (KNOX2) genes maintain sporophyte cell fate in *P. patens*^28^. Interestingly, the KNOX1 orthologue is lost in the *Anthoceros* genomes and only KNOX2 genes were found (Supplementary Fig. 8 and Supplementary Tables 8 and 9). The KNOX2 orthologues showed strong sporophyte-specific expression (Fig. 3d), which implies that the involvement of KNOX2 in maintaining sporophytic cell fate may be conserved in all land plants. Heterodimerization of KNOX1/KNOX2 and BELL-LIKE HOMEOBOX proteins is a deeply conserved molecular mechanism that is required for the KNOX functions^29^. We found that in *A. agrestis* Bonn, a single *BELL* and a single *KNOX2* gene were specifically expressed in the sporophyte phase. Nevertheless, contrary to our expectations, the *BELL* gene was more strongly expressed in the early stages while the *KNOX2* gene in the later stages of sporophyte development (Fig. 3d and Supplementary Tables 8 and 9). This suggests that hornwort sporophyte identity may not be determined by KNOX2 through interaction with BELL. Nevertheless, this hypothesis needs functional verification because partially overlapping expression of the *KNOX2* and *BELL* genes does not exclude the possibility of heterodimerization.

*WUSCHEL-related homeobox 13* like (WOX13L) genes are involved in zygote development and stem cell formation in the moss *P. patens*^30^*. A. thaliana WOX13* promotes replum formation in the fruit^31^ and *WOX14* promotes vascular cell differentiation^32^. The *Anthoceros* genomes have four WOX13L members (Supplementary Fig. 8 and Supplementary Tables 8 and 9) and *WOX13La* is specifically expressed in sporophytes while *WOX13Lbcd* have expression at both gametophyte and sporophyte generations (Fig. 3d) and may have diverse roles in stem cell maintenance and sporophyte development. The *Anthoceros* genomes also have a single *FLORICAULA*/*LEAFY* (*FLO*/*LFY*) gene (Supplementary Fig. 8 and Supplementary Tables 8 and 9), which in *P. patens* and *A. thaliana* controls zygote development and SAM maintenance, respectively^33^. In hornworts, *LFY* is predominantly expressed in the gametophyte stages (Fig. 3d) while in *P. patens* it is expressed both in the gametophyte and the sporophyte. It is possible that such differences may contribute to the unique developmental pattern of hornwort sporophytes.

Stomatal development in *A. thaliana* and *P. patens* is regulated by a conserved genetic toolbox, including the basic helix–loop–helix (bHLH) transcription factors SMF (*SPCH*, *MUTE* and *FAMA*), ICE/SCREAMs (*SCRM*s), *EPIDERMAL PATTERNING FACTOR* (*EPF*), *ERECTA* and *TOO MANY MOUTHS* (*TMM*) genes^34,35^. *FAMA* in particular is involved in the final guard cell differentiation step and serves as the key switch. Orthologues of *SMF*, *TMM* and *EPF* were absent in *M. polymorpha*, consistent with the fact that liverworts do not have stomata^8^. We found orthologues of *FAMA (SMF)*, *SCRM*, *ERECTA*, *EPF* and *TMM* in the *Anthoceros* genomes (in line with a previous study based on our earlier genome draft^26^; Supplementary Table 10 and Supplementary Fig. 9). *SMF*, *SCRM*, *TMM* and *EPF* showed sporophyte-specific expression patterns (Fig. 3d), suggesting that they may have similar roles in stomatal patterning in hornworts. While *ERECTA* was also expressed during early sporophyte development, its expression fluctuated between replicates and results were inconclusive. *EPF* expression showed similar inconsistency among replicates but did not influence our conclusion about its sporophyte-specific expression. In addition to *EPF*, an *EPF*-like gene in the EPFL4-6 clade, was found in hornworts (Supplementary Fig. 9), and is specifically expressed in gametophytes with a higher expression toward maturity and thus perhaps involved in a different cell–cell signalling other than stomatal regulation. *EPF4* and *EPF6* in *A. thaliana* are involved in coordination of the central and peripheral zone in SAM^36^. Taken together, our data are consistent with a single origin of stomatal differentiation mechanism among all stomatous land plants, though positional determination may have evolved differently (Supplementary Notes).

## Genes related to phytohormone synthesis and signalling

The *Anthoceros* genomes contain the genetic chassis for the biosynthesis and signalling of abscisic acid, auxin, cytokinin, ethylene and jasmonate (see Supplementary Notes, Supplementary Figs. 10–12 and Supplementary Table 10), reaffirming the origins of these pathways in the MRCA of land plants^7,8,37^. Similar to *M. polymorpha* and *P. patens*, salicylic acid signalling components, but not the receptor-related genes, are found in hornworts. While *DELLA* is present, orthologues of gibberellin (GA) receptor GID1 and GA oxidases are missing from the *Anthoceros* genomes. This is consistent with the recent suggestion that DELLA was recruited to the GA signalling pathway later in plant evolution^38^. Hornworts also possess enzymes to synthesize strigolactones but genes involved in strigolactone signalling are absent. This supports the idea that strigolactones are an ancient non-hormonal signal for rhizospheric communication with mycorrhizal fungi^39^.

## Genetic network for arbuscular mycorrhizal symbiosis was present in the MRCA of land plants

The symbiotic relationship with arbuscular mycorrhizal fungi (AMF) is one of the key innovations underlying the successful colonization and diversification on land of plants. Evidence of AMF can be found inside plant megafossils 407 million years ago^40,41^ and in almost all extant plant lineages (hornworts, liverworts and vascular plants). Recent genetic studies have identified a suite of genes in the angiosperms that regulate the establishment and maintenance of AMF symbiosis^42^. Some of these genes are also required for legume–rhizobial interaction and are often referred to as the common symbiosis genes^43^.

**Fig. 4:**
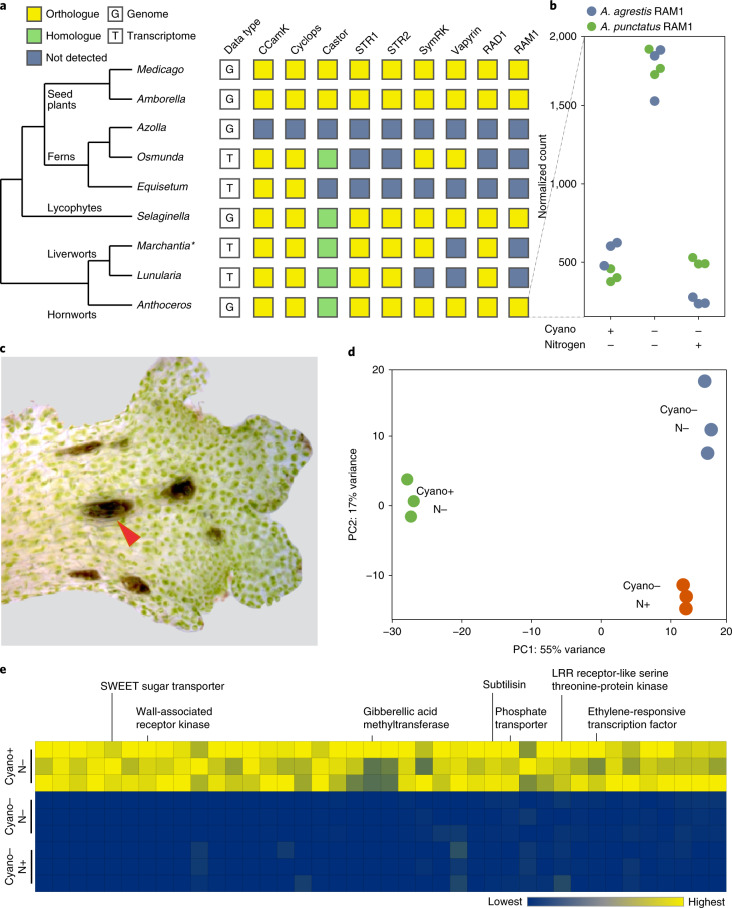
Evolution and genetics of symbiosis in hornworts. **a**, Orthologues of AMF symbiosis pathway genes can be found in hornworts, indicating their presence in the common ancestor of land plants. The asterisk indicates that the *M. paleacea* transcriptome was searched instead of *M. polymorpha* genome because the latter secondarily lost AMF. **b**, RAM1 is upregulated during nitrogen starvation in both *A. agrestis* and *A. punctatus*. **c**, Reconstituted *Anthoceros*–cyanobacteria symbiosis. Arrowhead points to a cyanobacteria colony. **d**, Transcriptomic responses to nitrogen starvation and cyanobacterial symbiosis in *A. agrestis* (*n* = 9 biologically independent samples). PC1 and PC2 refer to the first and second axes of principal component analysis on gene expression values. **e**, A suite of genes were highly upregulated under symbiosis in both *A. agrestis* and *A. punctatus* (two-sided test for differential expression, false-discovery rate ≤0.05 and log_2_-fold-change >4).

While a few components can be traced back to as far as charophyte algae^44^, the question of when exactly did the entire AMF symbiosis genetic network originate remains open. This is partly because both the bryophytes that have published genomes to date (*P. patens* and *M. polymorpha*) are incapable of AMF symbiosis and may have secondarily lost the symbiosis genes, as exemplified in some angiosperms^45^. Here, we show that all the key angiosperm AMF symbiosis genes have orthologues in the three hornwort genomes (Fig. 4, Supplementary Table 11 and Supplementary Fig. 13). Although their roles in hornwort AMF symbiosis remain to be tested, this result provides strong evidence that the genetic infrastructure required for AMF symbiosis was already present in the MRCA of land plants. Importantly, the presence of these genes in liverworts^44^ and hornworts makes this conclusion insensitive to any uncertainty of the land plant phylogeny. We have not succeeded in reconstituting hornwort–AMF symbiosis in vitro and hence are unable to test expression of these orthologues in the context of AMF. Nevertheless, we found that in both *A. agrestis* (Oxford strain) and *A. punctatus*, one of the AMF symbiosis genes, *RAM1*, was upregulated when plants were nitrogen-starved (Fig. 4). Nitrogen limitation is a major trigger for cyanobacteria symbiosis in hornworts, which might implicate the involvement of RAM1 in symbiosis, but further genetic studies are needed.

## Genes related to cyanobacterial symbiosis

Symbiosis with nitrogen-fixing cyanobacteria is a rare trait, with limited appearances in a few plant lineages: bryophytes, *Azolla* (ferns), cycads (gymnosperms) and *Gunnera* (angiosperms)^15,46^. In bryophytes, although mosses frequently harbour epiphytic cyanobacteria^47^, only hornworts and two liverwort species host cyanobacteria endophytically within specialized slime-filled cavities^15,46^. Amongst all the plant associations with cyanobacteria, most of the research has been done on hornworts, using *A. punctatus* (sequenced here) and the cyanobacterium *Nostoc punctiforme* as the study system.

Although several cyanobacterial genes from *N. punctiforme* have been identified that are key to initiation of symbiotic association^16^, nothing is known about the hornwort genetics. Here we generated RNA-seq data to compare the gene expression of symbiont-free (either nitrogen-starved or nitrogen-fed) and symbiosis-reconstituted hornworts (Fig. 4). This experiment was conducted with both *A. punctatus* and the *A. agrestis* Oxford isolate. We identified 40 genes that, when the cyanobionts are present, are highly induced (>16-fold) in both hornwort species (Fig. 4 and Supplementary Table 12). These include a number of receptor kinases, transcription factors and transporters. Of particular interest is a SWEET sugar transporter in the *SWEET16/17* clade (Fig. 4 and Supplementary Fig. 14), which is minimally transcribed under the symbiont-free states but is among the highest expressed genes in symbiosis (>10^3^ fold-change). The upregulation of *SWEET* in symbiosis is interesting because it implies that this sugar transporter is dedicated to supplying carbon rewards to the cyanobionts. This implication is supported by the fact that only exogenous glucose, fructose or sucrose sustained dark nitrogen fixation in the *A*. *punctatus*–*N*. *punctiforme* association^48^ and by the observation that inactivation of a carbohydrate permease in *N*. *punctiforme* resulted in a defective symbiotic phenotype^49^. In parallel, SWEET is involved in mycorrhizal symbiosis as well, but a different orthologue, in the *SWEET1* clade, was recruited^50^.

Another gene of interest is subtilase. Members of this gene family have been shown to be highly upregulated in a wide variety of microbial symbioses, including rhizobial^51^, mycorrhizal^52^ and actinorhizal^53–55^ interactions. RNA interference knockdown of a subtilase (*SBTM1*) in the legume *Lotus japonicus* resulted in a decreased arbuscule formation^52^. Here, we found that in both *A. punctatus* and *A. agrestis*, a subtilase homologue was similarly induced by cyanobacteria symbiosis. Phylogenetic reconstruction showed that this hornwort subtilase is not orthologous to those involved in other plant symbioses (Supplementary Fig. 15). Taken together, our results imply that hornworts might have convergently recruited SWEET and subtilase for cyanobacterial symbiosis, although in both cases not the same orthologues were used as in other plant–microbe symbioses.

## Pyrenoid-based CCM

To enable a more efficient photosynthesis, hornworts, cyanobacteria and many eukaryotic algae have evolved biophysical CCM inside their cells (cyanobacteria) or individual chloroplasts^56^. Algal and hornwort chloroplasts use inorganic carbon transporters and carbonic anhydrases to locally concentrate CO_2_ in the pyrenoids, a specialized chloroplast compartment where RuBisCOs aggregates. Pyrenoids can thus boost photosynthetic efficiency and reduce photorespiration. Such pyrenoid-based CCM has been extensively studied in the model green alga *Chlamydomonas reinhardtii* with the hope of installing a CCM in crop plants^57^.

**Fig. 5:**
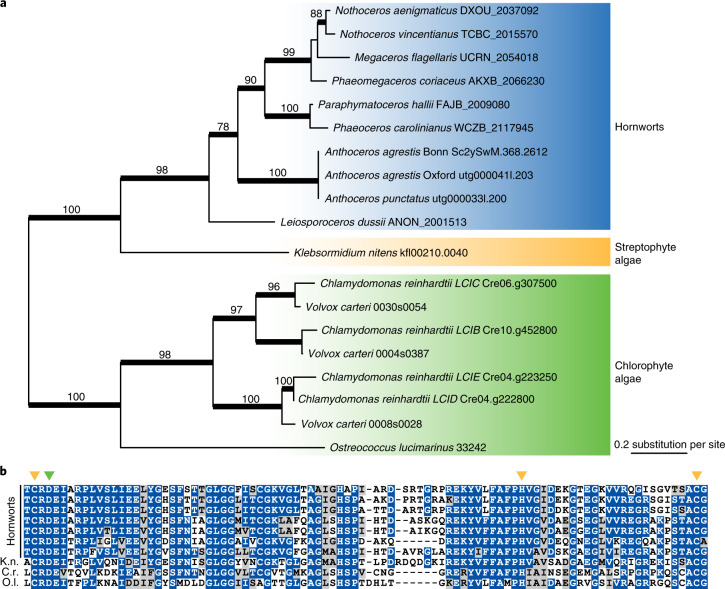
Relationship between *LCIB* and pyrenoid-based CCM. **a**, Phylogeny of *LCIB*. Numbers above branches are bootstrap support values (branches thickened when bootstrap >70). **b**, Hornwort *LCIB*s have conserved amino acid residues at the active site. Yellow and green arrowheads point to the zinc-binding and catalytic residues, respectively. K.n., *K. nitens*; C.r., *C. reinhardtii*; O.l., *Ostreococcus lucimarinus*.

Hornworts are the only land plants with a pyrenoid-based CCM. Interestingly, for the past 100 million years, pyrenoids in hornworts are inferred to have been repeatedly lost and gained^58^, suggesting that pyrenoid development and function is controlled by a few master switches. The genetics behind hornwort pyrenoids, however, has remained completely unknown. We explored whether hornwort genomes have genes that are known to be required for pyrenoid-based CCM in *C. reinhardtii*. While many of the *C. reinhardtii* CCM genes^57^ do not have clear homologues in hornworts (nor in any other land plants), we did find *LCIB* (low CO_2_ inducible B) to be present in the hornwort genomes and hornwort transcriptomes of the 1,000 plant transcriptomes project (1KP)^6^ (Fig. 5). Apart from hornworts, no *LCIB* homologue could be found in other plant genomes sequenced to date. The uniquely shared presence of LCIB in pyrenoid-bearing algae and hornworts implies that *LCIB* might have a role in the hornwort CCM. The phylogenetic tree indicates that the hornwort *LCIB*s form a sister clade to the *Klebsormidium nitens* homologue (Fig. 5) and thus is consistent with the organisms tree with many losses in various lineages. In this scenario, the MRCA of land plants had LCIB.

In *C. reinhardtii*, *LCIB* gene expression is highly induced by CO_2_ limitation and the encoded proteins localize around pyrenoids to presumably block CO_2_ leakage^59,60^. All the hornwort LCIB sequences have the conserved amino acid residues at the active sites that are shared with other algal LCIBs^61^ (Fig. 5b). However, unlike *C. reinhardtii*, we did not find *LCIB* to be differentially expressed when plants are grown at different CO_2_ levels (Supplementary Fig. 12). This, nevertheless, cannot rule out the involvement of *LCIB* in CCM because hornwort CCM was reported to be constitutively expressed and not regulated by CO_2_ level^62^. Whether *LCIB* homologues have a similar function and localization in hornworts remains to be experimentally tested.

## Discussion

The hornwort genomes presented here offer a unique window into the biology of land plant MRCA. For example, the *Anthoceros* genomes lack KNOX1, while *P. patens* and *M. polymorpha* lack *YABBY* genes. This suggests that the MRCA of land plants had both of these key developmental genes and independent gene losses occurred in different bryophyte lineages. While *LEAFY* expression is predominantly in the gametophyte stage, *YABBY*, *KNOX2*, *BELL* and some *WOX13L* genes are up-regulated in the hornwort sporophytes (Fig. 3). In addition, several stomata-related genes are present in the *Anthoceros* genomes and expressed in early sporophyte development (Fig. 3), implying a homology of stomata at the genetic level. Finally, we found that the genes required for AMF symbiosis are conserved in *Anthoceros* (Fig. 4), providing evidence that the MRCA of land plants was already equipped with the genetic network for AMF symbiosis. In-depth analysis on the evolution of the plant hormones (abscisic acid, auxin, gibberellin, jasmonate, salicylic acid and strigolactone), light signalling, peptidoglycan synthesis and chloroplast development can be found in Supplementary Notes.

The *Anthoceros* genomes shared several features with the two other published bryophyte genomes. Most notable is the absence of tandem repeats that make up the typical centromeric regions. Further studies are needed to identify the centromeric regions and understand their structure. While *P. patens* has experienced two rounds of WGD^21^, none can be found in *Anthoceros* and *M. polymorpha* (Fig. 1). This might explain the minimal representation of transcription factors in the last two genomes.

Furthermore, our functional genomic data shed light on the genetic framework that underpins features that are unique to hornworts. We identified a suite of candidate genes underlying hornwort–cyanobacteria symbiosis (Fig. 4). This includes a SWEET transporter that might be involved in nutrient transfer with the cyanobionts. A well-characterized *C. reinhardtii* CCM gene, *LCIB*, was conserved in hornworts but apparently lost in all other plant lineages (Fig. 5). Whether *LCIB* also participates in hornwort CCM awaits future functional characterization.

The recent advances of ‘seed-free genomics’ have greatly improved our understanding of streptophyte evolution^8,21,23,37,63–66^. Here, our hornwort genomes fill in yet another critical gap and are beginning to illuminate the dawn of land plants as well as the unique biology of hornworts.

## Methods

### Plant materials

Cultures of *A. agrestis* (Oxford and Bonn strains) and *A. punctatus* were all derived from a single spore and axenically propagated and maintained on either BCD^67^ or Hatcher’s^68^ media. Supplementary Table 13 shows the origin and specimen voucher for each of the three strains.

### Chromosome count

The tip of an *A. agrestis* Oxford gametophyte thallus was cut into small pieces and fixed with 4% glutaraldehyde in 0.05 M phosphate buffer (pH 7.0) for 12 h at 4 °C. After washing with the buffer for 10 min, cell walls were digested for 2 h with a solution containing 1% Driselase (Sigma-Aldrich), 1% Cellulase Onozuka RS (Yakult), 1% Pectolyase (Kikkoman), 0.5% IGEPAL CA-630 and 1% bovine serum albumin (BSA) at 30 °C. After several washes with the buffer, the samples were incubated in 0.05 M phosphate buffer containing 0.1% TritonX-100 for 12 h at 4 °C. After several washes with the buffer, the samples were transferred onto MAS coated slide glasses (Matsunami Glass) and coverslipped. The slides were then pressed with a thumb directly over the coverslip. After removal of the coverslip, the slides were air-dried for 10 min at room temperature and then extracted with methanol at −20 °C for 10 min. After the staining with the buffer containing 1 μg l 4,6-diamidino-2-phenylindole (DAPI) for 5 min, the slides were mounted with Vectashield mounting medium (Vector Laboratory Burlingame) and observed with a fluorescence microscope under ultraviolet-light excitation.

### DNA sequencing

Hornwort DNA was extracted using a CTAB-precipitation method modified from ref. ^69^. Nanopore libraries were prepared by SQK-LSK108 and sequenced on MinION R9 flow cells for 48 h. Basecalling was done by Albacore.

For *A. agrestis* Bonn, the TrueSeq DNA Nano Kit (Illumina) was used to prepare paired-end (PE) sequencing libraries which were sequenced (PE 150 bp) on HiSeq4000 at the Functional Genomic Center Zurich (FGCZ). For *A. agrestis* Oxford 251 PE reads, a PCR-Free library was prepared using a KAPA Hyper Prep Kit according to the protocol published by Broad Institute^70^). The library was mixed (5%) with other barcoded libraries and sequenced on Illumina HiSeq1500 (two lanes with Rapid mode; OnBoardClustering) at the National Institute of Basic Biology. For *A. punctatus*, Illumina genomic libraries were prepared by BGI and sequenced on HiSeq4000. Read quality and adaptor trimming was done by fastp^71^ with the default setting. For *A. agrestis* Bonn, additional Chicago and Hi-C libraries were prepared by DoveTail Genomics. A total of two Chicago libraries and one Hi-C library were prepared with a physical coverage of 300× and 200×.

To calculate the read mapping rates, trimmed reads were mapped to the final assemblies using bwa mem -M^72^ and sorted with samtools^73^. The mean insert size and #READ_PAIRS were calculated using picard CollectInsertSizeMetrics. Unmapped reads were counted with samtools view -c -f 4 (ref. ^73^) and divided with the total number of reads to calculate percentage mapped. High-quality mapped reads were counted with -q 20. Reads mapped to chloroplast and mitochondrial genomes were counted with samtools. The bam files were assessed with qualimap v.2.2.1 (ref. ^74^) bamqc and observed error rates (total, mismatch, insertions and deletions), as well as the genome coverage were recovered.

### Genome assembly

Genome sizes for the three *Anthoceros* were estimated based on *k*-mer distribution by Jellyfish^75^ in conjunction with GenomeScope^76^. Draft assembly for *A. agrestis* Bonn strain was first generated using a hybrid approach including Oxford nanopore (~60×) and Illumina paired-end reads (~150×) using MaSuRCA v.3.2.8 (ref. ^77^). After assembly, base call quality was improved by two rounds of Pilon polishing^78^. We mapped Chicago and Hi-C reads back to the draft assembly and used DoveTail’s HiRise assembler v.2.1.2 (ref. ^79^) for scaffolding. Contigs of the draft assembly were first scaffolded using the Chicago library to correct smaller scale errors and improve contiguity. Finally, the output assembly was further scaffolded using the Hi-C libraries and DoveTail’s HiRise assembler v.2.1.2 (ref. ^79^) to derive the final assembly.

Genome assemblies of *A. agrestis* Oxford strain and *A. punctatus* were generated with the minimap2-miniasm assembler^80^ using only the nanopore reads. We then used four iterations of minimap2-racon^81^ to derive the consensus sequence, followed by six rounds of Pilon polishing^78^.

### Contamination removal

While our cultures were grown in a putative axenic condition, low level of contamination cannot be completely ruled out. We therefore used blobtools^82^ to identify scaffolds/contigs primarily consisting of contaminant sequences. The Hi-C library theoretically should sort DNA sequences originating from different organisms because cross-linking occurred within the nuclei. Therefore, we hypothesized that dropping scaffolds mainly with non-streptophyte affiliation will effectively remove contaminants from our assembly. For *A. agrestis* Bonn, we used both the full uniprot and the National Center for Biotechnology Information (NCBI) nucleotide database and blobtools to assign the taxonomic affiliation to each scaffold with an *e*-value of 10^−4^. We found that some of the small scaffolds were classified as of non-streptophyte origin with high confidence; these scaffolds were then removed from the assembly. For the *A. agrestis* Oxford and *A. punctatus* genomes, assemblies were contamination-filtered in a similar way. The detailed summary can be found in Supplementary Table 14.

### RNA-seq dataset and analysis on developmental stages

To study the expression pattern of transcription factor genes across developmental stages, we generated RNA-seq libraries for the following stages of the *A. agrestis* Bonn strain in two biological replicates: (1) spores after 2 weeks of germination, (2) 4-week-old gametophytes, (3) 2-month-old gametophytes, (4) sporophytes shorter than 5 mm, (5) sporophytes of 5–10 mm, (6) sporophytes longer than 1 cm with brown or black tips. Plants were grown on agar plates containing BCD medium^67^ at 22 °C. RNA was extracted with the Spectrum Total RNA Plant Kit (Sigma-Aldrich) and stranded RNA-seq libraries were prepared using the TrueSeq Stranded mRNA Library Prep Kit (Illumina). Libraries were sequenced at the FGCZ on a HiSeq4000 machine. We used trimmomatic^83^ to quality filter and trim the raw reads. Gene expression was estimated using Salmon^84^ and differential expression done by DESeq2 (log_2_-fold ≥2, false-discovery rate ≤0.05 and normalized reads counts)^85^.

We also generated separate thallus RNA-seq data for the Oxford strain (for annotation purpose). The plants were cultured on solid BCD plates and total RNA was extracted using RNeasy Plant Mini Kit (QIAGEN). The library was prepared using the TrueSeq stranded mRNA Library Prep Kit (Illumina) and sequenced on Hiseq1500.

### RNA-seq dataset and analysis on cyanobacterial symbiosis

Liquid cultures of *A. agrestis* Oxford and *A. punctatus* were used in this experiment. To establish liquid cultures, plants were transferred from solid BCD plates to flasks with 100 ml of BCD media solution and placed on an orbital shaker at 130 r.p.m. for 2 weeks. For the cyano–/N+ and cyano–/N– conditions, plants were transferred to fresh new BCD solution with and without KNO_3_, respectively and grown for 10 d before harvest. To reconstitute cyanobacterial symbiosis (with *N. punctiforme* ATCC 29133), we followed the method of Enderlin and Meeks^86^ but using BCD as the growth medium. Three biological replicates were done for each condition. RNA was extracted by the Spectrum Total RNA Plant Kit (Sigma-Aldrich). The Illumina libraries were prepared by BGI and sequenced on HiSeq4000. Sequencing reads were mapped to the respective genomes by HiSat2 (ref. ^87^) and transcript abundance quantified by Stringtie^88^. We used DESeq2 (ref. ^85^) to carry out differential gene expression analysis, with false-discovery rate set to 0.005 and log_2_-fold-change threshold set to 1. To identify genes that are differentially expressed in both *A. agrestis* Oxford and *A. punctatus*, we used the Orthofinder gene family classification results (see below) coupled with phylogenetic analysis if needed.

### RNA-seq dataset and analysis on CO_2_ response

For the CO_2_ experiment, we grew hornworts in magenta boxes with vented lids to allow air circulation while maintaining sterility. *A. agrestis* Oxford strain was used in this experiment and kept on solid BCD medium. We subjected the plant cultures to one of the three CO_2_ environments at 150 ppm (low), 400 ppm (ambient) and 800 ppm (high) in a CO_2_-controlled growth chamber for 10 d (12 h/12 h day/night cycle). Three biological replicates were done for each treatment. RNA was extracted by the Spectrum Total RNA Plant Kit (Sigma-Aldrich). The Illumina libraries were prepared by BGI and sequenced on HiSeq4000. One of the low CO_2_ samples failed to produce high-quality library, and as a result the low CO_2_ condition has only two replicates. RNA-seq data analysis was done following the same procedure as described above. We used BiNGO^89^ for gene ontology enrichment analysis and REVIGO^90^ to summarize and visualize the results.

### Repeat annotation

For repeat annotation, we first built custom repeat libraries for each genome using RepeatModeler^91^ and LTR_retriever^92^. The libraries were filtered to remove protein-coding genes by blasting against the UniProt plant database. We then used RepeatMasker^93^ to annotate and mask the repetitive regions for each genome.

### RNA-seq, transcript and protein evidence

We pooled *A. agrestis* Bonn, Oxford and *A. punctatus* RNA-seq reads together and mapped them onto each of the genome assemblies using HiSat2 (ref. ^87^). We used all RNA-seq evidence available owing to the low nucleotide divergence among the three genomes. Transcriptomes were assembled for each species/strain separately. We used Portcullis^94^ to filter out bad splice junctions and Stringtie^88^ to assemble the transcripts. We additionally used Trinity^95^ to generate both de novo and genome-guided transcriptome assemblies. We combined Trinity transcripts using the Program to Assemble Spliced Alignments (PASA) pipeline^96^ and derived high-quality transcripts with Mikado^97^. To obtain protein homology information, we retrieved the 19 proteomes (only primary transcripts; Supplementary Table 15) and aligned them to the genome assemblies using exonerate^98^. We kept only hits with at least 60% coverage and a similarity above 60%.

### Gene prediction

We used RNA-seq, transcript and protein evidence to train Augustus (ref. ^99^) within Braker2 (ref. ^100^). Because the resulting gene models were heavily dependent on the training data, we decided to generate multiple gene predictions and build consensus gene models using EVidenceModeler (EVM)^101^. We used both individual approaches (prediction of genes for each genome separately, see (1)–(5) below) and comparative (simultaneous prediction of gene models for the genomes, see (5) below) approaches to increase the accuracy and compatibility of gene annotations. Comparative genome annotation approaches use whole-genome alignment and external evidence (RNA-seq, protein and expressed sequence tag) to simultaneously predict genes in multiple genomes and are able to correct errors may arise during individual-based predictions. The following gene prediction approaches were used. (1) We trained Augustus with only the RNA-seq evidence and predicted gene models by taking into account RNA-seq, protein, Mikado and PASA assembled transcripts. (2) We used the previously trained (in (1)) species model but with a modified weighting file (extrinsic.cfg) to give more weight to the protein evidence. (3) We trained Augustus using both protein and RNA-seq evidence within Braker2 (EPT mode of Braker2). (4) We used the RNA-seq evidence to automatically train genemark and obtain gene predictions. (5) Finally, we ran Augustus in the comparative mode with RNA-seq, transcript and protein evidence and genome alignments inferred by mugsy^102^. Generating this series of genome predictions was necessary as our preliminary analyses suggested that none of the predictions was superior but rather complementary. The proteomes used can be found in Supplementary Table 15.

### Generating consensus gene models

We used EVM to derive consensus gene models best supported by the various evidence. We used all the previously generated gene predictions (gff files) and selected the best consensus gene models using protein (exonerate-mapped proteomes of species and the uniprot_sport plant dataset) and transcript evidence (Mikado and PASA assembled transcripts). We gave equal weights to each ab initio predictions, transcript evidence (weight 1), but increased the weight for Mikado loci (2) and PASA assembled transcripts (10). After deriving the consensus gene models, we used PASA and the PASA assembled transcripts to correct erroneous gene models, add UTRs (untranslated regions), and predict alternative splice variants in two rounds. Finally, we extensively manually curated these three annotations (revised and corrected various gene models) and used them for all further downstream analyses.

Our annotation pipeline resulted about 1,000 more predicted gene models for the *A. agrestis* Bonn compared to the Oxford strain. This suggested that despite high collinearity, gene content of the two strains may differ. To aid future comparative analyses we created an *A. agrestis* pan genome containing a non-redundant set of genomic sequences and annotations of the two strains. Furthermore, we carefully analysed the predicted gene set of the two strains to show that gene number difference is not due to annotation issues. Methods and results of the pan genome construction as well as gene set comparison can be found in the Supplementary notes.

### Genome completeness assessment

We used BUSCO v.3 (ref. ^103^) with the Viridiplantae set to assess the completeness of our genomes and annotations. We did not use the Embryophya set because it was constructed based almost exclusively on flowering plant genomes (29 out of 30)^103^, which does not offer an appropriate benchmark for non-flowering plant genomes. Supplementary Fig. 3 shows that our genomes have similar (if not better) BUSCO scores compared to many published non-flowering plant genomes. It should be noted that while *Physcomitrella*, *Sphagnum* and *Marchantia* all have much higher BUSCO scores, this is probably reflecting the fact that these genomes were used to compile the Viridiplantae set.

### Reconstructing the land plant phylogeny

We used Orthofinder2 (ref. ^104^) to identify mostly single-copy genes, with 21 genomes (Fig. 2) included in the run to represent angiosperms, ferns, lycophytes, mosses, liverworts, hornworts and the grade of streptophyte algae. A total of 742 mostly single-copy orthogroups were identified. Protein alignments for individual orthogroup were done by MAFFT v.7.427 (ref. ^105^) and back translated to nucleotides by TranslatorX^106^. The alignments were processed to remove sites with over 50% gaps or Ns and remove sequences shorter than 50% of the alignment length. When a species had more than one copy in an orthogroup, none from that species was included. To infer gene trees, we used both the amino acid and nucleotide matrices, and employed the maximum likelihood method implemented in IQ-Tree v.1.6.12 (ref. ^107^). The best-fitting substitution models were selected by ModelFinder^108^. To reduce saturation in nucleotide substitution at this large time scale, the third codon position was excluded.

To infer the species tree, we used both concatenation and multispecies coalescent approach. The concatenated dataset included all the 742 loci and was analysed using IQ-Tree with ModelFinder model selection. To assess branch supports, we carried out ultrafast bootstrap^109^ and SH-aLRT^110^ analyses (both with 1,000 replicates). For the multispecies coalescent approach, we used ASTRAL-III (ref. ^111^) to summarize all the 742 gene trees and measured branch supports as local posterior probabilities^112^. Gene-tree/species-tree discordance in terms of quartet frequencies was calculated by DiscoVista^113^.

### Collinearity of the three hornwort genomes and collinearity across Viridiplantae

We used the D-GENIES dot-plot tool^114^ with the default options to visually assess collinearity of the three genome assemblies. We also aligned the genomic sequences using the nucmer module of mummer^115^ and assessed their differences using Assemblytics^116^.

To study the collinearity across all plants, we first created orthogroups with proteomes of 19 species using Orthofinder2 (ref. ^104^). The dataset included representatives from each major groups of land plants (Supplementary Table 15), and species experienced different numbers of large-scale duplication events^117^. Gff files and proteomes were retrieved from Phytozome v.12 (ref. ^118^). We used I-ADHore3 (ref. ^119^) to detect highly degenerate collinear blocks among bryophytes and vascular plants requiring a minimum of three, four and five anchor points within each collinear region (gap_size=30, cluster_gap=35, q_value=0.75, prob_cutoff=0.01, anchor_points=5, alignment_method=gg2, level_2_only=false).

### Identification of tandem repeats and centromeres

We run Tandem Repeats Finder^120^ to identify tandem repeats with a minimum alignment score of 50 and a maximum period size of 2,000 bp. We then plotted repeat unit size against tandem array size to look for bimodal distribution. To localize centromeric regions in the *A. agrestis* Bonn genome, we generated dot-plots between a short-read-only assembly and the final chromosome-scale assembly. Because centromeric repeats are difficult to assemble using short-reads we expected that they will be missing from the Illumina assembly but will be present in the chromosomal-scale assembly. We also generated a self dot-plot of the *A. agrestis* Bonn genome to search for regions that are highly similar across scaffolds and are repetitive. Finally, we used the output of Tandem Repeats Finder^120^ to search for tandem arrays with a period length of minimum 10 bp and with a minimum tandem array length of 30 repeat units. We plotted the location of these tandem arrays along the chromosomes to visually assessed their distribution.

### Screening for whole-genome duplication

We used a combination of synonymous divergence (Ks) and synteny analyses to look for evidence of whole-genome duplication in the *Anthoceros* genomes. For each genome, we used the DupPipe pipeline to construct gene families and estimate the age of gene duplications^121^. We translated DNA sequences and identified reading frames by comparing the Genewise^122^ alignment to the best-hit protein from a collection of proteins from 25 plant genomes from Phytozome^118^. For each analysis, we used protein-guided DNA alignments to align our nucleic acid sequences while maintaining reading frame. We then used single-linkage clustering to construct gene families and estimate K_S_ divergence using phylogenetic analysis by maximum likelihood (PAML)^123^ with the F3X4 model for each node in the gene family phylogenies. Because the *Anthoceros* genomes contain large numbers of pentatricopeptide repeat genes (PPR), we also repeated the analysis with all the PPR genes removed. PPR genes were identified based on the Orthofinder results (see later).

For synteny analysis, we used MCscan’s ‘jcvi.compara.catalog ortholog’^124^ function to search for and visualize intragenomic syntenic regions. The default *C*-score of 0.7 is used to filter low-quality hits. To calculate syntenic depths, the ‘jcvi.compara.synteny depth’ function was used. For comparison, we also carried out the same analysis for *P. patens* v.3.3 and *M. polymorpha* v.3.0 genomes; the former is known to have two rounds of WGD while the latter has none^8,21^.

### Transcription factor annotation

TAPs were annotated using TAPscan, according to Wilhelmsson et al.^24^ and compared with selected other organisms using the major protein of each gene model (‘.1’ splice variant). TF annotations were further manually checked and adjusted for annotation errors or missing annotations.

### Gene family classification and curation

We used Orthofinder2 (ref. ^104^) to classify gene families of 25 plant and algal complete genomes, including the three hornworts reported here (Supplementary Table 16) into orthogroups. Orthofinder was run using the default setting, except that the ‘msa’ option was used. A total of 31,001 orthogroups were circumscribed. The detailed gene count and classification results can be found in Supplementary Table 16. While Orthofinder2 (ref. ^104^) provides an automatic circumscription of gene families, they rarely correspond to their expert-based circumscriptions and can contain a substantial number of misclassified gene models due to the inherent limitations of the automatic classification algorithms. Therefore, all Orthofinder2 generated gene families selected for detailed evolutionary analyses were manually curated by their experts. In particular, members of all extensively investigated gene families were checked for the presence of their domain structure either using InterPro^125^, Pfam^126^ or CCD^127^ to remove false positives and/or correct improperly predicted gene models. Furthermore, to ensure that a gene is truly absent (and not just unannotated), we carried out additional searches on the genome assemblies. For each extensively analysed gene family, we directly searched the raw genomic sequence using bryophyte or vascular plant homologues as query sequences to find additional gene models that might have been missed by our gene prediction pipeline. These searches were done using tBLASTn^128^ and, in case no hit was found, were repeated with the hmmsearch module of HMMER^129^ using the corresponding hmmer profiles from Pfam. Indeed, the manual curation helped us to add, revise and correct a substantial number of existing and/or missing gene models. Therefore, we believe that our careful manual curation ensures that the number of false positives and negatives are kept low and allows us to make statements about the presence/absence of particular genes.

### Phylogenetic reconstruction of KNOX, LEAFY, WOX and YABBY

For KNOX, AagrBONN.evm.model.Sc2ySwM.368.1986.6 was used as a query to BLASTp search at NCBI on 13 Sept 2019. The search database was NCBI non-redundant (nr) database limited to records that include: *A. thaliana*, *Oryza sativa* (japonica cultivar-group), *Phalaenopsis equestris*, *Amborella trichopoda*, *Ceratopteris richardii*, *Selaginella moellendorffii*, *M. polymorpha*, *P. patens*, *K. nitens*, *Ostreococcus tauri* and *C. reinhardtii*. The search parameters were otherwise as default. The hit sequences were downloaded and combined with the *Anthoceros* KNOX sequences, then aligned with FFT-NS-2 in MAFFT v.7.427 (ref. ^105^). The alignment was manually inspected in Mesquite v.3.6 (ref. ^130^) and 149 well-conserved sites of 51 sequences were included. Phylogenetic analysis based on maximum likelihood (ML) was conducted in MEGA X^131^. The best-fitting model was chosen as LG+G+I using the FindBestProteinModel function. A total of 100 bootstrap replicates were performed to evaluate branch support. ‘ML Heuristic Method’ was set to ‘Subtree-Pruning-Regrafting – Extensive (SPR level 5)’ and ‘No. of Discrete Gamma Categories’ set to 5.

For LEAFY, AagrOXF evm.model.utg000049l.76.4 was used as a query to BLASTp search at NCBI on 30 August 2019. The search database was nr limited to records that include: *A. thaliana*, *O. sativa* (japonica cultivar-group), *P. equestris*, *A. trichopoda*, *P. radiata*, *P. armandii*, *P. abies*, *C. richardii*, *S. moellendorffii*, *M. polymorpha* and *P. patens*. The search parameters were otherwise as default. The hit sequences were downloaded and combined with the *Anthoceros* LEAFY sequence and AHJ90704.1, AHJ90706.1, AHJ90707.1 from Sayou et al.^132^, then aligned with FFT-NS-2 in MAFFT v.7.427 (ref. ^105^). The alignment was manually inspected and processed as described above to include 194 conserved sites of 20 sequences. Phylogenetic inference was done similarly as above but with LG selected as the best-fitting model.

For WOX, WOX genes in *Anthoceros* genomes were searched using the corresponding *A. thaliana*, *P. patens* and *M. polymorpha* proteins. Based on comparison among the three genomes, three gene models with excess intron predictions were manually revised and one model was added. AagrOXF_evm.model.utg000018l.552.1 was used as a query to BLASTp search at NCBI on 9 October 2019. The search database was nr limited to records that include: *A. thaliana*, *O. sativa* (japonica cultivar-group), *P. equestris*, *A. trichopoda*, *C. richardii*, *S. moellendorffii*, *M. polymorpha*, *P. patens*, *K. nitens* and *C. braunii*. Maximum target was set to 250 and the word size as 2. The search parameters were otherwise as default. The hit sequences were downloaded and combined with the *Anthoceros* WOX sequences, then aligned with einsi —maxiterate 1,000 in MAFFT v.7.429 (ref. ^105^). The alignment was manually inspected with Mesquite v.3.6 (ref. ^130^) and a matrix consisting of 58 included sites of 142 sequences was constructed. Sequences identical in the included region were treated as a single operational taxonomic unit (OTU) during the phylogenetic analysis. The best-fitting model was chosen as JTT with ProteinModelSelection8.pl. The maximum likelihood (ML) tree was inferred by RAxM^133^ with -f a -\# 100 -m PROTGAMMAJTT and supplying -p and -x from random number generator. Bootstrap samples were generated with seqboot from PHYLIP package v.3.697 (ref. ^134^) and RAxML was run for each of them.

For *YABBY*, the 107 OTU dataset from Finet et al.^135^ was downloaded from treebase and combined with *YABBY* genes from *Huperzia* and *Anthoceros*. The sequences were aligned using einsi of MAFFT v.7.450 (ref. ^105^). The aligned sequences were manually inspected with Mesquite and short sequences were removed and ambiguously aligned or gap containing sites were excluded. The best-fitting model was chosen as HIVB by ProteinModelSelection8.pl and ML tree search followed what was described for WOX.

### Phylogenetic reconstruction of stomata-related genes

An *Anthoceros* ICE/SCRM homologue sequence AagrBONN_evm.model.Sc2ySwM_368.1570.1 was used as a query to BLASTp search at NCBI on 7 October 2019. The search database was nr limited to records that include: *A. thaliana*, *O. sativa* (japonica cultivar-group), *P. equestris*, *A. trichopoda*, *S. moellendorffii*, *P. patens*, *M. polymorpha*, *C. braunii* and *K. nitens*. The word size was set to 2 and maximum target sequences as 250. The search parameters were otherwise set as the default. The hit sequences (100) were downloaded and combined with the *Anthoceros* ICE/SCRM sequences, then aligned with einsi —maxiterate 1,000 in MAFFT v.7.429 (ref. ^105^). The alignment was manually inspected with MacClade 4.08 and 123 well-conserved sites were included to result in alignment of 66 sequences. The sequences identical in the included region were treated as a single OTU during the phylogenetic analysis. The best-fitting model was chosen as JTTDCMUTF with ProteinModelSelection8.pl. The ML tree was inferred by RAxML with -f a -# 100 -m PROTGAMMAJTTDCMUTF and supplying -p and -x from random number generator. Bootstrap samples (1,000 replicates) were generated with seqboot from PHYLIP package v.3.697 (ref. ^134^) and RAxML^133^ was run for each of them. For ERECTA and TMM, the sequences of AagrOXF_evm.model.utg000083l.351.1 and AagrOXF_evm.model.utg000012l.100.1 were respectively used as the query and processed as in ICE/SCRM. Phylogenetic analyses were performed as for the ICE/SCRM case, but with LG selected as the best-fitting model. For the EPF and EPF-like gene family, we used the matrix compiled by Takata et al.^136^ and added the *Anthoceros* and *M. polymorpha* homologues. ML tree inference was done by IQ-TREE v.1.6.1 with 1,000 replicates of UltraFast Bootstraps^109^.

### Identification of orthologues to AMF symbiosis genes

Homologues to symbiotic genes were retrieved in 31 species covering the different plant lineages (Supplementary Table 11) using protein from the model plant *Medicago truncatula* and the tBLASTn v.2.9.0+ (ref. ^128^) with a threshold e-value of 1e^−10^. Sequences were aligned using MAFFT v.7.407 (ref. ^105^) with default parameters and alignments were cleaned using TrimAl v.1.4 (ref. ^137^) to remove positions with more than 20% of gaps. Resulting alignments were subjected to ML tree inference using IQ-TREE v.1.6.1 (ref. ^107^). Before ML analysis, the best-fitting evolutionary model was tested using ModelFinder^108^ and according to the Bayesian Information Criteria Branch support was tested using 10,000 replicates of UltraFast Bootstraps^109^. Trees were visualized with the iTOL platform v.4.4.2 (ref. ^138^).

### Phylogenetic reconstruction of LCIB

The orthogroup OG0009668 was identified as the *LCIB* gene family containing *C. reinhardtii LCIB*-*E* genes. Additional hornwort *LCIB* homologues were retrieved from the 1,000 plant transcriptome database^6^. To find other *LCIB* homologues, we ran BLASTp against the Phytozome database using both the *Anthoceros* and C. *reinhardtii* sequences as the query and no hit could be obtained. Gene phylogeny was reconstructed on the basis of the amino acid alignment done by MUSCLE^139^. IQ-TREE v.1.6.1 (ref. ^107^) was used to obtain the ML tree as outlined above.

### Reporting Summary

Further information on research design is available in the Nature Research Reporting Summary linked to this article.

## Supplementary information

Supplementary Information Supplementary Note and Figs. 1–18.

Reporting Summary

Supplementary Tables Supplementary Tables 1–18.

## Data Availability

All three genomes and their annotations can be accessed, browsed, searched and downloaded at https://www.hornworts.uzh.ch/en.html. All the raw sequences are deposited in the NCBI Sequence Read Archive under the BioProject PRJNA574424 and PRJNA574453, and to European Nucleotide Archive (ENA) under the study accessions PRJEB34763 and PRJEB34743 (Supplementary Tables 2 and 3). The genome assemblies, annotations (Submitted.zip) as well as alignment matrices and tree files (phylogeny_dataset.zip) can be found at Figshare: 10.6084/m9.figshare.9974999.

## References

[1] Morris JL (2018). The timescale of early land plant evolution. Proc. Natl Acad. Sci. USA.

[2] Nishiyama T (2004). Chloroplast phylogeny indicates that bryophytes are monophyletic. Mol. Biol. Evol..

[3] Wickett NJ (2014). Phylotranscriptomic analysis of the origin and early diversification of land plants. Proc. Natl Acad. Sci. USA.

[4] Puttick MN (2018). The interrelationships of land plants and the nature of the ancestral embryophyte. Curr. Biol..

[5] de Sousa F, Foster PG, Donoghue PCJ, Schneider H, Cox CJ (2019). Nuclear protein phylogenies support the monophyly of the three bryophyte groups (Bryophyta Schimp). New Phytol..

[6] One Thousand Plant Transcriptomes Initiative. (2019). One thousand plant transcriptomes and the phylogenomics of green plants. Nature.

[7] Rensing SA (2008). The *Physcomitrella* genome reveals evolutionary insights into the conquest of land by plants. Science.

[8] Bowman JL (2017). Insights into land plant evolution garnered from the *Marchantia polymorpha* genome. Cell.

[9] Renzaglia KS (1978). Comparative morphology and developmental anatomy of the Anthocerotophyta. J. Hattori Bot. Lab..

[10] Smith EC, Griffiths H (1996). A pyrenoid-based carbon-concentrating mechanism is present in terrestrial bryophytes of the class Anthocerotae. Planta.

[11] Li F-W, Villarreal Aguilar JC, Szövényi P (2017). Hornworts: an overlooked window into carbon-concentrating mechanisms. Trends Plant Sci..

[12] Qiu Y-L (2006). The deepest divergences in land plants inferred from phylogenomic evidence. Proc. Natl Acad. Sci. USA.

[13] Renzaglia KS, Villarreal Aguilar JC, Piatkowski BT, Lucas JR, Merced A (2017). Hornwort stomata: architecture and fate shared with 400-Million-year-old fossil plants without leaves. Plant Physiol..

[14] Renzaglia, K. S., Villarreal, J. C. & Duff, R. J. in *Bryophyte Biology* Vol. 2 (eds Goffinet, B. & Shaw, J.) 139–171 (Cambridge Univ. Press, 2009).

[15] Meeks JC (1998). Symbiosis between nitrogen-fixing cyanobacteria and plants. Bioscience.

[16] Meeks JC (2009). Physiological adaptations in nitrogen-fixing *Nostoc*–plant symbiotic associations. Microbiol. Monogr..

[17] Szövényi P (2015). Establishment of *Anthoceros agrestis* as a model species for studying the biology of hornworts. BMC Plant Biol..

[18] Bainard JD, Villarreal Aguilar JC (2013). Genome size increases in recently diverged hornwort clades. Genome.

[19] Hornworts (Anthocerotophyta). *University of Zurich* https://www.hornworts.uzh.ch/en.html (2020).

[20] Jiang J, Birchler JA, Parrott WA, Dawe RK (2003). A molecular view of plant centromeres. Trends Plant Sci..

[21] Lang D (2018). The *Physcomitrella patens* chromosome-scale assembly reveals moss genome structure and evolution. Plant J..

[22] Landis JB (2018). Impact of whole-genome duplication events on diversification rates in angiosperms. Am. J. Bot..

[23] Banks JA (2011). The *Selaginella* genome identifies genetic changes associated with the evolution of vascular plants. Science.

[24] Wilhelmsson PKI, Mühlich C, Ullrich KK, Rensing SA (2017). Comprehensive genome-wide classification reveals that many plant-specific transcription factors evolved in streptophyte algae. Genome Biol. Evol..

[25] Ligrone R, Duckett JG, Renzaglia KS (2012). The origin of the sporophyte shoot in land plants: a bryological perspective. Ann. Bot..

[26] Chater CCC, Caine RS, Fleming AJ, Gray JE (2017). Origins and evolution of stomatal development. Plant Physiol..

[27] Coudert Y, Novák O, Harrison CJ (2019). A KNOX-cytokinin regulatory module predates the origin of indeterminate vascular plants. Current Biology.

[28] Sakakibara K (2013). KNOX2 genes regulate the haploid-to-diploid morphological transition in land plants. Science.

[29] Arun A (2019). Convergent recruitment of TALE homeodomain life cycle regulators to direct sporophyte development in land plants and brown algae. eLife.

[30] Sakakibara K (2014). WOX13-like genes are required for reprogramming of leaf and protoplast cells into stem cells in the moss *Physcomitrella patens*. Development.

[31] Romera-Branchat M, Ripoll JJ, Yanofsky MF, Pelaz S (2013). The WOX 13 homeobox gene promotes replum formation in the *Arabidopsis thaliana* fruit. Plant J..

[32] Denis E (2017). WOX14 promotes bioactive gibberellin synthesis and vascular cell differentiation in *Arabidopsis*. Plant J..

[33] Tanahashi T, Sumikawa N, Kato M, Hasebe M (2005). Diversification of gene function: homologs of the floral regulator FLO/LFY control the first zygotic cell division in the moss *Physcomitrella patens*. Development.

[34] Lee LR, Bergmann DC (2019). The plant stomatal lineage at a glance. J. Cell Sci..

[35] Chater CC (2016). Origin and function of stomata in the moss *Physcomitrella patens*. Nat. Plants.

[36] Kosentka PZ, Overholt A, Maradiaga R, Mitoubsi O, Shpak ED (2019). EPFL signals in the boundary region of the SAM restrict its size and promote leaf initiation. Plant Physiol..

[37] Nishiyama T (2018). The *Chara* genome: secondary complexity and implications for plant terrestrialization. Cell.

[38] Hernandez-Garcia J, Briones-Moreno A (2019). Origin of gibberellin-dependent transcriptional regulation by molecular exploitation of a transactivation domain in DELLA proteins. Mol. Biol. Evol..

[39] Walker CH, Siu-Ting K, Taylor A, O’Connell MJ, Bennett T (2019). Strigolactone synthesis is ancestral in land plants, but canonical strigolactone signalling is a flowering plant innovation. BMC Biol..

[40] Remy W, Taylor TN, Hass H, Kerp H (1994). Four hundred-million-year-old vesicular arbuscular mycorrhizae. Proc. Natl Acad. Sci. USA.

[41] Strullu-Derrien C (2018). Fossil filamentous microorganisms associated with plants in early terrestrial environments. Curr. Opin. Plant Biol..

[42] MacLean AM, Bravo A, Harrison MJ (2017). Plant signaling and metabolic pathways enabling arbuscular mycorrhizal symbiosis. Plant Cell.

[43] Parniske M (2008). Arbuscular mycorrhiza: the mother of plant root endosymbioses. Nat. Rev. Microbiol..

[44] Delaux P-M (2015). Algal ancestor of land plants was preadapted for symbiosis. Proc. Natl Acad. Sci. USA.

[45] Delaux P-M (2014). Comparative phylogenomics uncovers the impact of symbiotic associations on host genome evolution. PLoS Genet..

[46] Adams DG, Duggan PS (2008). Cyanobacteria–bryophyte symbioses. J. Exp. Bot..

[47] Rousk K, Jones DL, DeLuca TH (2013). Moss–cyanobacteria associations as biogenic sources of nitrogen in boreal forest ecosystems. Front. Microbiol..

[48] Steinberg NA, Meeks JC (1991). Physiological sources of reductant for nitrogen-fixation activity in *Nostoc* sp. strain UCD 7801 in symbiotic association with *Anthoceros punctatus*. J. Bacteriol..

[49] Ekman M, Picossi S, Campbell EL, Meeks JC, Flores E (2013). A *Nostoc punctiforme* sugar transporter necessary to establish a cyanobacterium–plant symbiosis. Plant Physiol..

[50] An J (2019). A *Medicago truncatula* SWEET transporter implicated in arbuscule maintenance during arbuscular mycorrhizal symbiosis. New Phytol..

[51] Kistner C (2005). Seven *Lotus japonicus* genes required for transcriptional reprogramming of the root during fungal and bacterial symbiosis. Plant Cell.

[52] Takeda N, Sato S, Asamizu E, Tabata S, Parniske M (2009). Apoplastic plant subtilases support arbuscular mycorrhiza development in *Lotus japonicus*. Plant J..

[53] Fournier J (2018). Cell remodeling and subtilase gene expression in the actinorhizal plant *Discaria trinervis* highlight host orchestration of intercellular *Frankia* colonization. New Phytol..

[54] Ribeiro A, Akkermans AD, van Kammen A, Bisseling T, Pawlowski K (1995). A nodule-specific gene encoding a subtilisin-like protease is expressed in early stages of actinorhizal nodule development. Plant Cell.

[55] Svistoonoff S (2003). cg12 expression is specifically linked to infection of root hairs and cortical cells during *Casuarina glauca* and *Allocasuarina verticillata* actinorhizal nodule development. Mol. Plant Microbe Interact..

[56] Meyer MT, Whittaker C, Griffiths H (2017). The algal pyrenoid: key unanswered questions. J. Exp. Bot..

[57] Rae BD (2017). Progress and challenges of engineering a biophysical CO_2_-concentrating mechanism into higher plants. J. Exp. Bot..

[58] Villarreal Aguilar JC, Renner SS (2012). Hornwort pyrenoids, carbon-concentrating structures, evolved and were lost at least five times during the last 100 million years. Proc. Natl Acad. Sci. USA.

[59] Wang Y, Spalding MH (2014). LCIB in the *Chlamydomonas* CO_2_-concentrating mechanism. Photosyn. Res..

[60] Atkinson N (2016). Introducing an algal carbon‐concentrating mechanism into higher plants: location and incorporation of key components. Plant Biotechnol. J..

[61] Jin S (2016). Structural insights into the LCIB protein family reveals a new group of β-carbonic anhydrases. Proc. Natl Acad. Sci. USA.

[62] Hanson, D. T., Renzaglia, K. & Villarreal, J. C. in *Photosynthesis in Bryophytes and Early Land Plants* (eds Hanson, D. T. & Rice, S. K.) 95–111 (Springer, 2014).

[63] Li F-W (2018). Fern genomes elucidate land plant evolution and cyanobacterial symbioses. Nat. Plants.

[64] Hori K (2014). *Klebsormidium flaccidum* genome reveals primary factors for plant terrestrial adaptation. Nat. Commun..

[65] Cheng S (2019). Genomes of subaerial Zygnematophyceae provide insights into land plant evolution. Cell.

[66] VanBuren R (2018). Extreme haplotype variation in the desiccation-tolerant clubmoss *Selaginella lepidophylla*. Nat. Commun..

[67] Cove DJ (2009). Culturing the moss *Physcomitrella patens*. Cold Spring Harb. Protoc..

[68] Hatcher RE (1965). Towards the establishment of a pure culture collection of Hepaticae. Bryologist.

[69] Nagar, R. & Schwessinger, B. High purity, high molecular weight DNA extraction from rust spores via CTAB based DNA precipitation for long read sequencing v1. *protocols.io*10.17504/protocols.io.n5ydg7w (2018).

[70] Weisenfeld NI (2014). Comprehensive variation discovery in single human genomes. Nat. Genet..

[71] Chen S, Zhou Y, Chen Y, Gu J (2018). fastp: an ultra-fast all-in-one FASTQ preprocessor. Bioinformatics.

[72] Li H, Durbin R (2009). Fast and accurate short read alignment with Burrows–Wheeler transform. Bioinformatics.

[73] Li H (2009). The sequence alignment/map format and SAMtools. Bioinformatics.

[74] Okonechnikov K, Conesa A, García-Alcalde F (2016). Qualimap 2: advanced multi-sample quality control for high-throughput sequencing data. Bioinformatics.

[75] Marçais G, Kingsford C (2011). A fast, lock-free approach for efficient parallel counting of occurrences of *k*-mers. Bioinformatics.

[76] Vurture GW (2017). GenomeScope: fast reference-free genome profiling from short reads. Bioinformatics.

[77] Zimin AV (2013). The MaSuRCA genome assembler. Bioinformatics.

[78] Walker BJ (2014). Pilon: an integrated tool for comprehensive microbial variant detection and genome assembly improvement. PLoS ONE.

[79] Putnam NH (2016). Chromosome-scale shotgun assembly using an in vitro method for long-range linkage. Genome Res..

[80] Li H (2016). Minimap and miniasm: fast mapping and de novo assembly for noisy long sequences. Bioinformatics.

[81] Vaser R, Sović I, Nagarajan N, Šikić M (2017). Fast and accurate de novo genome assembly from long uncorrected reads. Genome Res..

[82] Laetsch DR, Blaxter ML (2017). BlobTools: interrogation of genome assemblies. F1000Res..

[83] Bolger AM, Lohse M, Usadel B (2014). Trimmomatic: a flexible trimmer for Illumina sequence data. Bioinformatics.

[84] Patro R, Duggal G, Love MI, Irizarry RA, Kingsford C (2017). Salmon provides fast and bias-aware quantification of transcript expression. Nat. Methods.

[85] Love MI, Huber W, Anders S (2014). Moderated estimation of fold change and dispersion for RNA-seq data with DESeq2. Genome Biol..

[86] Enderlin CS, Meeks JC (1983). Pure culture and reconstitution of the *Anthoceros–Nostoc* symbiotic association. Planta.

[87] Kim D, Langmead B, Salzberg SL (2015). HISAT: a fast spliced aligner with low memory requirements. Nat. Methods.

[88] Pertea M (2015). StringTie enables improved reconstruction of a transcriptome from RNA-seq reads. Nat. Biotechnol..

[89] Maere S, Heymans K, Kuiper M (2005). BiNGO: a Cytoscape plugin to assess overrepresentation of gene ontology categories in biological networks. Bioinformatics.

[90] Supek F, Bošnjak M, Škunca N, Šmuc T (2011). REVIGO summarizes and visualizes long lists of gene ontology terms. PLoS ONE.

[91] Smit, A. F. A. & Hubley, R. *RepeatModeler Open-1.0* (Institute for Systems Biology, accessed February 2019); www.repeatmasker.org

[92] Ou S, Jiang N (2018). LTR_retriever: a highly accurate and sensitive program for identification of long terminal repeat retrotransposons. Plant Physiol..

[93] Smit, A. F. A., Hubley, R. & Green, P. *RepeatMasker Open-4.0* (Institute for Systems Biology, accessed February 2019); www.repeatmasker.org

[94] Mapleson D, Venturini L, Kaithakottil G, Swarbreck D (2018). Efficient and accurate detection of splice junctions from RNA-seq with Portcullis. Gigascience.

[95] Grabherr MG (2011). Full-length transcriptome assembly from RNA-Seq data without a reference genome. Nat. Biotechnol..

[96] Haas BJ (2003). Improving the *Arabidopsis* genome annotation using maximal transcript alignment assemblies. Nucleic Acids Res..

[97] Venturini L, Caim S, Kaithakottil GG, Mapleson DL, Swarbreck D (2018). Leveraging multiple transcriptome assembly methods for improved gene structure annotation. Gigascience.

[98] Slater GSC, Birney E (2005). Automated generation of heuristics for biological sequence comparison. BMC Bioinf..

[99] Stanke M, Morgenstern B (2005). AUGUSTUS: a web server for gene prediction in eukaryotes that allows user-defined constraints. Nucleic Acids Res..

[100] Hoff KJ, Lange S, Lomsadze A, Borodovsky M, Stanke M (2016). BRAKER1: unsupervised RNA-seq-based genome annotation with GeneMark-ET and AUGUSTUS. Bioinformatics.

[101] Haas BJ (2008). Automated eukaryotic gene structure annotation using EVidenceModeler and the program to assemble spliced alignments. Genome Biol..

[102] Angiuoli SV, Salzberg SL (2011). Mugsy: fast multiple alignment of closely related whole genomes. Bioinformatics.

[103] Simão FA, Waterhouse RM, Ioannidis P, Kriventseva EV, Zdobnov EM (2015). BUSCO: assessing genome assembly and annotation completeness with single-copy orthologs. Bioinformatics.

[104] Emms DM, Kelly S (2019). OrthoFinder: phylogenetic orthology inference for comparative genomics. Genome Biol..

[105] Katoh K, Standley DM (2013). MAFFT multiple sequence alignment software version 7: improvements in performance and usability. Mol. Biol. Evol..

[106] Abascal F, Zardoya R, Telford MJ (2010). TranslatorX: multiple alignment of nucleotide sequences guided by amino acid translations. Nucleic Acids Res..

[107] Nguyen L-T, Schmidt HA, von Haeseler A, Minh BQ (2015). IQ-TREE: a fast and effective stochastic algorithm for estimating maximum-likelihood phylogenies. Mol. Biol. Evol..

[108] Kalyaanamoorthy S, Minh BQ, Wong TKF, von Haeseler A, Jermiin LS (2017). ModelFinder: fast model selection for accurate phylogenetic estimates. Nat. Methods.

[109] Hoang DT, Chernomor O, von Haeseler A, Minh BQ, Vinh LS (2018). UFBoot2: improving the ultrafast bootstrap approximation. Mol. Biol. Evol..

[110] Guindon S (2010). New algorithms and methods to estimate maximum-likelihood phylogenies: assessing the performance of PhyML 3.0. Syst. Biol..

[111] Zhang C, Rabiee M, Sayyari E, Mirarab S (2018). ASTRAL-III: polynomial time species tree reconstruction from partially resolved gene trees. BMC Bioinf..

[112] Sayyari E, Mirarab S (2016). Fast coalescent-based computation of local branch support from quartet frequencies. Mol. Biol. Evol..

[113] Sayyari E, Whitfield JB, Mirarab S (2018). DiscoVista: interpretable visualizations of gene tree discordance. Mol. Phylogenet. Evol..

[114] Cabanettes F, Klopp C (2018). D-GENIES: dot plot large genomes in an interactive, efficient and simple way. PeerJ..

[115] Marçais G (2018). MUMmer4: a fast and versatile genome alignment system. PLoS Comput. Biol..

[116] Nattestad M, Schatz MC (2016). Assemblytics: a web analytics tool for the detection of variants from an assembly. Bioinformatics.

[117] Qiao X (2019). Gene duplication and evolution in recurring polyploidization–diploidization cycles in plants. Genome Biol..

[118] Goodstein DM (2012). Phytozome: a comparative platform for green plant genomics. Nucleic Acids Res..

[119] Proost S (2012). i-ADHoRe 3.0—fast and sensitive detection of genomic homology in extremely large data sets. Nucleic Acids Res..

[120] Benson G (1999). Tandem repeats finder: a program to analyze DNA sequences. Nucleic Acids Res..

[121] Barker MS (2010). EvoPipes.net: bioinformatic tools for ecological and evolutionary genomics. Evol. Bioinform..

[122] Birney E, Clamp M, Durbin R (2004). Genewise and genomewise. Genome Res..

[123] Yang Z (2007). PAML 4: phylogenetic analysis by maximum likelihood. Mol. Biol. Evol..

[124] Tang H (2008). Unraveling ancient hexaploidy through multiply-aligned angiosperm gene maps. Genome Res..

[125] Mitchell AL (2019). InterPro in 2019: improving coverage, classification and access to protein sequence annotations. Nucleic Acids Res..

[126] El-Gebali S (2019). The Pfam protein families database in 2019. Nucleic Acids Res..

[127] Marchler-Bauer A (2017). CDD/SPARCLE: functional classification of proteins via subfamily domain architectures. Nucleic Acids Res..

[128] Camacho C (2009). BLAST+: architecture and applications. BMC Bioinf..

[129] Wheeler TJ, Eddy SR (2013). Nhmmer: DNA homology search with profile HMMs. Bioinformatics.

[130] Maddison, W. P. & Maddison, D. R. *Mesquite: A Modular System for Evolutionary Analysis* v.3.04 (Mesquite, accessed 5 July 2016); http://mesquiteproject.org

[131] Kumar S, Stecher G, Li M, Knyaz C, Tamura K (2018). MEGA X: molecular evolutionary genetics analysis across computing platforms. Mol. Biol. Evol..

[132] Sayou C (2014). A promiscuous intermediate underlies the evolution of LEAFY DNA binding specificity. Science.

[133] Stamatakis A (2014). RAxML version 8: a tool for phylogenetic analysis and post-analysis of large phylogenies. Bioinformatics.

[134] Felsenstein, J. *PHYLIP (Phylogeny Inference Package)* Version 3.697 (University of Washington, 2015); http://evolution.genetics.washington.edu/phylip/oldversions.html

[135] Finet C (2016). Evolution of the YABBY gene family in seed plants. Evol. Dev..

[136] Takata N (2013). Evolutionary relationship and structural characterization of the EPF/EPFL gene family. PLoS ONE.

[137] Capella-Gutierrez S, Silla-Martinez JM, Gabaldon T (2009). trimAl: a tool for automated alignment trimming in large-scale phylogenetic analyses. Bioinformatics.

[138] Letunic I, Bork P (2016). Interactive tree of life (iTOL) v3: an online tool for the display and annotation of phylogenetic and other trees. Nucleic Acids Res..

[139] Edgar RC (2004). MUSCLE: multiple sequence alignment with high accuracy and high throughput. Nucleic Acids Res..

