# Supplementary material for: Anthoceros genomes illuminate the origin of land plants and the unique biology of hornworts
Source: Nat Plants. 2020 Mar 13;6(3):259–72. doi: 10.1038/s41477-020-0618-2 (PMC8075897; doi:10.1038/s41477-020-0618-2)
Supplement: Supplementary file 1 — Supplementary Note and Figs. 1–18. [file 41477_2020_618_MOESM1_ESM.pdf]

nature plants

SUPPLEMENTARY INFORMATION

https://doi.org/10.1038/s41477-020-0618-2

In the format provided by the authors and unedited.

Anthoceros genomes illuminate the origin of land plants and the unique biology of hornworts

Fay-Wei Li[1,2] ✉, Tomoaki Nishiyama[3], Manuel Waller[4], Eftychios Frangedakis[5], Jean Keller[6], Zheng Li[7], Noe Fernandez-Pozo[8], Michael S. Barker[7], Tom Bennett[9], Miguel A. Blázquez[10], Shifeng Cheng[11], Andrew C. Cuming[9], Jan de Vries[12], Sophie de Vries[13], Pierre-Marc Delaux[6], Issa S. Diop[4], C. Jill Harrison[14], Duncan Hauser[1], Jorge Hernández-García[10], Alexander Kirbis[4], John C. Meeks[15], Isabel Monte[16], Sumanth K. Mutte[17], Anna Neubauer[4], Dietmar Quandt[18], Tanner Robison[1,2], Masaki Shimamura[19], Stefan A. Rensing[8,20,21], Juan Carlos Villarreal[22,23], Dolf Weijers[17], Susann Wicke[24], Gane K.-S. Wong[25,26], Keiko Sakakibara[27] and Péter Szövényi[4,28] ✉

[1]Boyce Thompson Institute, Ithaca, NY, USA. [2]Plant Biology Section, Cornell University, Ithaca, NY, USA. [3]Advanced Science Research Center, Kanazawa University, Ishikawa, Japan. [4]Department of Systematic and Evolutionary Botany, University of Zurich, Zurich, Switzerland. [5]Department of Plant Sciences, University of Cambridge, Cambridge, UK. [6]LRSV, Université de Toulouse, CNRS, UPS Castanet-Tolosan, Toulouse, France. [7]Department of Ecology and Evolutionary Biology, University of Arizona, Tucson, AZ, USA. [8]Faculty of Biology, Philipps University of Marburg, Marburg, Germany. [9]Centre for Plant Sciences, Faculty of Biological Sciences, University of Leeds, Leeds, UK. [10]Instituto de Biología Molecular y Celular de Plantas, CSIC-Universidad Politécnica de Valencia, Valencia, Spain. [11]Shenzhen Branch, Guangdong Laboratory for Lingnan Modern Agriculture, Genome Analysis Laboratory of the Ministry of Agriculture, Agricultural Genomics Institute at Shenzhen, Chinese Academy of Agricultural Sciences, Shenzhen, China. [12]Institute for Microbiology and Genetics, Department of Applied Bioinformatics, Georg-August University Göttingen, Göttingen, Germany. [13]Institute of Population Genetics, Heinrich Heine University Düsseldorf, Düsseldorf, Germany. [14]School of Biological Sciences, University of Bristol, Bristol, UK. [15]Department of Microbiology and Molecular Genetics, University of California, Davis, CA, USA. [16]Department of Plant and Microbial Biology, University of Zurich, Zurich, Switzerland. [17]Laboratory of Biochemistry, Wageningen University & Research, Wageningen, the Netherlands. [18]Nees Institute for Biodiversity of Plants, University of Bonn, Bonn, Germany. [19]Graduate School of Integrated Sciences for Life, Hiroshima University, Hiroshima, Japan. [20]BIOSS Centre for Biological Signalling Studies, University of Freiburg, Freiburg, Germany. [21]LOEWE Center for Synthetic Microbiology (SYNMIKRO), University of Marburg, Marburg, Germany. [22]Department of Biology, Laval University, Quebec City, Quebec, Canada. [23]Smithsonian Tropical Research Institute, Balboa, Panamá. [24]Institute for Evolution and Biodiversity, University of Muenster, Münster, Germany. [25]Department of Biological Sciences, Department of Medicine, University of Alberta, Edmonton, Alberta, Canada. [26]BGI-Shenzhen, Shenzhen, China. [27]Department of Life Science, Rikkyo University, Tokyo, Japan. [28]Zurich-Basel Plant Science Center, Zurich, Switzerland. ✉e-mail: fl329@cornell.edu; peter.szoevenyi@systbot.uzh.ch

Supplementary Notes

Table of content

Read mapping statistics 1

Centromeres and repeats 2

Strain-specific genes of *A. agrestis* and the pan genome 3

Transcription associated proteins (TAPs) 4

Genes related to meristem development 4

Genes related to stomatal development 5

Abscisic acid (ABA) signaling and synthesis 6

Auxin signaling and synthesis 7

Jasmonates 8

Strigolactones 8

Gibberellic acid (GA) signaling and synthesis 9

Salicylic acid 10

Ethylene and cytokinin signaling/synthesis 13

Genes for plastidial peptidoglycan biosynthesis 13

Monoplastidy 14

Plastid targeted proteins 15

Photoreceptors 15

Literature cited 16

Read mapping statistics

Supplementary Table 2 reports detailed read mapping statistics of Illumina genomic reads. We believe the high mapping rates (95.1%-99.4%) are correct for the following reasons. First, while a number of contigs/scaffolds were removed as contaminants, the vast majority of them have very low or zero Illumina read coverage (see Supplementary Table 14) and therefore did not impact much on the Illumina read mapping statistics. Second, as shown in the revised Supplementary Table 2, it is the trimmed reads that achieved >99% mapping rates, after the low-quality bases were removed.

We did notice that *A. agrestis* Oxford and *A. punctatus* Illumina reads have a lower genomic coverage (84.6%-87.1%), compared to *A. agrestis* Bonn (99.8%). This is likely reflecting the different assemblers we used for these genomes: minimap2-miniasm-racon-pilon for the

former two and masurca for the latter. Masurca is a hybrid assembler that combines Illumina and nanopore reads early in the assembly process to make mega-reads, whereas the minimap2-miniasm-racon-pilon approach only uses Illumina reads for polishing after the contigs were already constructed. The minimap assemblies may have captured more regions that are not recovered in the Masurca assembly, though It is likely that the minimap assemblies still may have residual errors especially in the repeat regions where Illumina reads can not be placed with confidence and hence exhibit lower coverage when Illumina reads were mapped back to them. Conversely, *A. agrestis* Bonn has a lower proportion of uniquely mapped reads (i.e. less reads with high mapping qualities) than *A. agrestis* Oxford and *A. punctatus*. This again might be due to the higher accuracy in the repeat regions in *A. agrestis* Bonn, causing more reads to be multi-mapped.

Centromeres and repeats

We could not find typical vascular plant centromeric regions in the *Anthoceros* genomes. First, when mapping the only Illumina-based assembly to the chromosomal-scale assembly, there were no indication of obvious DNA stretches missing from the Illumina assembly but present in the chromosomal-scale assembly (Supplementary Figure 5). Second, we could not identify stretches of the putative chromosomes with elevated repeat content and similarity to the other chromosomes typical for vascular plant centromeres (Supplementary Figures 4-5). Finally, we could not find typical centromeric repeats containing long tandem repeat arrays (100-1000 repeat unit in length) with a period length greater than 50bp (Supplementary Figure 5). Repeats with a period length greater than 50 bp built up very short arrays. When reducing the period length threshold to a minimum of 10 bp, we found that there was one longest tandem array on most of the scaffolds that may mark the position of centromeres (Supplementary Figure 5). Nevertheless, these arrays were still short and not considerably longer than other arrays. Therefore, extensive centromeric/pericentromeric regions with abundant long tandem repeats are missing from the *A. agrestis* genome.

We found that gene density is low but repeat and transposon density is high at the very end of the scaffolds. Nevertheless, the conserved “TTTAGGG” motif[4] typical for plant telomeres occurred only on four scaffolds in short tandem arrays (maximum array length of 13 repeat units) and usually sub-terminally (Supplementary Table 17). Similarly, the less common telomeric repeats described for plants and algae, such as the one found in *Chlamydomonas* [TTTTTAGGG][5], *Cestrum* [TTTTTTAGGG][6], and *Allium* [CTCGGTTATGGG][7] were missing from the genome. Nevertheless, we found that tandem repeats with a unit length between 5-15bp and array length greater than 20 repeats are numerous at the end of the scaffolds (1Mbp from the end of the scaffolds) but only shorter tandem arrays are terminal whereas longer arrays are rather sub-terminally localized (Supplementary Table 16). Such repeats may be part of the telomeres but are not specifically restricted to the telomeric regions and can also occur in the inner parts of the scaffolds. Finally, nucleotide sequence of these putative telomeric tandem arrays is not fixed and varies considerably across scaffolds (Supplementary Table 17).

Altogether, our analyses of the *A. agrestis* Bonn genome suggest that most of the telomeres are either missing from the current assembly or their structure/repeat composition is more variable than previously thought. In addition, hornwort centromeres may not be

characterized by considerably higher repeat density than other parts of the genome and their putative tandem repeat arrays may be shorter than those of vascular plants. Similar centromere and telomere structure was discovered in the moss, *P. patens*, one of the other two monophyletic lineages of bryophytes[8]. Therefore, it is possible that this genome structure is a shared feature of the group of bryophytes as a whole. Our assemblies are based on long reads, which makes it unlikely that our findings (i.e. shorter tandem repeats than in typical flowering plant centromeres) are due to assembly problems. Nevertheless, this possibility cannot be completely ruled out and has to be addressed in the future.

Strain-specific genes of *A. agrestis* and the pan genome

Our combined individual and comparative annotation approach resulted in approximately 1000 more gene models in the Bonn strain than in the Oxford strain of *A. agrestis*. Here we carried out further analyses to show that this difference is not due to annotation issues. To do so, we first defined whether these ~1000 gene models are specific for the Bonn strain`s gene set. Using BLASTn[9] searches (e-value threshold of 10^{-6}, alignment similarity of 80% and 50% coverage) we found that primary transcripts of 1131 genes of the Bonn strain (4.41%) had no homologs in the Oxford strain's predicted gene set (Supplementary Table 5). This shows that almost all of the approximately 1000 additionally predicted gene models of the Bonn genome seem to be strain-specific. After that we investigated whether the majority of the 1131 putatively strain-specific genes occur on strain-specific genomic regions. We run the nucmer and dnadiff modules of MUMmer4[10] to identify strain-specific genomic regions. We further verified the strain-specificity of the detected regions using BLASTn with a threshold of 90% similarity and 80% coverage. We found that 2,431,441 base pairs of the Bonn strain's genome was strain-specific. These genomic regions housed 567 strain-specific gene models. Altogether, this shows that half (50.13%) of the putatively strain-specific gene models is restricted to the Bonn strain's genome and are not due to annotation issues. Finally, we searched the primary transcripts of the putatively strain-specific Bonn loci that were not located on the strain-specific genomic regions using blat[11] (threshold: min 90% similarity, bestoverlap, min length coverage 50%) against the Oxford genome`s sequence. We found that using the cutoff only 12% (68) of the 564 primary transcripts had a valid alignment against the Oxford strain's genome. This shows that most of these gene models (their sequence) is only partially present in the Oxford strain`s genome which can well explain their absence in the predicted gene set. Altogether, our analysis shows that genome annotations of the two *A. agrestis* strains are largely comparable and the gene number difference is real and not a consequence of annotation issues.

To aid future comparative analyses we created an *A. agrestis* pan genome containing a non-redundant set of genomic sequences and annotations of the two strains. To do so, we first identified genomic regions that were absent in one or the other strain`s genome using the nucmer module of MUMmer4[10] which we further verified using BLASTn searches with a threshold of 90% coverage and 95% similarity. For this analysis we used the Oxford strain as the reference because the Bonn strain had a larger number of predicted genes which we wanted to add to the pan genome. In the next step we added strain-specific sequence regions from Bonn to the Oxford genome together with their annotations. To consolidate

gene models we next mapped all the Bonn gene models against the draft pan-genome using blat[11] and accepted mappings with at least 80% similarity. We used this mapping file to retain all original Oxford gene models showing at least 80% overlap with the mapped gene models of the Bonn strain. The Bonn gene models that did not meet these criteria (i.e. no overlap with the Bonn annotation) were added to the assembly as new genes. Finally, all sequence regions categorized as missing from the Oxford assembly were concatenated (chunks separated by 100 Ns) and added to the Oxford genome fasta file to provide a pan-genome assembly file. Annotations were also merged into a single pan-genome gff3 file. The pan genome is 125.8 Mbp long and contains 25,309 gene models and can be retrieved from Figshare (http://dx.doi.org/10.6084/m9.figshare.9974999) as "Pangenome_Subm.zip". Descriptive statistics of the pan genome are provided in Supplementary Table 5.

Transcription associated proteins (TAPs)

We found that there are several lineage-specific TAP losses among bryophytes (Supplementary Table 8). Type I MADS box genes are present in both *P. patens* and the hornwort genomes but are missing from *M. polymorpha*. On the other hand, some TAP families were uniquely lost in hornworts. For instance, SHI RELATED SEQUENCE (SRS) TFs originated in the most recent common ancestor (MRCA) of Streptophytes[12] and are present in *Chara*, liverworts, mosses, and vascular plants but are missing from the hornwort genomes (Supplementary Table 8). C2C2-YABBY transcription factor represents yet another evolutionary pattern. *YABBY* homologs are missing from the *P. patens* and *M. polymorpha* genomes but encoded by the hornwort genomes (Supplementary Tables 8-9). Therefore, *YABBY* likely evolved in the MRCA of land plants, were retained in all seed plants, *Huperzia*[13], and hornworts, but got lost in mosses, liverworts, ferns, and *Selaginella*[13]. *YABBY* functions in abaxial side of lateral organs in seed plants[14] and expressed in early sporophyte stages in hornworts (Fig. 3). Further functional study in hornworts may shed light on its function.

Genes related to meristem development

WUSCHEL, is a WOX gene family member and regulates meristem size in the *A. thaliana* shoot apical meristem by communicating with a receptor kinase pathway composed of two leucine-rich repeat (LRR) receptor kinases, *CLAVATA1* (*CLV1*), *CLAVATA2* (*CLV2*) and their ligand *CLV3*, a small secreted protein[15]. The CLV3-CLV1 pathway seems to be functional in mosses and regulate division plane orientation in gametophyte meristems[16]. Similarly, proliferation of gametophytic meristems in *M. polymorpha* appears to be affected by CLE peptide hormones[17]. Presence of a CLE domain containing gene as well as a *CLV1* homolog in the hornwort genomes suggests the existence of this pathway in land plant MRCA (Supplementary Table 10). This finding implies that division plane regulation/regulation of proliferation might be an ancient function of CLE peptides, which was

likely present in the land plant MRCA. Whether the CLE-receptor pathway is only active in the gametophyte or potentially also in the sporophyte meristem remains to be investigated.

Transcription factor *LEAFY/FLORICAULA* (LFY/FLO)[18] and *WUSCHEL-related homeobox 13 like* (WOX13L)[19] genes are involved in the zygote development in the moss *P. patens* and shoot apical meristem (SAM) maintenance in flowering plants[20]. The hornwort genomes encode a single *LFY* gene and four *WOX13L* orthologs (*WOX13La*, *WOX13Lb*, *WOX13Lc*, and *WOX13Ld*; Supplementary Figure 8; Supplementary Table 9). *WOX13La* and *b* resulted from a recent duplication and together are closely related to *WOX13Lc*. *WOX13Ld* on the other hand is more divergent (Supplementary Figure 8). *WOX13L* genes regulate zygote division and are necessary for cell reprogramming in *P. patens*, while *WOX13* and *WOX14* genes are involved in fruit development and vascular cell differentiation in *A. thaliana*[19,21,22]. Preferential sporophyte expression of the hornwort *WOX13La* suggests that it is likely involved in sporophytic meristem maintenance. In contrast, *WOXL13Lb* and *c* are expressed both in the early gametophyte and late sporophyte developmental stages, perhaps regulating early rhizoid development in the gametophyte and dehiscence of the sporophyte similarly to the *WOX13* genes of *A. thaliana*[22]. *WOX13Ld* is also expressed in both phases (gametophyte and sporophyte) of the life cycle but mainly in advanced stages of development with unknown functions. Altogether, our expression data suggests possible subfunctionalization and dual role of the four *WOX13L* genes in hornworts and implies some functional conservation with their *A. thaliana* and *P. patens* orthologs.

The *A. agrestis* LFY gene was 61% identical to the published *Nothoceros aenigmaticus* LFY sequence and amino acid position critical to DNA binding affinity was identical (Supplementary Figure 8), which have unique DNA binding specificity different from *Klebsormidium nitens*, mosses, liverworts and vascular plants[23]. It was shown that hornwort LFY has promiscuous DNA-binding ability which may have allowed it to evolve new binding specificity[23]. Nevertheless, biological function of LFY in hornworts is completely unexplored. Our expression data, in contrast to what was described for *P. patens*[18], showed that the hornwort LFY is primarily expressed in the gametophyte and not in the sporophyte phase (Fig. 3). This expression pattern may imply that the promiscuous DNA binding domain of the hornwort LFY is associated with radically new functions which need to be explored in the future.

Genes related to stomatal development

In *A. thaliana,* stomatal development and patterning is controlled by a well-defined set of genes[24,25]. This is composed of the basic helix-loop-helix (bHLH) transcription factors *SPEECHLESS (SPCH), MUTE, FAMA*, and *ICE/SCREAMs (SCRMs),* all contributing to stomata development. These genes act in a stomatal signaling cascade including the mobile peptide *EPIDERMAL PATTERNING FACTOR (EPF), ERECTA,* and *TOO MANY MOUTHS* (*TMM*)*,* which determines how stomata are spatially arranged. Stomata occur in hornworts, mosses and all vascular plants, but is absent in liverworts[26]. It was suggested that stomata may have evolved once in the common ancestor of land plants and was independently lost in liverworts[27]. Using stomatal toolbox genes of *A. thaliana* and *P. patens* and BLASTp searches we found that orthologs of all these genes are present in the three hornwort

genomes (Supplementary Table 10; Supplementary Figure 9). This is in line with a previous study using an earlier draft assembly of our genomes[26], and supports the hypothesis that stomata and their regulation have evolved once and relies on a common set of highly conserved genes.

Previous studies on *P. patens* suggested that *FAMA* and *SCRM* functions are likely conserved between the moss and *A. thaliana* by creating stomatal lineage cells and triggering differentiation of guard cells[28]. We found that the hornwort orthologs of *FAMA* and *SCRM* were both strongly and preferentially expressed in the early stages of sporophyte development (Fig. 3). This finding suggests functional conservation of these two genes across *P. patens*, hornworts and *A. thaliana*. The function and action of *EPF*, *TMM* and *ERECTA* genes in *P. patens* is not known but they may regulate stomatal spacing via the *FAMA* orthologs, similar to *A. thaliana*[26]. In hornworts, we found that *TMM* and *EPF* were preferentially expressed in the early stages of sporophyte development. This is consistent with the idea that *TMM* and *EPF* has a deeply conserved function in regulating stomatal development, probably stomatal spacing, across land plants. Although *ERECTA* showed increased expression in the early stages of sporophyte development, it was also strongly expressed in one of the gametophyte samples. Sporophytic expression of *ERECTA* is in line with its functional conservation in stomatal development, but its gametophytic expression needs to be further investigated. Taken together, while our data strongly support the involvement of *FAMA*, *SCRM, EPF,* and *TMM* in hornwort stomatal development, the role of *ERECTA* remains ambiguous. These observations provide strong evidence for a remarkable conservation of the developmental toolbox of stomatal development spanning more than 400 million years of evolution from the bryophytes to flowering plants.

Abscisic acid (ABA) signaling and synthesis

Dormancy and stress acclimation is regulated by the plant hormone Abscisic Acid (ABA) under water limited conditions. Here we focused on the ABA response pathway[29,30]. ABA binding with receptor promotes interaction of PYLs with the active site of PP2C phosphatases subclass A (encoded by *Arabidopsis* genes ABI1, ABI2, HAB1). This prevents dephosphorylation of SnRK2 protein kinases (Subclass III), thereby enabling them to proceed to phosphorylate target proteins that execute the ABA response. Non-vascular plants contain an additional protein kinase, encoded in *P. patens* by the ANR gene (for the ABA Non-Responsive mutant phenotype)[31].

Executive proteins in the ABA response include the bZIP transcription factors ABI5, ABF1-4, AREB1-3, DPBF1-2, and the B3 transcription factor ABI3. In guard cells, the SLAC1 slow ion channel protein that initiates ABA-mediated stomatal closure is also a target of SnRK2 phosphorylation[32]. We also searched for genes part of the Carotenoid and ABA biosynthesis pathway and for some genes regulated by ABA (LEA proteins)[33]. We used *P. patens* and *M. polymorpha* protein sequences to search for homologs in the hornwort assemblies using the tBLASTn search tool[9].

In general, we found that the ABA-related gene content of *A. agrestis* is similar to those described from the *M. polymorpha* and *P. patens* genomes[12,34] (Supplementary Table 10).

We found one ABA receptor homolog in the *A. agrestis* genome. Homologs of the putative phosphatase and kinase genes were also all present as well as a homolog of the non-vascular plant specific ANR kinase. Of special importance is the SnRK2 kinase that activates ABA transcriptional responses by phosphorylating the bZip transcription factors, which bind the ABA Response Element (ABRE) in the promoters of ABA-regulated genes. This gene also phosphorylates the SLAC1 Slow Anion Channel protein that mediates stomatal closure. Homologs of both SnRK2 and SLAC1 were present in the genome (Supplementary Table 10).

Of the executive bZip transcription factors, there seems to be only one that is orthologous with the ABA response element binding class, and contains the consensus SnRK2 phosphorylation sites (Supplementary Table 10). We also found multiple B3 transcription factors homolog to ABI3. Our analysis revealed that genes of enzymes critical for the biosynthetic pathway of ABA production are present in the hornwort genome and multiple members of the ABA responsive LEA proteins also occur in the genome (Supplementary Table 10). Therefore, the ABA sensing, response pathway, and its synthesis appears to be similar to what was described earlier for the other two bryophyte species[12,34].

Auxin signaling and synthesis

To identify the orthologues of genes involved in auxin synthesis and signaling in the *Anthoceros* genomes, protein sequences of the corresponding genes from *A. thaliana* (TAIR10 genome with Araport11 annotations) and *M. polymorpha* (v3.1 from Phytozome) were used. Initially, the protein sequences from these two species were queried to each of the *Anthoceros* proteomes using BLASTp with the criteria of E-value less than 0.001[9]. Then the protein sequences for all the corresponding hits from each BLAST result, were queried back to the *Arabidopsis* proteome to confirm the orthologous relationship with the respective gene families using best-bidirectional BLAST hits strategy. The domain information for these orthologues was confirmed using InterProScan[35]. The list of *Anthoceros* orthologs with best *A. thaliana* hit are provided in Supplementary Table 10. We found representatives of the auxin biosynthesis genes[36], Tryptophan amino transferase (TAA), and YUCCA family of flavin monooxygenases (YUC), in all three genomes investigated. Total of 2-3 TAA gene homologs were recovered from the genomes that is slightly more than the single copy discovered in *M. polymorpha*[12,34]. Furthermore, the three hornwort genomes contained a large repertoire of YUCCA homologs outnumbering those found in the paralog-poor genome of *M. polymorpha*. This observation confirms that the conserved land plant auxin biosynthetic pathway using indole-3-pyruvic acid is present in all three lineages of bryophytes. The transcriptional response machinery consists of five ARF genes, two AUX/IAA homologs, and one to three TIR1 homolog (Supplementary Table 10). In which tissues and in what temporal manner these genes are expressed in hornworts needs to be further investigated.

Jasmonates

Plant reactions to abiotic and biotic stresses usually involves the jasmonate (JA) signaling pathway[37,38]. Core components of the JA signaling pathway appears to be present both in *P. patens* and *M. polymorpha*. JAZ proteins are repressors that simultaneously bind to the NINJA adaptor protein and to several transcription factors (TF), which recruits the TOPLESS repressor (TPL). When JA is present, JAZ protein binds to the SCFCOI1 (Skp–Cullin–F-box) complex by forming a co-receptor with COI1 to perceive JA-Ile[39]. JAZs are then ubiquitinated and targeted to the 26S proteasome for degradation. This will release the TFs, which will interact with other partners to activate JA-Ile responsive genes.

To identify homologous sequences to JA signalling components in the three hornwort genomes, we first compiled JA signalling protein sequences from the non-redundant protein database of NCBI, using *A. thaliana* and *M. polymorpha* protein sequences as queries in a BLASTp search. We then used this database to look for homologs of the hornwort proteomes with BLASTp. The top hornwort hits were then blasted against the *M. polymorpha* and *A. thaliana* proteomes at the marchantia.info database to correctly identify the hornwort homologs. Protein sequences from different plant species were aligned with MUSCLE[40] to analyze domain conservation. Trees corresponding to the orthogroups were visualized with the iTOL platform v4.4.2[41].

In line with previous results from the liverwort, *M. polymorpha*, we found that components of the JA signaling pathway are present in all three hornwort genomes[12]. The three hornwort genomes showed conserved sequences for all the jasmonate signalling components found in land plants: COI, JAZ, MYC, NINJA and TPL (Supplementary Figure 10; Supplementary Table 10). It was shown that *A. agrestis* does not synthesize Jasmonic acid but it can likely produce the JA-precursor OPDA (12-oxo-phytodienoic acid) which will cause growth inhibition in *A. agrestis*, *M. polymorpha*, *P. patens* and also in *A. thaliana*[42]. Similar to other bryophytes, none of the *Anthoceros* COI homologs showed an alanine in the equivalent position to AtCOI1^{A384}, which indicates that *Anthoceros* COI do not perceive JA-Ile, the hormone from vascular plants. This is consistent with the previously reported insensitivity of *A. agrestis* Oxford to JA-Ile but not to OPDA[42], which suggests that the bioactive jasmonate perceived by COI in *Anthoceros* might be dn-OPDA as in *Marchantia*. Importantly, most components are represented by a single gene in the three hornwort genomes but JAZ and COI in *A. punctatus*, a situation similar to the *M. polymorpha* genome (Supplementary Table 10). This suggests that function of the JA pathway is likely to be conserved across bryophytes and, to some extent, across land plants as proposed by the recent study on *M. polymorpha*[43].

Strigolactones

Strigolactones are signals secreted into the environment inhibiting branching and promoting mycorrhizal symbiosis. A recent report has suggested an ancient origin for strigolactone synthesis, but a much more recent origin for canonical strigolactone signalling in angiosperms, through duplication and divergence in components of the ancient KAI2-

SCF^{MAX2}-SMXL pathway[44]. KAI2 α/β hydrolase receptors and SMXL proteolytic target proteins have already been shown to exist in hornwort transcriptomes, consistent with the apparent ancient nature of this pathway, and were also identified in *A. agrestis* and *A. punctatus*[44,45]. BLAST searches also revealed unambiguous orthologues of the MAX2 F-box protein in *A. agrestis* and *A. punctatus* (Supplementary Table 10). All other land plant groups have a second clade of KAI2-like proteins, which have ultimately given rise to DWARF14 strigolactone receptors in angiosperms[45]. However, BLAST searches with *A. thaliana KAI2* and *D14*, or *M. polymorpha KAI2A* or *KAI2B* failed to detect any other *KAI2* homologue in either *A. agrestis* or *A. punctatus*, consistent with a previous report using hornwort transcriptomes[45]. Thus, unlike all other land plant groups, hornworts only have a single clade of KAI2/D14-family proteins. These data support the idea that strigolactone signalling is not present in non-seed plants. However, BLAST searches using the *A. thaliana* sequences identified unambiguous homologues of the strigolactone synthesis enzymes CCD7, CCD8 and MAX1 in both *A. agrestis* and *A. punctatus* (Supplementary Table 10)**.** We identified homologues of the *DWARF27* (*D27*) isomerase in both species, but phylogenetic analysis showed that these genes fell into the closely related *DWARF27-LIKE1* (D27-L1) clade and not DWARF27 itself; hornworts therefore appear to lack true DWARF27 orthologues. This suggests that hornworts may have lost *D27*, or that the duplication in the *proto-D27* lineage that gave rise to *D27* and *D27L1* occurred after the divergence of hornworts and other land plants. Nevertheless, hornworts clearly possess the core enzymes for strigolactone synthesis, consistent with the idea that strigolactones are an ancient non-hormonal signal that originated for rhizospheric communication with mycorrhizal fungi[44].

Gibberellic acid (GA) signaling and synthesis

Of the GA signaling related genes we found proper *DELLA* orthologs in *A. agrestis* (Bonn) and *A. punctatus*. There was no proper *DELLA* ortholog in the *A. agrestis* (Oxford) genome which appears to be due to an assembly error because we were able to clone this gene from the very same plant tissue (Supplementary Table 10). The three *Anthoceros* genomes investigated lack proper orthologs of GID1 (GA receptor) and GID2/SLY (specific F-box protein). To demonstrate this, we have retrieved all genes similar to *GID1* and *GID2* and made phylogenetic trees with known orthologs from *Arabidopsis*, *Oryza* and other non-vascular land plants, and we have confirmed that the *Anthoceros* genes are homologs of other *GID1-like* and *GID2-like* genes, but not of the ones involved in GA signalling (Supplementary Figure 11). Overall, this coincides with the findings reported earlier[46] with one exception: this previous analysis also found a possible *GID1* ortholog in *Phaeoceros* and *Paraphymatoceros*. Since this was based on 1KP partial sequences, it is currently difficult to disentangle whether these and other hornworts have bona-fide *GID1* orthologs that have been lost in *Anthoceros* or all hornworts lack proper *GID1* orthologs.

From a qualitative perspective, the presence/absence of GA synthesis genes in *Anthoceros* is essentially identical to *Marchantia polymorpha*[12]. In other words, CPS/KS-, KO-, and KAO-encoding genes are unambiguously present in *Anthoceros* (Supplementary Table 10)[47]. Both moss genomes *P. patens* and *Sphagnum fallax* lack *KAO* genes[34]. Because genes encoding proper GA oxidases are not present in any of the *Anthoceros* genome sequences, we conclude that gibberellins are not synthesized in *Anthoceros*, at least via the canonical

pathway. A striking observation is the expansion of the *CPS/KS* family in *Anthoceros*, many of these genes appearing as tandem duplications. The total numbers are high (around 30), which is significantly different from angiosperms (usually 1 *CPS* and 1 *KS*), and other non-vascular land plants (3-6). We also found that both bifunctional (CPS/KS) and monofunctional (CPS or KS) proteins are present, as proposed also for *M. polymorpha* and *P. patens*[12,34].

Salicylic acid

To identify whether the genome of *A. agrestis* and *A. punctatus* encodes the components for salicylic acid (SA) biosynthesis and signaling, the protein sequences of factors involved in SA biosynthesis (ICS, PAL, PBS3, and EDS5) and its signaling (NPR, TGA, WRKY, and PR1) from *A. thaliana* were used as a reference point[48,49]. The sequences were downloaded from TAIR (Araport11 assembly)[50,51]. Additional protein data were downloaded for the genomes of *P. abies* v.1.0[52], *A. filiculoides* v.1.0, *S. cucullata* v1.2[53], *Selaginella moellendorffii* v.1.0[54], *P. patens* v.3.3[55], *M. polymorpha* v.3.1[12], *C. braunii* v.1.0[56] and *K. nitens* v.1.1[57] from NCBI and Phytozome[58].

An initial candidate set of SA biosynthesis and signaling components from the hornworts and the other streptophyte lineages was acquired using BLASTp[9]. Here, the sequences from *A. thaliana* were used as query against the protein files of *P. abies, A. filiculoides, S. cucullata, S. moellendorffii, P. patens, M. polymorpha, A. agrestis* Bonn, *A. agrestis* Oxford, *A. punctatus, C. braunii* and *K. nitens.* The e-value cutoff was 10^{-5} (Supplementary Table 10).

Initial alignments were produced using MAFFT v.7.427[59] with either a local (L-INS-I) or global (G-INS-I) alignment strategy. G-INS-I-based alignments were chosen for ICS, NPR and TGA, while L-INS-I-based alignments were chosen for EDS5/EDS5H, PR1, PAL, PBS3 and WRKYs. If N- and/or C-terminal regions were too divergent alignments were trimmed to only include the conserved regions (ICS and TGA). Highly divergent sequences were removed from the alignment (Supplementary Figure 12). For PBS3 and PR1 only sequences containing the same domains as the respective reference sequence from *Arabidopsis* were included in the phylogeny to further reduce sequence divergence across alignments (domains for PBS3: GH3, GH3 superfamily, PLN02247, PLN02249 and PLN02620; domains for PR1: CAP_PR-1). All domain searches have been performed using CD batch search[60]. An e-value cutoff of 10^{-5} was used. Phylogenies were built using IQ-TREE v.1.5.5[61] with pre-selection for the best model[62]. The Maximum-likelihood phylogenies are based on 500 bootstrap replicates.

Biosynthesis of salicylic acid. Salicylic acid (SA) is synthesized in the chloroplast and cytoplasm (Supplementary figure 12) from chorismate[63,64]. In the angiosperm *Arabidopsis thaliana,* the first step is catalyzed by Isochorismate synthase 1 (ICS1) and likely the functionally redundant ICS2[65,66]. The product of this reaction is isochorismate, which is exported into the cytoplasm by the MATE-transporter ENHANCED DISEASE SUSCEPTIBILITY 5 (EDS5)[65,66]. Conversion to SA in the cytoplasm requires the acyl acid amido synthetase GH3.12 or AvrPphB SUSCEPTIBLE 3 (PBS3), which uses isochorismate as substrate to produce isochorismate-9-glutamate[66,67]. Formation of SA occurs

spontaneously from isochorismate-9-glutamate[66,67], but the reaction can be enhanced by the Brassicaceae-specific BAHD acyltransferase ENHANCED PSEUDOMONAS SUSCEPTIBILTY 1 (EPS1)[67]. Alternatively, SA can be generated in the chloroplast via a largely unexplored pathway involving Phenylalanine Ammonia Lyase (PAL)[68–70].

We searched the genomes of 11 streptophytes for SA biosynthesis genes via an initial BLASTp search, followed by in-depth phylogenetic analyses (Supplementary Figure 12). We retrieved orthologs of ICS1 and 2 in most streptophyte genomes (Supplementary Figure 12). *Picea abies* appears to have lost both ICS copies. *Azolla filiculoides* and *A. punctatus* each have lost one of the functionally redundant ICS-encoding genes. A direct co-ortholog for PAL1, 2, 3 and 4 was only found in *P. abies* (Supplementary Figure 12). However, based on phylogenetic analyses including both PALs and the related Histidine Ammonia Lyases (HALs) from more eukaryotic supergroups and bacteria, it was shown that all embryophyte lineages as well as the streptophyte algae *K. nitens* possess putative PAL-like candidates[71]. A domain search could confirm the presence of PAL domains (PLN02457) are present in all embryophyte sequences included in our phylogeny (Supplementary Table 24). The PAL-like sequences of streptophyte algae are truncated compared to the embryophyte versions[71]. Therefore, albeit the *K. nitens* has been identified as putative PAL[71], domain analyses can only identify the truncated HAL domain. PBS3 belongs to the GH3 family and clusters with other GH3 family members of *A. thaliana,* suggesting it arose during an expansion within *A. thaliana* (Supplementary Figure 12). No *Anthoceros* sequences clustered with this exact subfamily, albeit several sequences were found that had domains occurring in the proteins belonging to the GH3 family (i.e. GH3, GH3 superfamily, PLN02247, PLN02249 and PLN02620). The transporter *At*EDS5 is duplicated within *A. thaliana,* the duplication called *At*EDS5 Homolog (*At*EDS5H). The role of *At*EDS5H in SA signaling is currently unknown[72]. In our phylogenetic analyses *At*EDS5H and *At*EDS5 cluster together, suggesting a recent duplication in *A. thaliana* (Supplementary Figure 12). Therefore, it is hard to hypothesize the ancestral function in the absence of further functional studies. Our current analyses can only go so far as to show that homologs of EDS5/EDS5H exist in most embryophyte lineages (except *P. abies*), including both *Anthoceros* species. The presence of the entire biosynthesis pathway in the embryophytes (Supplementary Figure 12), however, implies that some kind of SA transfer mechanism of SA from chloroplast to cytoplasm must exist.

Perception and downstream signaling of salicylic acid. In the cytoplasm, SA is perceived by three receptors in the angiosperm *A. thaliana*: NONEXPRESSER OF PR GENES (NPR)1, 3 and 4[73,74]. The NPR receptors further activate downstream signaling by directly or indirectly interacting with TGACG-binding bZIP (TGA) and WRKY transcription factors (TFs) (Supplementary Figure 12)[29,75,76]. It has recently been shown that NPR1 is a positive regulator of SA signaling, while NPR3 and 4 are negative regulators[77,78]. Downstream the TGA and WRKY TFs activate SA-specific signaling and may also interfere with jasmonic acid signaling, thereby establishing the antagonistic relationship of these two phytohormones that is observed in *A. thaliana*. Yet, it should be noted that the antagonistic relationship between JA and SA may not exist in all plant lineages to the extent it is observed in *A. thaliana*[79–82].

We investigated the presence of SA signaling components in *Anthoceros* sp. and other streptophyte lineages and confirmed that NPR1, 3 and 4 have originated through recent duplication within *A. thaliana* (Supplementary Figure 12). Only the gymnosperm *P. abies* had

a direct homolog of the NPR clade with high support, which warrants attention because it lacked both ICS as well as SA exporter candidates (Supplementary Figure 12). NPR-like sequences were further found in *Salvinia cucullata, A. filiculoides, S. moellendorffii* and the bryophytes *P. patens* and *M. polymorpha,* but with low bootstrap support (support >50 and <70; Supplementary Figure 12). *A. agrestis, A. punctatus* and the two streptophyte algae, *C. braunii* and *K. nitens,* possess the related and also BTB/POZ domain-containing proteins BLADE ON PETIOLE 1 and 2 (BOP1 and 2), involved in plant development[83], but lack homologs of NPRs (Supplementary Figure 12).

Our TGA analyses included the closely related bZIPs, bZIP28 and bZIP49, belonging to bZIP group B[84] to better distinguish which TFs are TGA (bZIP group D) and which are other bZIP factors. Yet, bZIP group B clustered within the TGAs, leading to an unresolved phylogeny (Supplementary Figure 12). Such results are generally not surprising for evolutionary analyses of TFs. It is noteworthy that, in a previous study, we observed that TGAs show a higher conservation to each other than to any of the other bZIP groups[85]. We therefore used this information to identify the most likely TGA-like sequences. *At*TGA paralogs show a 58.98±11.41% amino acid (aa) identity between each other, while they are only 7.68±0.95% identical to bZIP group B TFs on the aa level. Using this information, we found that most sequences in the supported cluster that includes *A. thaliana's* TGAs as well as bZIP28 and 49 showed an aa identity of over 47.5% to at least one TGA family member (Supplementary Figure 12; Supplementary Table 10). On the contrary, sequences outside of this cluster showed an aa identity <29% to each of the TGA members. Only two sequences, from within the TGA-like/bZIP group B cluster diverted from this pattern: One is a protein sequence from *P. patens* (Pp3c11_3960V3.1.p), which however clustered with high bootstrap support (bootstrap value 98) with bZIP28 and 49, suggesting that it is more likely a group B bZIP. The second is from *A. fliculoides* (Azfi_s0113.g045990), which clusters with other TGA-like sequences from *S. cucullata* and *A. filiculoides.* It is 41.10 to 47.16% identical to *At*TGA1 to 10. These values are very close to the sequence identity of TGAs within *A. thaliana* and largely different to the <29% aa identity cutoff for sequences outside the TGA-like cluster, suggesting that Azfi_s0113.g045990 may also be a TGA homolog (Supplementary Figure 12).

We next investigated the evolutionary conservation of WRKY TFs involved in SA signaling. To do so, we selected six WRKYs, for which function in SA signaling or the JA/SA antagonism was reported in *A. thaliana*[53,86–89]: WRKY22, 41, 50, 51, 62 and 70. Here we found homologs to the clade of WRKY41 and 70 in several land plants (Supplementary Figure 12) with high bootstrap support (bootstrap value 72). This included one homolog in each *A. agrestis* isolates and one in *A. punctatus*. Downstream these TFs activate a characteristic SA-specific and dependent gene expression. One of the best characterized marker genes for SA-specific gene expression in *A. thaliana* is Pathogenesis-related 1 (*PR1*). Phylogenetic analyses of PR1 remained inconclusive, due little or no bootstrap support. Only for *P. abies* weakly supported (bootstrap value 52) PR1-like sequences were identified (Supplementary Figure 12). It is however noteworthy that all sequences included in our phylogenetic analyses possessed a CAP_PR-1 domain (Supplementary Table 10), distinguishing them from proteins which only encode CAP superfamily domains. They are therefore belonging to the PR1 protein subfamily within the CAP superfamily.

Our evolutionary analyses suggest that the genetic chassis for SA biosynthesis occurs across the streptophytes. Yet, for example *P. abies* may synthesize SA singularly through the PAL route, given the absence of proteins with any similarity to ICS. Further, our analyses reveal a lack of putative SA exporters in both the gymnosperm *P. abies,* as well as the two streptophyte algae *C. braunii* and *K. nitens.* Given that SA was measured in the latter[57], it may be that other mechanisms than EDS5-dependent export from chloroplast to cytoplasm exist in some green lineages. Concerning the here analyzed hornworts the genetic potential for SA biosynthesis and export is fully present in their genomes. We further found that, like the other analyzed streptophyte genomes, those of the three hornworts (*A. agrestis* Bonn, *A. agrestis* Oxford and *A. punctatus*) encode the components to induce downstream SA signaling (TGA-like and WRKY TFs), yet it is unclear how SA might be perceived in hornworts, given that no NPR-like sequences were retrieved.

Ethylene and cytokinin signaling/synthesis

Ethylene and cytokinin signaling, both two-component systems, has evolved in the Charophytes and is not present in any of the Chlorophytes[12,29,56]. We used *A. thaliana*, *M. polymorpha* and *P. patens* genes related to ethylene and cytokinin signaling and synthesis and BLASTp searches to find their corresponding homologs in the three hornwort genomes. The ethylene and cytokinin signaling and synthesis related gene set of the three hornwort genomes was very similar that was reported earlier for *M. polymorpha* and *P. patens*[12,29,34]. In brief, the full set of cytokinin signaling and synthesis genes are present in all three hornwort genomes further confirming the conservation of this pathway in all land plants (Supplementary Table 10). Genes involved in ethylene signaling and synthesis are also present, but as was shown for other non-seed plants[12,34,53] a gene coding for a distinct ethylene-forming enzyme is missing (ACO)(Supplementary Table 10). Biological function of ethylene and cytokinins are relatively well investigated in *P. patens* and *M. polymorpha*[90–92]. In contrast, effect and biological significance of cytokinins and ethylene is poorly known in hornworts and needs to be explored in the future.

Genes for plastidial peptidoglycan biosynthesis

Plastids originated from cyanobacteria through endosymbiosis. Cyanobacteria are gram negative bacteria and are hence encased by a murein layer-based cell wall. The plastid number and morphology of streptophyte algae, mosses, lycophytes, and—to a certain degree—ferns are affected by inhibitors (β-lactam antibiotics) of peptidoglycan (PG) biosynthesis[93–96]. Various non-seed plants bear a genetic chassis that is homologous to PG biosynthesis of bacteria, including a set of *mur* genes[97,98]. *P. patens* that has had these PG biosynthesis genes knocked out bear alterations in plastid number and morphology[97,99]—corroborating the aforementioned inhibitor-based data. These insights culminated in a study by Hirano et al.[98], who used a specific D-Ala-D-Ala labeling technique to visualize the PG layer of the model moss *P. patens*. The genes for PG biosynthesis are present in the genome of *M. polymorpha* as well[12,98] and they are affected by PG-inhibiting antibiotics[100]. In

sum, among land plants, mosses, liverworts, and lycophytes have genes for PG biosynthesis. What about hornworts?

Genomes of *P. patens*[55] and *M. polymorpha*[12] have genes for PG biosynthesis. To screen the three *Anthoceros* genomes for the presence of PG biosynthesis genes, we downloaded the protein sequences of the genes *murA-G* and *mraY* (annotated based on KEGG) of *Nostoc punctiforme* PCC 73102[101] as well as the *P. patens* proteins DDL, PBP, and DAC[97]. We used a reciprocal BLASTp screening to identify putative orthologs of these genes in all three assemblies of *Anthoceros*. All identified *Anthoceros* proteins were investigated for the relevant domains via Interproscan. Both *Anthoceros agrestis* and *Anthoceros punctatus* had genes likely coding for MURA to MURG, MRAY, DDL, PBP, and DAC in their genomes (Supplementary Figure 17, Supplementary Table 10). They hence have the full homologous genetic chassis for PG biosynthesis. Altogether, our data indicate that all hornworts have a similar chassis for PG biosynthesis as mosses. This means that PG biosynthesis was likely present in the last common ancestor of all land plants and was lost along the trajectory of euphyllophyte evolution.

Monoplastidy

Recently, Grosche and Rensing[102] reported an intriguing co-occurrence of genes for FtsZ3 in those photosynthetic eukaryotes whose plastids bear PG layers. As in their cyanobacterial progenitors, plastidial FtsZ proteins assemble into a contractile ring that facilitates the fission of plastids[103]. Knock-out studies of the five *ftsZ* genes found in *P. patens* revealed that loss of function in FtsZ results in alterations of plastid number and morphology[104]. Along the trajectory of streptophyte evolution, having more than one plastid per cell is a trait that was likely gained within the Phragmoplastophyta[105]. This might have occurred via a biplastidy in the closest algal relatives of land plants[105]—and hence potentially the earliest land plants.

Hornwort data are essential for assembling the picture of how the last common ancestor of all land plants—and hence also the earliest land plants—might have looked like. Further, hornworts might represent an interesting case for the evolution of polyplastidy: some of them can be considered monoplastidic or biplastidic[105,106]. Since the PG layer and FtsZ3 are proposed to be functionally interconnected[102,105], we analyzed the FtsZ repertoire encoded in the *Anthoceros* genomes (Supplementary Table 10). In all three genomes of *Anthoceros* we detected only two *ftsZ* genes each. Based on the data of Grosche and Rensing[102], we constructed a phylogeny (G-INS-I alignment; ML; LG+I+G4; 100 bootstrap replicates; IQ-TREE v1.5.5) that included all putative *Anthoceros* FtsZ proteins as well as the FtsZ protein candidates that we found in the genomes of the ferns *A. filiculoides* and *S. cucullata* (Supplementary Figure 18). Interestingly, both species of *Anthoceros* had only an ortholog of FtsZ1 and FtsZ3, but not FtsZ2. The same was true for the genome of *Chara braunii*[56], but not for *Klebsormidium nitens*[57]. This raises the question of whether FtsZ2 was present in the last common ancestor of land plants and Klebsormidiophyceae and then (a) simply lost in *Chara* and *Anthoceros* or (b) whether it speaks to independent origins of FtsZ2. Altogether, the evolution of FtsZ2, the co-occurrence of genes coding for FtsZ3 and PG in *Anthoceros* corroborates the previously observed patterns for mosses and liverworts[102].

To further understand the monoplastidy in hornworts, we investigated if there is any other gene involved in controlling plastid division and number be absent in the *Anthoceros* genomes. *A. thaliana* (TAIR10 genome with Araport11 annotations), *P. patens* (v3.3 from Phytozome) and *M. polymorpha* (v3.1 Phytozome) homologs of the key genes known to control chloroplast division (see Supplementary Table 10)[107–109] were queried to each of the three *Anthoceros* proteomes or genomes using BLASTp and tBLASTn[9] respectively, with a threshold e-value of $1e^{-5}$. We found all of them are present in the *Anthoceros* genomes (Supplementary Table 10). Control of their expression or their difference in the amino acid sequence may have determined the monoplastidy in hornworts.

Plastid targeted proteins

In order to further our understanding of hornwort plastid biology, we compared the sets of proteins with a supposed function in the plastid between the three hornworts and two mosses (*P. patens*, *S. fallax*). We hypothesized that the reduced plastid number (solitary plastids in the three investigated hornwort species) will have an effect on the plastid targeted gene set. To this end, we subjected all predicted proteins, including isoforms, per species to a targetP analysis[110,111] to identify transit peptides and predict the cellular localization of all proteins. The analysis was carried out under default settings, allowing for 2,000 peptides to be searched at once; the prediction of cleavage sites was enabled. All proteins with a chloroplast- or thylakoid lumen-specific transit peptide were extracted from the data sets. Subsequently, an all-versus-all BLASTp search of plastid-predicted proteins (BLOSUM62 matrix, gap and extension penalty: 11 and 1, respectively; word size: 6, e-value: 10E-3; no low complexity filter) was used to identify proteins that were conserved in all species. Proteins missing an unambiguous transit peptide in one species were searched for again in the protein pool using BLASTp (as above). If that failed, too, a protein-to-genome search was performed using exonerate[112] with an allowed minimum and maximum intron size of 20 and 8,000 bp, respectively. Together, this approach discovered over 3,000 plastid-targeted genes that are common in hornworts and mosses (Supplementary Table 18). Six hornwort proteins with plastid-specific transit peptide had no match in *P. patens* or *S. fallax*. Of these, one showed some similarity to the bacterial ribosomal protein L34 (54% over a length of 39 aa), and all others contained short amino acid sequences (max. 40 aa in length) that resembled peptides from bacterial proteins of unknown function. Our analysis suggests some difference between the plastid targeted set of proteins in hornworts and mosses. Nevertheless, the biological significance of these differences needs to be further investigated by functional essays.

Photoreceptors

Being able to accurately sense and respond to light cues is fundamental for plants to adapt to the terrestrial environment[113]. Previously, a chimeric photoreceptor, neochrome, was found in hornworts that consist of part phytochrome (a red/far-red-light receptor) and part phototropin (a blue sensor)[114]. Phylogenetic analyses showed that hornwort neochrome was

horizontally transferred to ferns, which likely enabled ferns to survive and diversify in the angiosperm-dominated forests[114]. With the newly assembled genomes, we characterized the complete photoreceptor repertoire in hornworts (Supplementary Table 10). We found that with the exception of cryptochrome, all the photoreceptors are single-copy genes in the *Anthoceros* genomes. Such a reduced number of photoreceptor orthologs is highly unusual for a plant genome[115,116], but similar to that in *M. polymorpha*[12]. Most of the key light signaling components, including phytochrome-interacting factors (PIFs), are single-copy as well, a situation again found in *M. polymorpha* but not in other plant lineages (Supplementary Table 10). Future work investigating the presumably simpler light signalling networks in hornworts and *M. polymorpha* should form the basis for understanding the diversification of light regulatory modules in vascular plants.

Literature cited

1. Heslop-Harrison, J. S. Comparative genome organization in plants: from sequence and markers to chromatin and chromosomes. *Plant Cell* **12**, 617–636 (2000).
2. Zhu, W. *et al.* Altered chromatin compaction and histone methylation drive non-additive gene expression in an interspecific Arabidopsis hybrid. *Genome Biol.* **18**, 157 (2017).
3. Melters, D. P. *et al.* Comparative analysis of tandem repeats from hundreds of species reveals unique insights into centromere evolution. *Genome Biol.* **14**, R10 (2013).
4. Richards, E. J. & Ausubel, F. M. Isolation of a higher eukaryotic telomere from Arabidopsis thaliana. *Cell* **53**, 127–136 (1988).
5. Fulnečková, J. *et al.* Dynamic evolution of telomeric sequences in the green algal order Chlamydomonadales. *Genome Biol. Evol.* **4**, 248–264 (2012).
6. Sykorova, E. *et al.* The absence of Arabidopsis-type telomeres in Cestrum and closely related genera Vestia and Sessea (Solanaceae): first evidence from eudicots. *Plant J.* **34**, 283–291 (2003).
7. Fajkus, P. *et al.* Allium telomeres unmasked: the unusual telomeric sequence (CTCGGTTATGGG)n is synthesized by telomerase. *Plant J.* **85**, 337–347 (2016).
8. Lang, D. *et al.* The Physcomitrella patens chromosome-scale assembly reveals moss genome structure and evolution. *Plant J.* **93**, 515–533 (2018).
9. Altschul, S. F., Gish, W., Miller, W., Myers, E. W. & Lipman, D. J. Basic local alignment

search tool. *J. Mol. Biol.* **215**, 403–410 (1990).

10. Marçais, G. *et al.* MUMmer4: A fast and versatile genome alignment system. *PLoS Comput. Biol.* **14**, e1005944 (2018).
11. Kent, W. J. BLAT—The BLAST-Like Alignment Tool. *Genome Res.* **12**, 656–664 (2002).
12. Bowman, J. L. *et al.* Insights into Land Plant Evolution Garnered from the Marchantia polymorpha Genome. *Cell* **171**, 287–304.e15 (2017).
13. Evkaikina, A. I. *et al.* The Huperzia selago shoot tip transcriptome sheds new light on the evolution of leaves. *Genome Biol. Evol.* **9**, 2444–2460 (2017).
14. Finet, C. *et al.* Evolution of the YABBY gene family in seed plants. *Evol. Dev.* **18**, 116–126 (2016).
15. Somssich, M., Je, B. I., Simon, R. & Jackson, D. CLAVATA-WUSCHEL signaling in the shoot meristem. *Development* **143**, 3238–3248 (2016).
16. Whitewoods, C. D. *et al.* CLAVATA was a genetic novelty for the morphological innovation of 3D growth in land plants. *Curr. Biol.* **28**, 2365–2376 (2018).
17. Hirakawa, Y. *et al.* Control of proliferation in the haploid meristem by CLE peptide signaling in Marchantia polymorpha. *PLoS Genet.* **15**, e1007997 (2019).
18. Tanahashi, T., Sumikawa, N., Kato, M. & Hasebe, M. Diversification of gene function: homologs of the floral regulator FLO/LFY control the first zygotic cell division in the moss Physcomitrella patens. *Development* **132**, 1727–1736 (2005).
19. Sakakibara, K. *et al.* WOX13-like genes are required for reprogramming of leaf and protoplast cells into stem cells in the moss Physcomitrella patens. *Development* **141**, 1660–1670 (2014).
20. Lee, Z. H., Hirakawa, T., Yamaguchi, N. & Ito, T. The roles of plant hormones and their interactions with regulatory genes in determining meristem activity. *Int. J. Mol. Sci.* **20**, 4065 (2019).
21. Denis, E. *et al.* WOX14 promotes bioactive gibberellin synthesis and vascular cell differentiation in Arabidopsis. *Plant J.* **90**, 560–572 (2017).
22. Romera-Branchat, M., Ripoll, J. J., Yanofsky, M. F. & Pelaz, S. The WOX 13 homeobox

gene promotes replum formation in the Arabidopsis thaliana fruit. *Plant J.* **73**, 37–49 (2013).

23. Sayou, C. *et al.* A promiscuous intermediate underlies the evolution of LEAFY DNA binding specificity. *Science* **343**, 645–648 (2014).
24. Peterson, K. M., Rychel, A. L. & Torii, K. U. Out of the mouths of plants: the molecular basis of the evolution and diversity of stomatal development. *Plant Cell* **22**, 296–306 (2010).
25. Lee, L. R. & Bergmann, D. C. The plant stomatal lineage at a glance. *J. Cell Sci.* **132**, jcs228551 (2019).
26. Chater, C. C. C., Caine, R. S., Fleming, A. J. & Gray, J. E. Origins and Evolution of Stomatal Development. *Plant Physiol* **174**, 624–638 (2017).
27. Duckett, J. G. & Pressel, S. The evolution of the stomatal apparatus: intercellular spaces and sporophyte water relations in bryophytes—two ignored dimensions. *Philos. Trans. R. Soc. Lond. B Biol. Sci.* **373**, 20160498 (2018).
28. Chater, C. C. *et al.* Origin and function of stomata in the moss Physcomitrella patens. *Nat Plants* **2**, 16179 (2016).
29. Wang, C., Liu, Y., Li, S.-S. & Han, G.-Z. Insights into the origin and evolution of the plant hormone signaling machinery. *Plant Physiol.* **167**, 872–886 (2015).
30. Cutler, S. R., Rodriguez, P. L., Finkelstein, R. R. & Abrams, S. R. Abscisic acid: emergence of a core signaling network. *Annu. Rev. Plant Biol.* **61**, 651–679 (2010).
31. Stevenson, S. R. *et al.* Genetic analysis of Physcomitrella patens identifies ABSCISIC ACID NON-RESPONSIVE (ANR), a regulator of ABA responses unique to basal land plants and required for desiccation tolerance. *Plant Cell* **28**, 1310–1327 (2016).
32. Sussmilch, F. C., Atallah, N. M., Brodribb, T. J., Banks, J. A. & McAdam, S. A. M. Abscisic acid (ABA) and key proteins in its perception and signaling pathways are ancient, but their roles have changed through time. *Plant Signal. Behav.* **12**, e1365210 (2017).
33. Artur, M. A. S., Zhao, T., Ligterink, W., Schranz, E. & Hilhorst, H. W. M. Dissecting the

genomic diversification of late embryogenesis abundant (LEA) protein gene families in plants. *Genome Biol. Evol.* **11**, 459–471 (2019).

34. Rensing, S. A. *et al.* The Physcomitrella genome reveals evolutionary insights into the conquest of land by plants. *Science* **319**, 64–69 (2008).
35. Jones, P. *et al.* InterProScan 5: genome-scale protein function classification. *Bioinformatics* **30**, 1236–1240 (2014).
36. Eklund, D. M. *et al.* Auxin Produced by the Indole-3-Pyruvic Acid Pathway Regulates Development and Gemmae Dormancy in the Liverwort Marchantia polymorpha. *Plant Cell* **27**, 1650–1669 (2015).
37. Goossens, J., Fernández-Calvo, P., Schweizer, F. & Goossens, A. Jasmonates: signal transduction components and their roles in environmental stress responses. *Plant Mol. Biol.* **91**, 673–689 (2016).
38. Ruan, J. *et al.* Jasmonic Acid Signaling Pathway in Plants. *Int. J. Mol. Sci.* **20**, (2019).
39. Sheard, L. B. *et al.* Jasmonate perception by inositol-phosphate-potentiated COI1-JAZ co-receptor. *Nature* **468**, 400–405 (2010).
40. Edgar, R. C. MUSCLE: multiple sequence alignment with high accuracy and high throughput. *Nucleic Acids Res.* **32**, 1792–1797 (2004).
41. Letunic, I. & Bork, P. Interactive Tree Of Life (iTOL) v4: recent updates and new developments. *Nucleic Acids Res.* **47**, W256–W259 (2019).
42. Monte, I. *et al.* Ligand-receptor co-evolution shaped the jasmonate pathway in land plants. *Nat. Chem. Biol.* **14**, 480–488 (2018).
43. Monte, I. *et al.* A single JAZ repressor controls the jasmonate pathway in Marchantia polymorpha. *Mol. Plant* **12**, 185–198 (2019).
44. Walker, C. H., Siu-Ting, K., Taylor, A., O'Connell, M. J. & Bennett, T. Strigolactone synthesis is ancestral in land plants, but canonical strigolactone signalling is a flowering plant innovation. *BMC Biol.* **17**, 70 (2019).
45. Bythell-Douglas, R. *et al.* Evolution of strigolactone receptors by gradual neo-functionalization of KAI2 paralogues. *BMC Biol.* **15**, (2017).

46. Hernandez-Garcia, J., Briones-Moreno, A., Dumas, R. & Blazquez, M. A. Origin of gibberellin-dependent transcriptional regulation by molecular exploitation of a transactivation domain in DELLA proteins. *Mol. Biol. Evol.* **36**, 908–918 (2019).

47. Salazar-Cerezo, S., Martínez-Montiel, N., García-Sánchez, J., Pérez-Y-Terrón, R. & Martínez-Contreras, R. D. Gibberellin biosynthesis and metabolism: A convergent route for plants, fungi and bacteria. *Microbiol. Res.* **208**, 85–98 (2018).

48. Maruri-López, I., Aviles-Baltazar, N. Y., Buchala, A. & Serrano, M. Intra and Extracellular Journey of the Phytohormone Salicylic Acid. *Front. Plant Sci.* **10**, 423 (2019).

49. Zhang, Y. & Li, X. Salicylic acid: biosynthesis, perception, and contributions to plant immunity. *Curr. Opin. Plant Biol.* **50**, 29–36 (2019).

50. Huala, E. *et al.* The Arabidopsis Information Resource (TAIR): a comprehensive database and web-based information retrieval, analysis, and visualization system for a model plant. *Nucleic Acids Res.* **29**, 102–105 (2001).

51. Berardini, T. Z. *et al.* The Arabidopsis information resource: making and mining the 'gold standard' annotated reference plant genome. *Genesis* **53**, 474–485 (2015).

52. Nystedt, B. *et al.* The Norway spruce genome sequence and conifer genome evolution. *Nature* **497**, 579–584 (2013).

53. Li, F.-W. *et al.* Fern genomes elucidate land plant evolution and cyanobacterial symbioses. *Nat Plants* **4**, 460–472 (2018).

54. Banks, J. A. *et al.* The Selaginella genome identifies genetic changes associated with the evolution of vascular plants. *Science* **332**, 960–963 (2011).

55. Lang, D., Ullrich, K. K., Murat, F. & Fuchs, J. The Physcomitrella patens chromosome-scale assembly reveals moss genome structure and evolution. *Plant J* **93**, 515–533 (2018).

56. Nishiyama, T. *et al.* The Chara Genome: Secondary Complexity and Implications for Plant Terrestrialization. *Cell* **174**, 448–464.e24 (2018).

57. Hori, K. *et al.* Klebsormidium flaccidum genome reveals primary factors for plant

terrestrial adaptation. *Nat. Commun.* **5**, 3978 (2014).

58. Goodstein, D. M. *et al.* Phytozome: a comparative platform for green plant genomics. *Nucleic Acids Res.* **40**, D1178–86 (2012).
59. Katoh, K. MAFFT: a novel method for rapid multiple sequence alignment based on fast Fourier transform. *Nucleic Acids Res.* **30**, 3059–3066 (2002).
60. Marchler-Bauer, A. & Bryant, S. H. CD-Search: protein domain annotations on the fly. *Nucleic Acids Res.* **32**, W327–W331 (2004).
61. Nguyen, L.-T., Schmidt, H. A., von Haeseler, A. & Minh, B. Q. IQ-TREE: a fast and effective stochastic algorithm for estimating maximum-likelihood phylogenies. *Mol. Biol. Evol.* **32**, 268–274 (2015).
62. Kalyaanamoorthy, S., Minh, B. Q., Wong, T. K. F., von Haeseler, A. & Jermiin, L. S. ModelFinder: fast model selection for accurate phylogenetic estimates. *Nat. Methods* **14**, 587–589 (2017).
63. Wildermuth, M. C., Dewdney, J., Wu, G. & Ausubel, F. M. Isochorismate synthase is required to synthesize salicylic acid for plant defence. *Nature* **414**, 562–565 (2001).
64. Garcion, C. *et al.* Characterization and biological function of the ISOCHORISMATE SYNTHASE2 gene of Arabidopsis. *Plant Physiol.* **147**, 1279–1287 (2008).
65. Serrano, M. *et al.* Export of salicylic acid from the chloroplast requires the multidrug and toxin extrusion-like transporter EDS5. *Plant Physiol.* **162**, 1815–1821 (2013).
66. Rekhter, D. *et al.* Isochorismate-derived biosynthesis of the plant stress hormone salicylic acid. *Science* **365**, 498–502 (2019).
67. Torrens-Spence, M. P. *et al.* PBS3 and EPS1 complete salicylic acid biosynthesis from isochorismate in Arabidopsis. *Biorxiv* 601948 (2019).
68. Meuwly, P., Molders, W., Buchala, A. & Metraux, J. P. Local and Systemic Biosynthesis of Salicylic Acid in Infected Cucumber Plants. *Plant Physiol.* **109**, 1107–1114 (1995).
69. Pallas, J. A., Paiva, N. L., Lamb, C. & Dixon, R. A. Tobacco plants epigenetically suppressed in phenylalanine ammonia-lyase expression do not develop systemic acquired resistance in response to infection by tobacco mosaic virus. *Plant J.* **10**, 281–

293 (1996).

70. Coquoz, J. L., Buchala, A. & Metraux, J. P. The biosynthesis of salicylic acid in potato plants. *Plant Physiol.* **117**, 1095–1101 (1998).

71. de Vries, J., de Vries, S., Slamovits, C. H., Rose, L. E. & Archibald, J. M. How embryophytic is the biosynthesis of phenylpropanoids and their derivatives in streptophyte algae? *Plant Cell Physiol.* **58**, 934–945 (2017).

72. Parinthawong, N., Cottier, S., Buchala, A., Nawrath, C. & Métraux, J.-P. Localization and expression of EDS5H a homologue of the SA transporter EDS5. *BMC Plant Biol.* **15**, 135 (2015).

73. Fu, Z. Q. *et al.* NPR3 and NPR4 are receptors for the immune signal salicylic acid in plants. *Nature* **486**, 228–232 (2012).

74. Wu, Y. *et al.* The Arabidopsis NPR1 protein is a receptor for the plant defense hormone salicylic acid. *Cell Rep.* **1**, 639–647 (2012).

75. Després, C., DeLong, C., Glaze, S., Liu, E. & Fobert, P. R. The Arabidopsis NPR1/NIM1 protein enhances the DNA binding activity of a subgroup of the TGA family of bZIP transcription factors. *Plant Cell* **12**, 279–290 (2000).

76. Zhang, Y., Fan, W., Kinkema, M., Li, X. & Dong, X. Interaction of NPR1 with basic leucine zipper protein transcription factors that bind sequences required for salicylic acid induction of the PR-1 gene. *Proc. Natl. Acad. Sci. U. S. A.* **96**, 6523–6528 (1999).

77. Shi, Z., Maximova, S., Liu, Y., Verica, J. & Guiltinan, M. J. The salicylic acid receptor NPR3 is a negative regulator of the transcriptional defense response during early flower development in Arabidopsis. *Mol. Plant* **6**, 802–816 (2013).

78. Ding, Y. *et al.* Opposite Roles of Salicylic Acid Receptors NPR1 and NPR3/NPR4 in Transcriptional Regulation of Plant Immunity. *Cell* **173**, 1454–1467.e15 (2018).

79. de Vries, S. *et al.* On plant defense signaling networks and early land plant evolution. *Commun. Integr. Biol.* **11**, 1–14 (2018).

80. Xu, M., Dong, J., Wang, H. & Huang, L. Complementary action of jasmonic acid on salicylic acid in mediating fungal elicitor-induced flavonol glycoside accumulation of

Ginkgo biloba cells. *Plant Cell Environ.* **32**, 960–967 (2009).

81. Thaler, J. S., Humphrey, P. T. & Whiteman, N. K. Evolution of jasmonate and salicylate signal crosstalk. *Trends Plant Sci.* **17**, 260–270 (2012).
82. Arnerup, J. *et al.* The primary module in Norway spruce defence signalling against H. annosum s.l. seems to be jasmonate-mediated signalling without antagonism of salicylate-mediated signalling. *Planta* **237**, 1037–1045 (2013).
83. Norberg, M., Holmlund, M. & Nilsson, O. The BLADE ON PETIOLE genes act redundantly to control the growth and development of lateral organs. *Development* **132**, 2203–2213 (2005).
84. Jakoby, M. *et al.* bZIP transcription factors in Arabidopsis. *Trends Plant Sci.* **7**, 106–111 (2002).
85. de Vries, S. *et al.* Jasmonic and salicylic acid response in the fern Azolla filiculoides and its cyanobiont. *Plant Cell Environ.* **41**, 2530–2548 (2018).
86. Gao, Q.-M., Venugopal, S., Navarre, D. & Kachroo, A. Low oleic acid-derived repression of jasmonic acid-inducible defense responses requires the WRKY50 and WRKY51 proteins. *Plant Physiol.* **155**, 464–476 (2011).
87. Mao, P., Duan, M., Wei, C. & Li, Y. WRKY62 transcription factor acts downstream of cytosolic NPR1 and negatively regulates jasmonate-responsive gene expression. *Plant Cell Physiol.* **48**, 833–842 (2007).
88. Higashi, K. *et al.* Modulation of defense signal transduction by flagellin-induced WRKY41 transcription factor in Arabidopsis thaliana. *Mol. Genet. Genomics* **279**, 303–312 (2008).
89. Kloth, K. J. *et al.* AtWRKY22 promotes susceptibility to aphids and modulates salicylic acid and jasmonic acid signalling. *J. Exp. Bot.* **67**, 3383–3396 (2016).
90. von Schwartzenberg, K. Moss biology and phytohormones-cytokinins in Physcomitrella. *Plant Biol.* **8**, 382–388 (2006).
91. Aki, S. S., Nishihama, R., Kohchi, T. & Umeda, M. Cytokinin signaling coordinates development of diverse organs in Marchantia polymorpha. *Plant Signal. Behav.*

1668232 (2019).

92. Aki, S. S. *et al.* Cytokinin signaling Is essential for organ formation in Marchantia polymorpha. *Plant Cell Physiol* **60**, 1842–1854 (2019).

93. Kasten, B. & Reski, R. β-Lactam antibiotics inhibit chloroplast division in a moss (Physcomitrella patens) but not in tomato (Lycopersicon esculentum). *J. Plant Physiol.* **150**, 137–140 (1997).

94. Izumi, Y., Ono, K. & Takano, H. Inhibition of plastid division by ampicillin in the pteridophyte Selaginella nipponica Fr. et Sav. *Plant Cell Physiol.* **44**, 183–189 (2003).

95. Katayama, N. *et al.* Effects of antibiotics that inhibit the bacterial peptidoglycan synthesis pathway on moss chloroplast division. *Plant Cell Physiol.* **44**, 776–781 (2003).

96. Matsumoto, H., Takechi, K., Sato, H., Takio, S. & Takano, H. Treatment with antibiotics that interfere with peptidoglycan biosynthesis inhibits chloroplast division in the desmid Closterium. *PLoS One* **7**, e40734 (2012).

97. Machida, M. *et al.* Genes for the peptidoglycan synthesis pathway are essential for chloroplast division in moss. *Proc. Natl. Acad. Sci. U. S. A.* **103**, 6753–6758 (2006).

98. Hirano, T. *et al.* Moss chloroplasts are surrounded by a peptidoglycan wall containing D-amino acids. *Plant Cell* **28**, 1521–1532 (2016).

99. Homi, S. *et al.* The peptidoglycan biosynthesis genes MurA and MraY are related to chloroplast division in the moss Physcomitrella patens. *Plant Cell Physiol.* **50**, 2047–2056 (2009).

100. Tounou, E., Takio, S., Sakai, A., Ono, K. & Takano, H. Ampicillin inhibits chloroplast division in cultured cells of the liverwort Marchantia polymorpha. *Cytologia* **67**, 429–434 (2002).

101. Meeks, J. C. *et al.* An overview of the genome of Nostoc punctiforme, a multicellular, symbiotic cyanobacterium. *Photosynth. Res.* **70**, 85–106 (2001).

102. Grosche, C. & Rensing, S. A. Three rings for the evolution of plastid shape: a tale of land plant FtsZ. *Protoplasma* **254**, 1879–1885 (2017).

103. Bi, E. F. & Lutkenhaus, J. FtsZ ring structure associated with division in Escherichia coli.

Nature **354**, 161–164 (1991).

104. Martin, A. *et al.* Targeted gene knockouts reveal overlapping functions of the five Physcomitrella patens FtsZ isoforms in chloroplast division, chloroplast shaping, cell patterning, plant development, and gravity sensing. *Mol. Plant* **2**, 1359–1372 (2009).

105. de Vries, J. & Gould, S. B. The monoplastidic bottleneck in algae and plant evolution. *J. Cell Sci.* **131**, (2018).

106. Vaughn, K. C. *et al.* The anthocerote chloroplast: a review. *New Phytol.* **120**, 169–190 (1992).

107. Chen, C., MacCready, J. S., Ducat, D. C. & Osteryoung, K. W. The Molecular Machinery of Chloroplast Division. *Plant Physiol.* **176**, 138–151 (2018).

108. Yang, Y., Glynn, J. M., Olson, B. J., Schmitz, A. J. & Osteryoung, K. W. Plastid division: across time and space. *Curr. Opin. Plant Biol.* **11**, 577–584 (2008).

109. Fulgosi, H., Gerdes, L., Westphal, S., Glockmann, C. & Soll, J. Cell and chloroplast division requires ARTEMIS. *Proc. Natl. Acad. Sci. U. S. A.* **99**, 11501–11506 (2002).

110. Emanuelsson, O., Nielsen, H., Brunak, S. & von Heijne, G. Predicting subcellular localization of proteins based on their N-terminal amino acid sequence. *J. Mol. Biol.* **300**, 1005–1016 (2000).

111. Emanuelsson, O., Brunak, S., von Heijne, G. & Nielsen, H. Locating proteins in the cell using TargetP, SignalP and related tools. *Nat. Protoc.* **2**, 953–971 (2007).

112. Slater, G. S. C. & Birney, E. Automated generation of heuristics for biological sequence comparison. *BMC Bioinformatics* **6**, 31 (2005).

113. Li, F.-W. & Mathews, S. Evolutionary aspects of plant photoreceptors. *J. Plant Res.* **129**, 115–122 (2016).

114. Li, F.-W. *et al.* Horizontal transfer of an adaptive chimeric photoreceptor from bryophytes to ferns. *Proc. Natl. Acad. Sci. U. S. A.* **111**, 6672–6677 (2014).

115. Li, F.-W. *et al.* Phytochrome diversity in green plants and the origin of canonical plant phytochromes. *Nat. Commun.* **6**, 1–12 (2015).

116. Li, F.-W. *et al.* The origin and evolution of phototropins. *Front. Plant Sci.* **6**, 1–11 (2015).

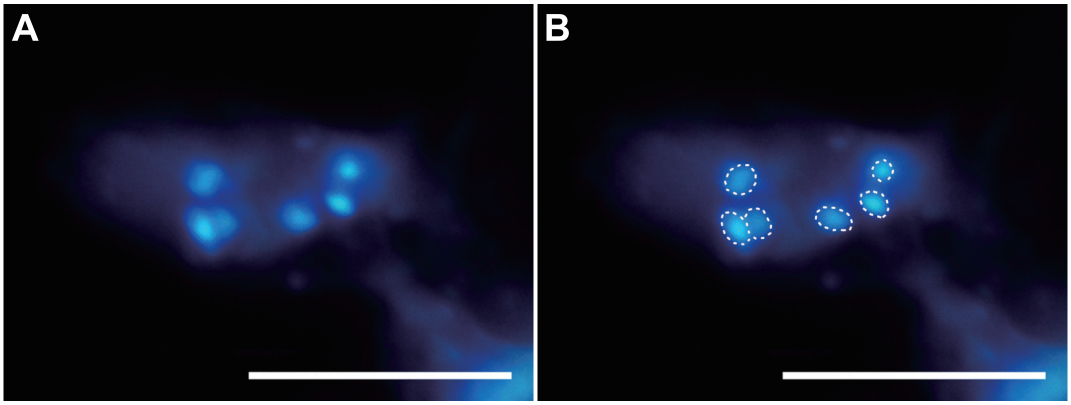

Supplementary Figure 1. **Mitotic chromosome count of *Anthoceros agrestis* (Oxford strain)**. (A) A total of 6 chromosomes can be distinguished. (B) Same image as (A) but with each chromosome outlined separately. The scale bar is 10μm.

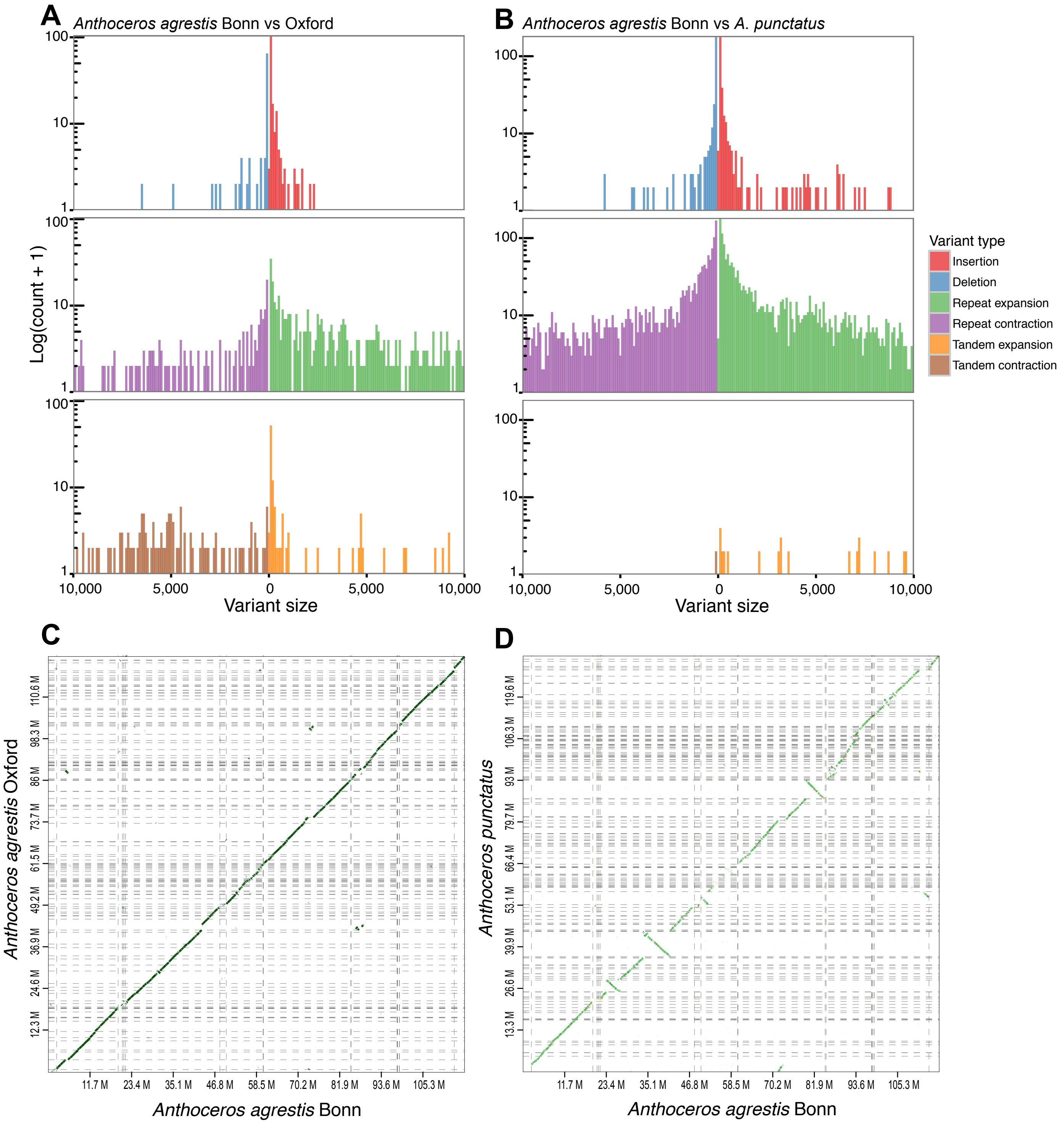

Supplementary Figure 2. **Collinearity of the three *Anthoceros* genomes**. Structural variation between **a**, *A. agrestis* Bonn and Oxford strains, and **b**, between *A. agrestis* Bonn and *A. punctatus*. Dot plot comparing **c**, *A. agrestis* Bonn to Oxford, and **d**, *A. agrestis* Bonn to *A. punctatus*.

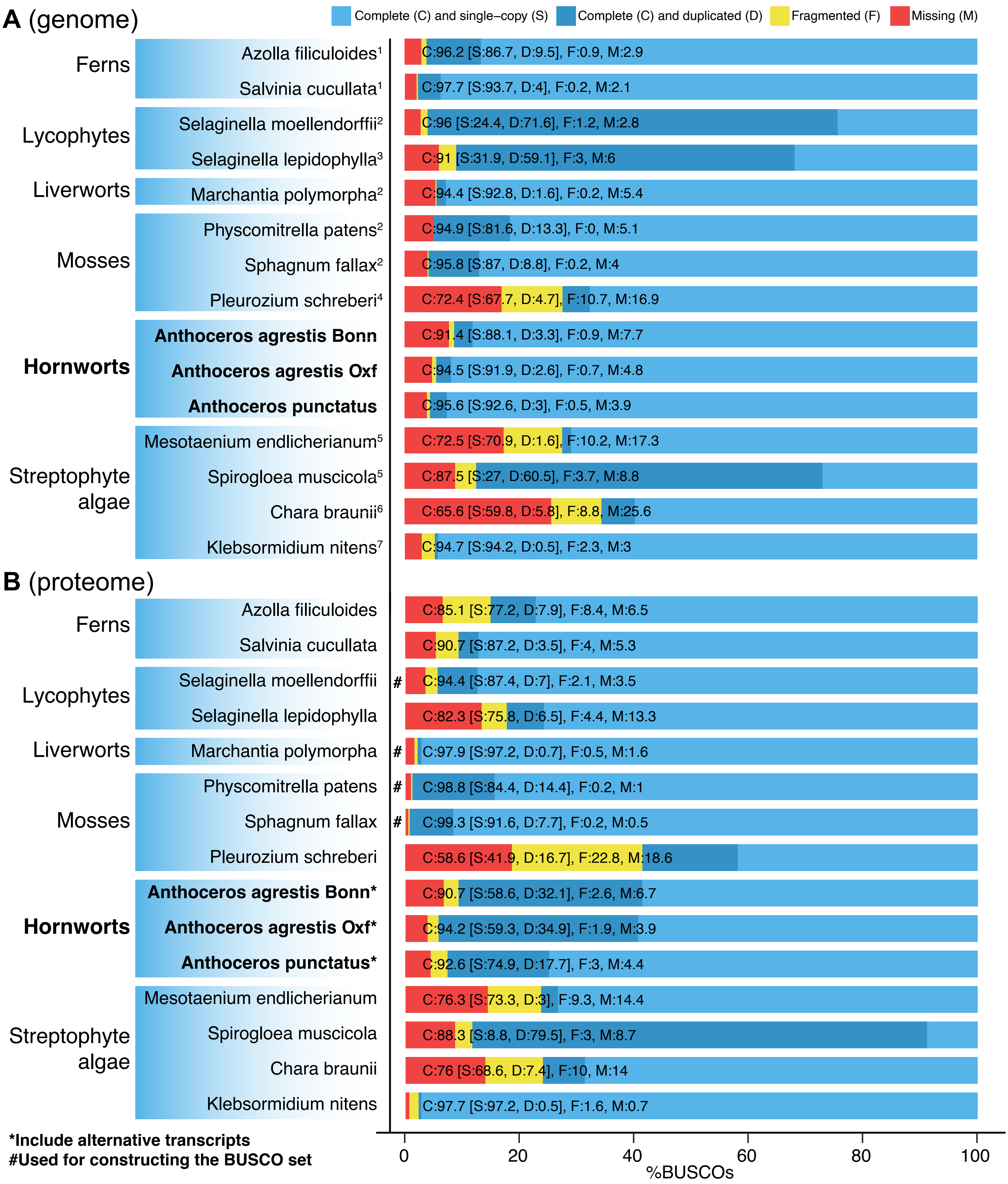

Supplementary Figure 3. **Comparison of BUSCO scores (Viridiplantae set)**. (A) Genome. (B) Annotated proteome. The hornwort proteomes include alternative transcripts and that is why they show high proportions of duplicated BUSCOs. The proteomes that form the basis of the Viridiplantae set are marked by #, which unsurprisingly show high degrees of completeness.

(1) Li, F.-W. et al. Fern genomes elucidate land plant evolution and cyanobacterial symbioses. Nature Plants 4, 1–16 (2018). (2) Goodstein, D. M. et al. Phytozome: a comparative platform for green plant genomics. Nucleic Acids Res. 40, D1178–86 (2012). (3) VanBuren, R. et al. Extreme haplotype variation in the desiccation-tolerant clubmoss Selaginella lepidophylla. Nat. Commun. 9, 13 (2018). (4) Pederson, E. R. A. et al. Genome sequencing of Pleurozium schreberi: The assembled and annotated draft genome of a Pleurocarpous feather moss. G3 9, 2791–2797 (2019). (5) Cheng, S. et al. Genomes of subaerial Zygnematophyceae provide insights into land plant evolution. Cell 179, 1057–1067.e14 (2019). (6) Nishiyama, T. et al. The Chara genome: secondary complexity and implications for plant terrestrialization. Cell 174, 448–464.e24 (2018). (7) Hori, K. et al. Klebsormidium flaccidum genome reveals primary factors for plant terrestrial adaptation. Nat. Commun. 5, 3978 (2014).

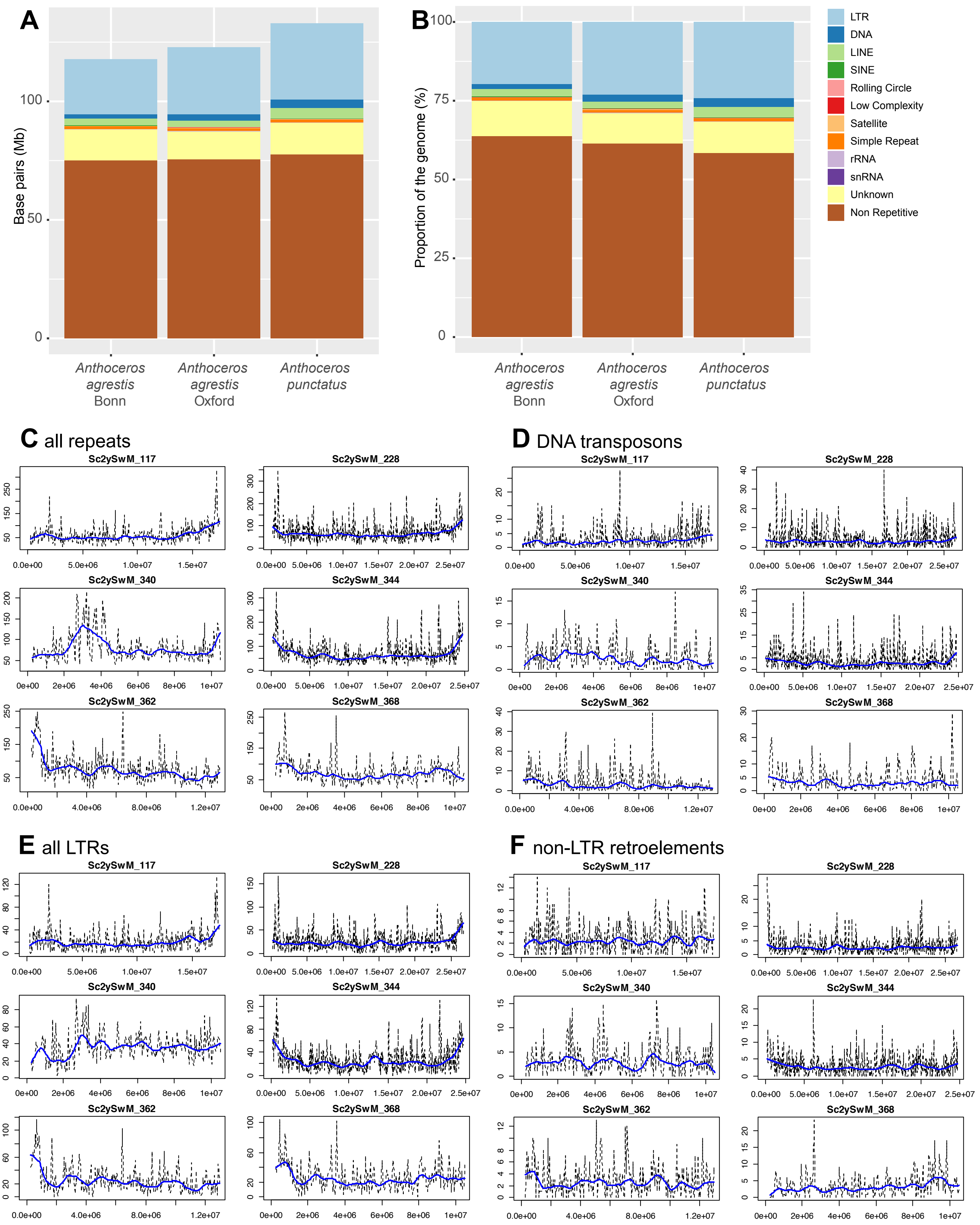

Supplementary Figure 4. **Repeat composition and distribution in the *Anthoceros* genomes**. Comparison of the three *Anthoceros* genomes in **a**, absolute repeat size, and **b**, repeat size as genome proportion. Repeat distributions along the six largest *A. agrestis* Bonn scaffolds: **c**, all repeats, **d**, DNA transposons, **e**, all LTR, **f**, non-LTR retroelements, **g**, Gypsy, **h**, Copia, **i**, LINE, **j**, SINE, **k**, LINE-L1.

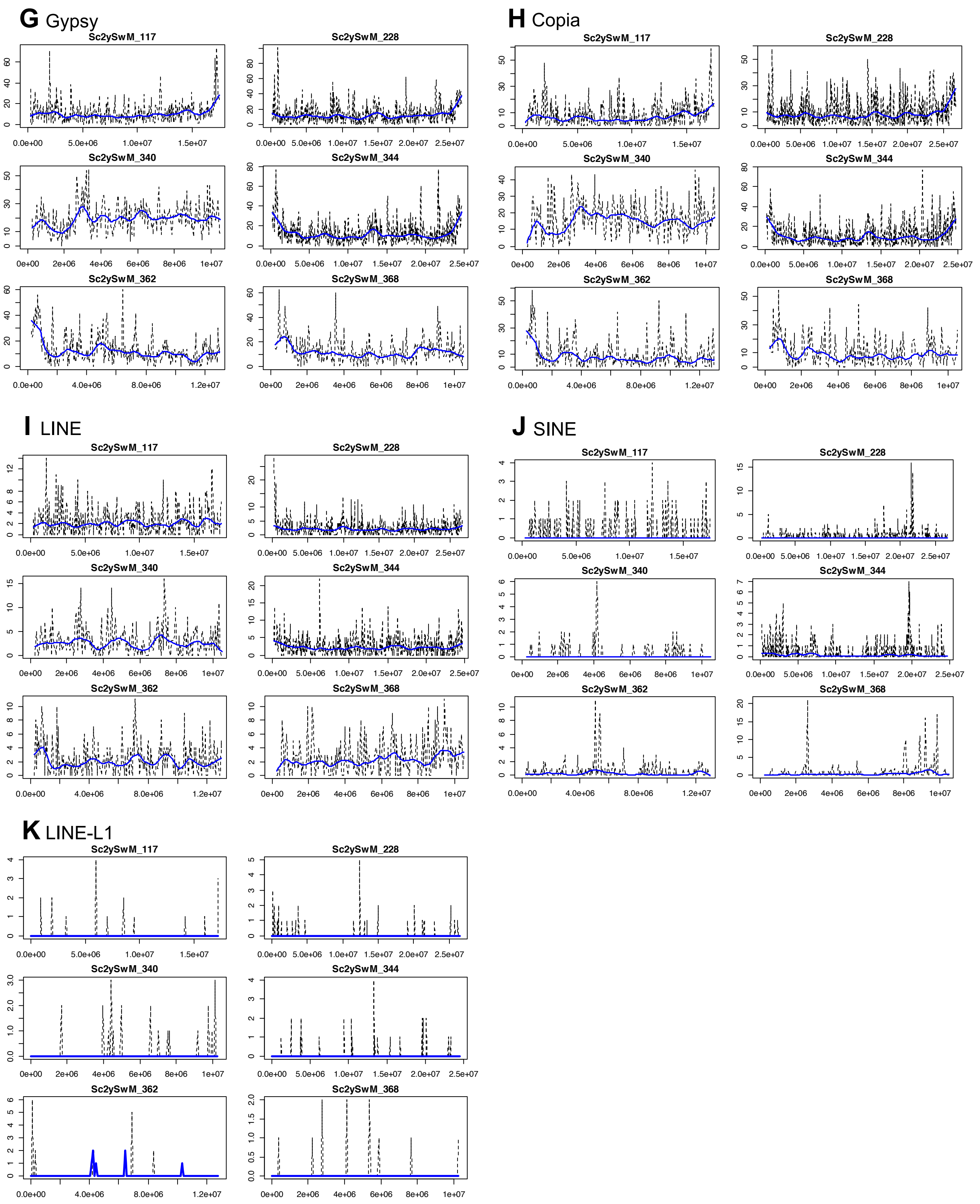

Supplementary Figure 4. **Repeat composition and distribution in the *Anthoceros* genomes**. continued**.**

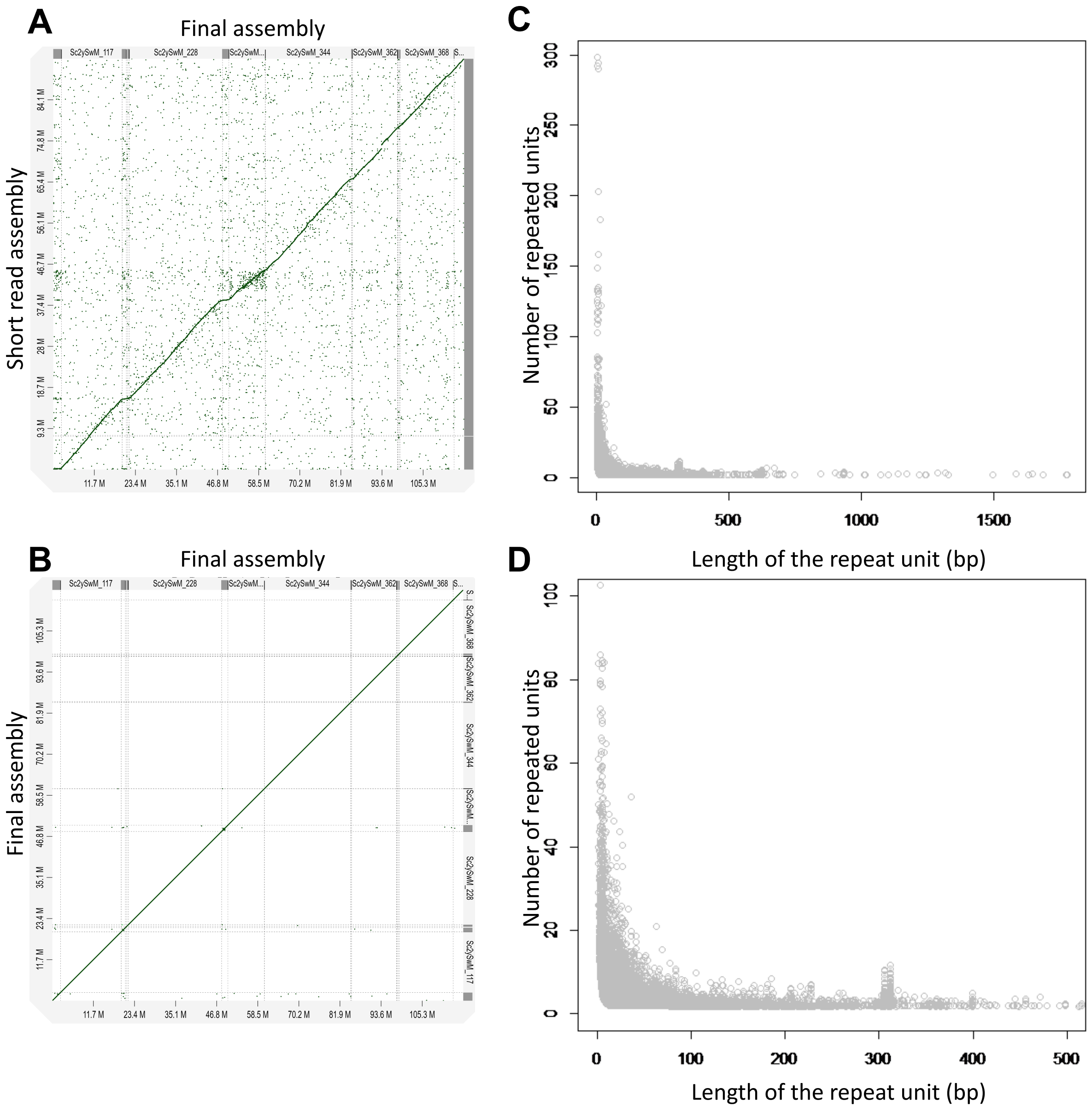

Supplementary Figure 5. **The *Anthoceros* genomes lack long tandem repeats**. **a**, Dotplot comparing Illumina short-read and final chromosomal assemblies. **b**, Self synteny of the final chromosomal assembly. **c**, The number of repeated units plotted against the length of the unit. **d**, Same as **c** but the x-axis is cutoff at 500bp. **e**, Distribution of tandem repeats along the six largest scaffolds in *A. agrestis* Bonn genome. There is no long tandem repeats that are spatially clustered. Position in Mb. **f**, Same as **e** but only the repeats with unit length ≥10bp and duplicated ≥30 times were plotted.

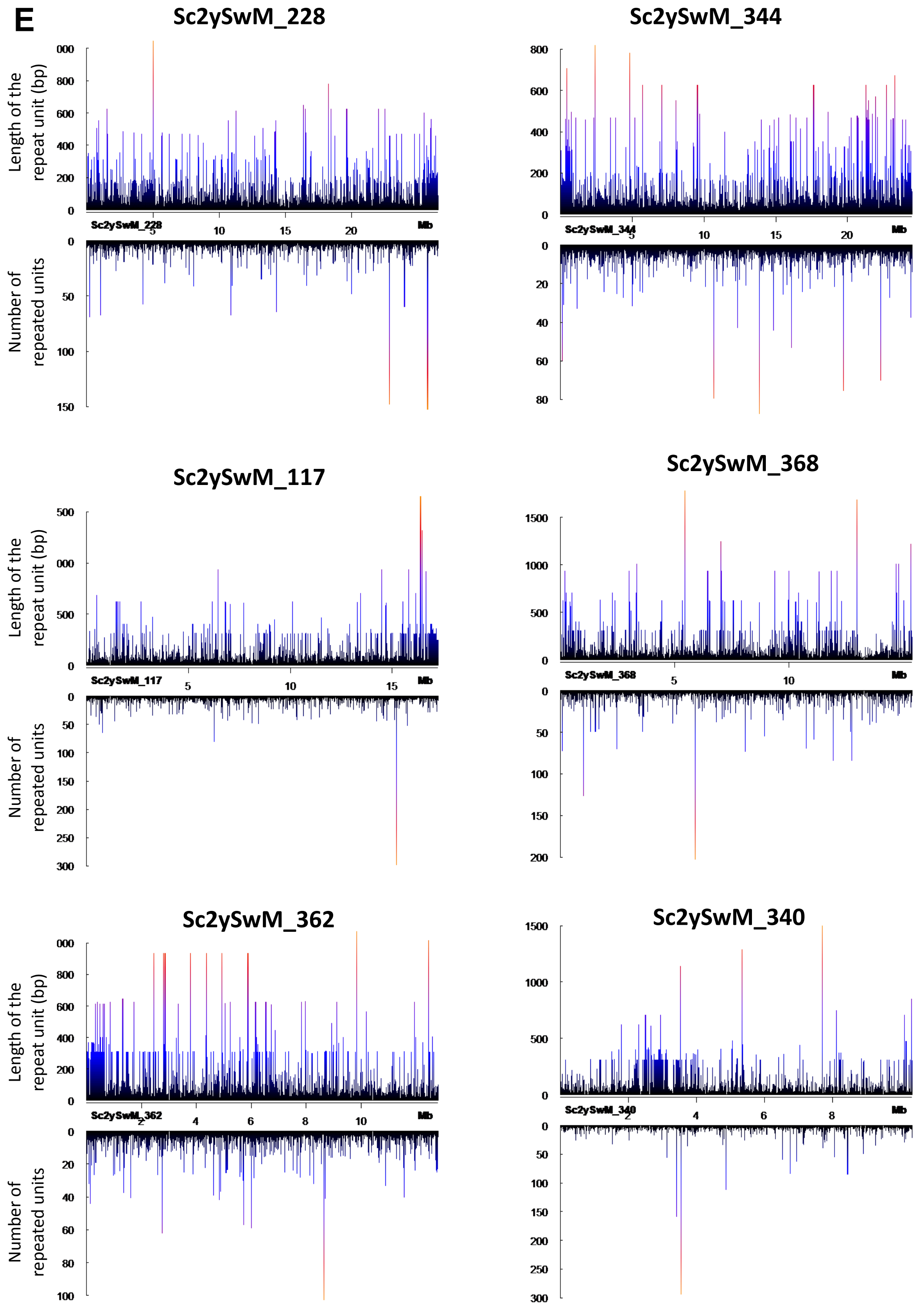

Supplementary Figure 5. **The *Anthoceros* genomes lack long tandem repeats**. continued**.**

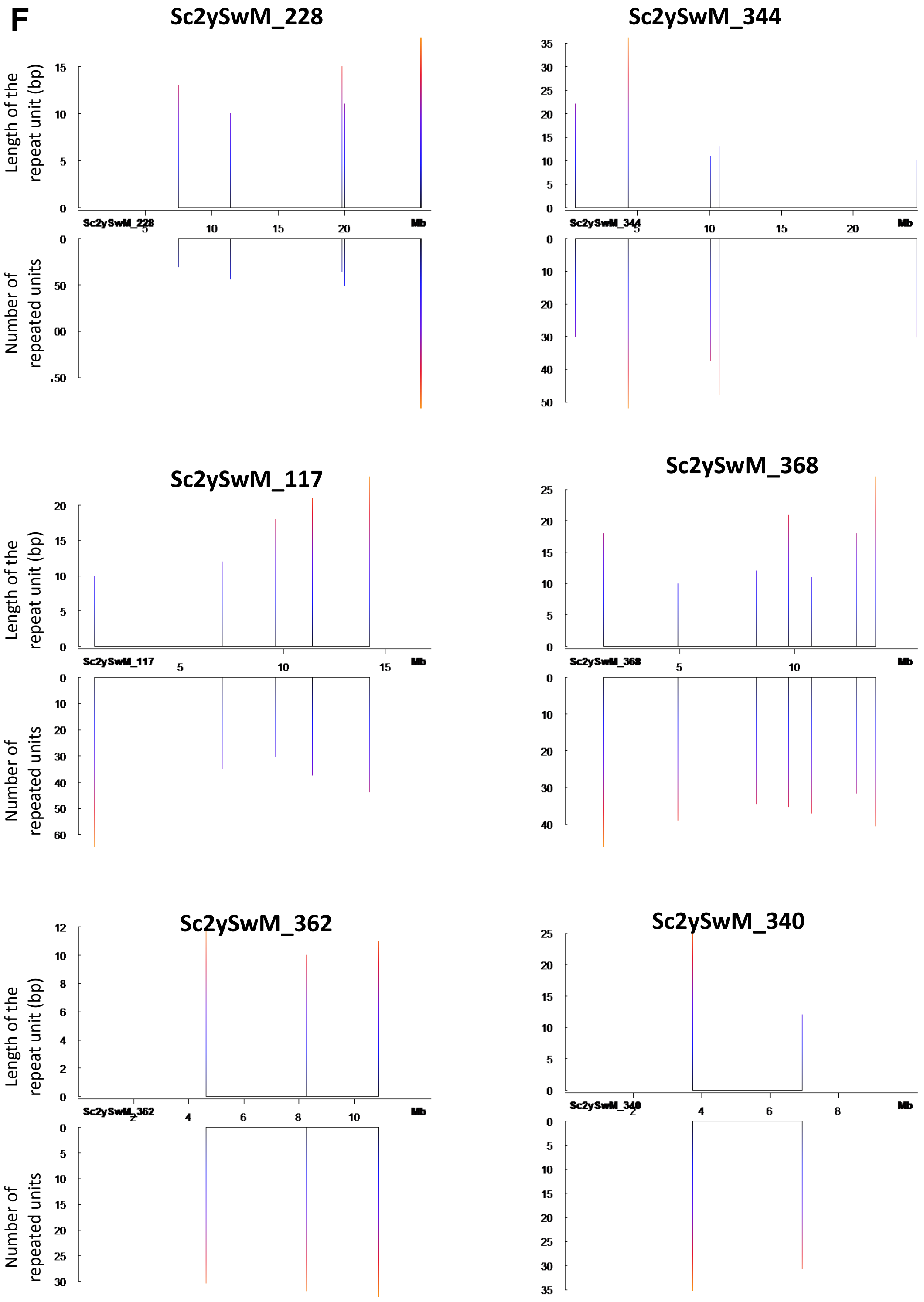

Supplementary Figure 5. **The *Anthoceros* genomes lack long tandem repeats**. continued.

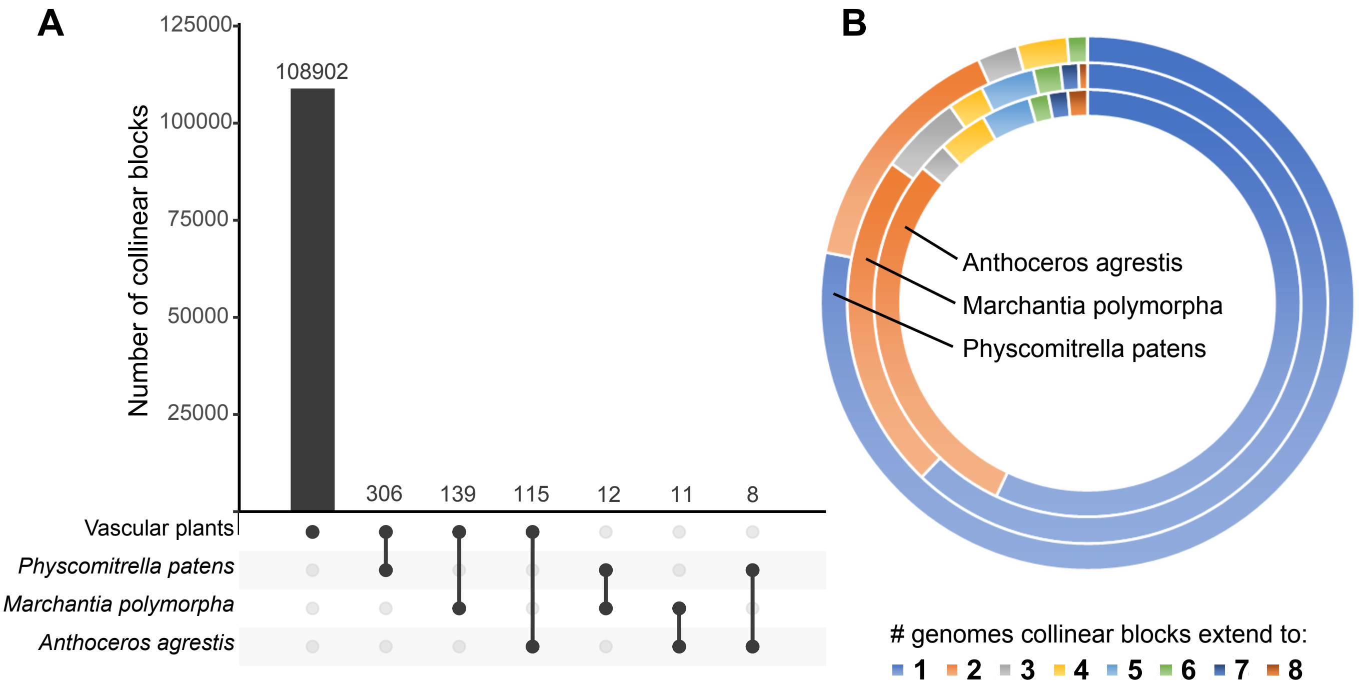

Supplementary Figure 6. **Collinearity of land plant genomes**. **a**, Number of collinear blocks within vascular plants, as well as between each of the bryophyte lineages and vascular plants. Collinearity with any one of the vascular plant genomes (see Supplementary Table 7) was counted. **b**, The proportion of genomic segments in the three bryophyte genomes collinear across 1-8 other genomes. IAdHore3 was run with a minimum of three anchors per collinear blocks.

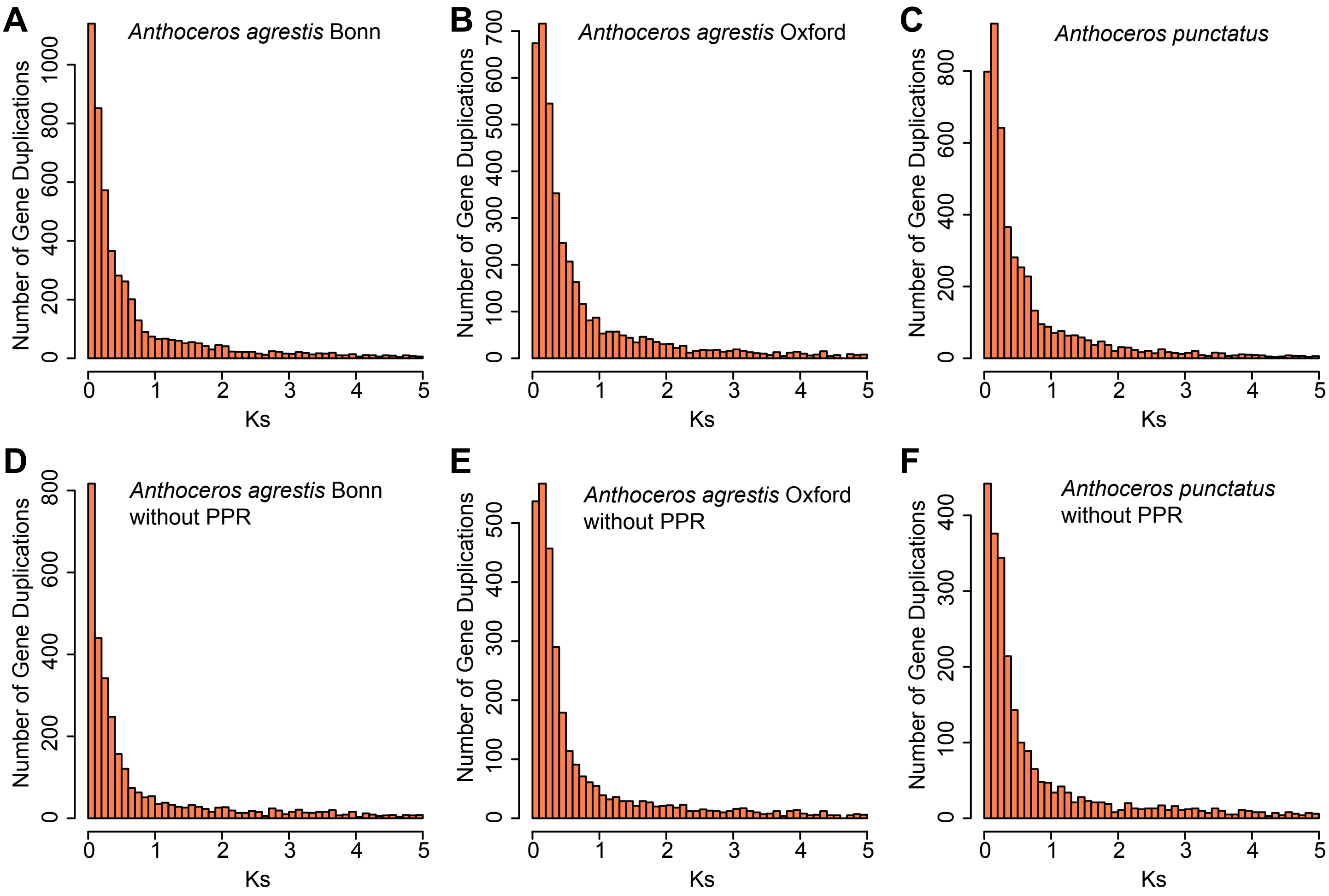

Supplementary Figure 7. **No strong signal of whole genome duplication from Ks divergence**. Ks plots of the annotated genes from **a,** *Athoceros agrestis* Bonn, **b,** *A. agresits* Oxford, and **c,** *A. punctatus*. Because pentatricopeptide repeat protein (PPR) genes have proliferated in hornworts and might create artificial low Ks peaks, we additionally generated Ks plots with PPR genes removed (**d-f**).

A
YABBY

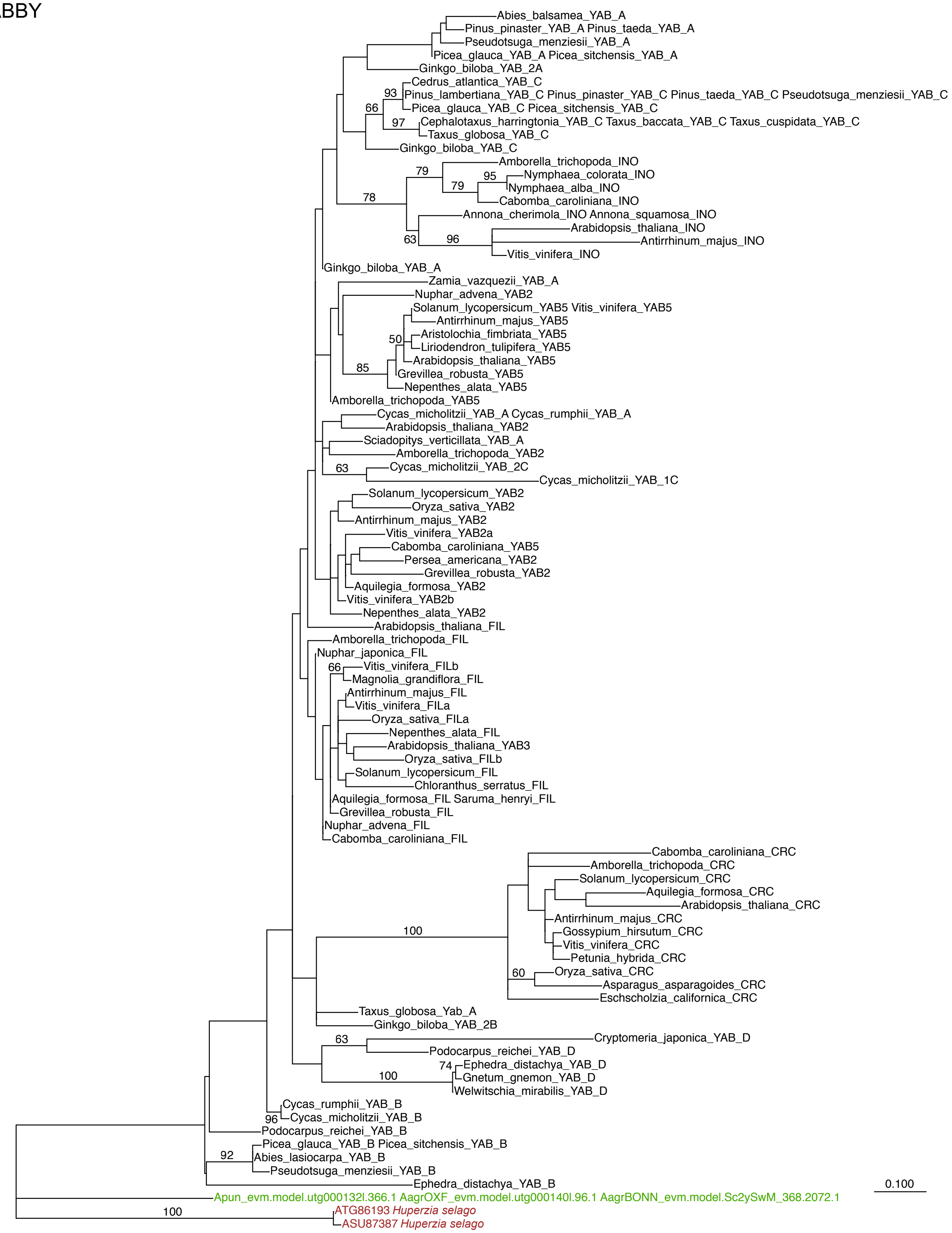

Supplementary Figure 8. **Phylogeny of key sporophyte development genes**. **a,** YABBY. **b,** KNOX. **c,** WOX. **d,** LEAFY. **e**, A part of the LEAFY amino acid sequence alignment confined to the DNA binding domain. Amino acid numbering is based on AtLFY. Black triangle indicates residues that are reportedly involved in DNA binding specificity (Sayou et al. 2014). Hornwort sequences (in green) were collapsed as one terminal tip if they are identical. Bootstrap support values, if >50, are shown along the branches.

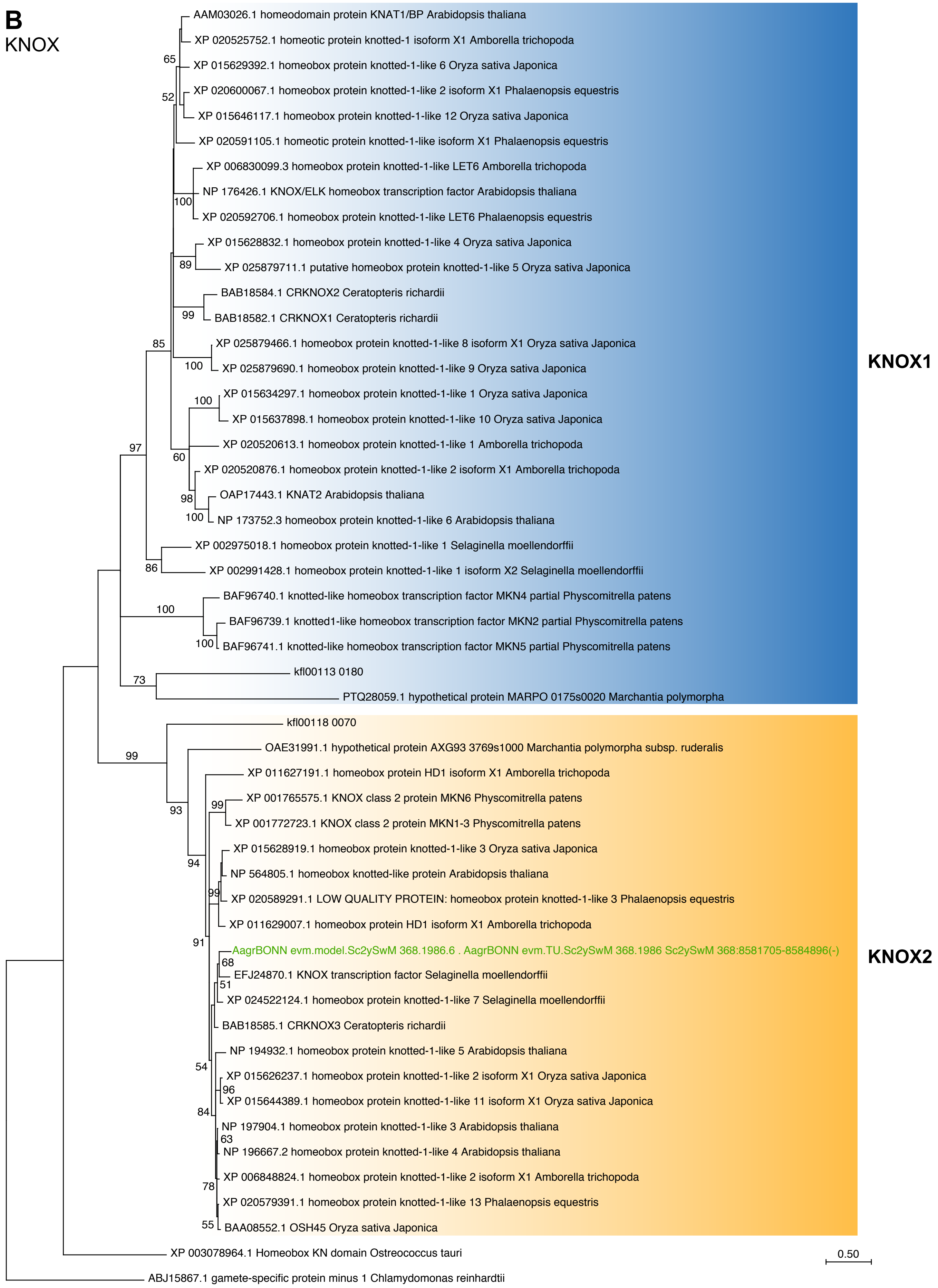

Supplementary Figure 8. **Phylogeny of key sporophyte development genes.** continued.

C
WOX

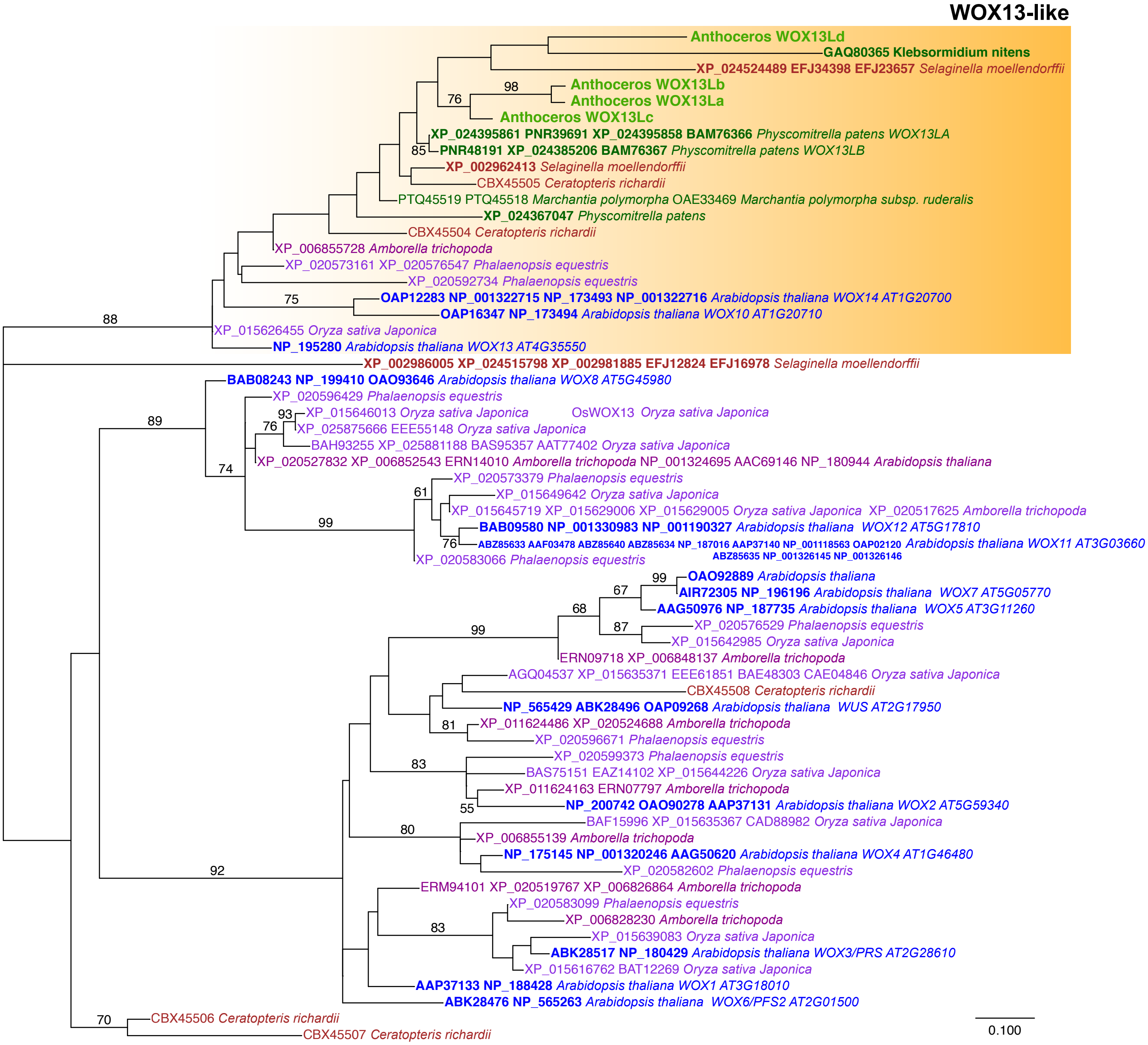

Supplementary Figure 8. **Phylogeny of key sporophyte development genes.** continued.

D

LEAFY

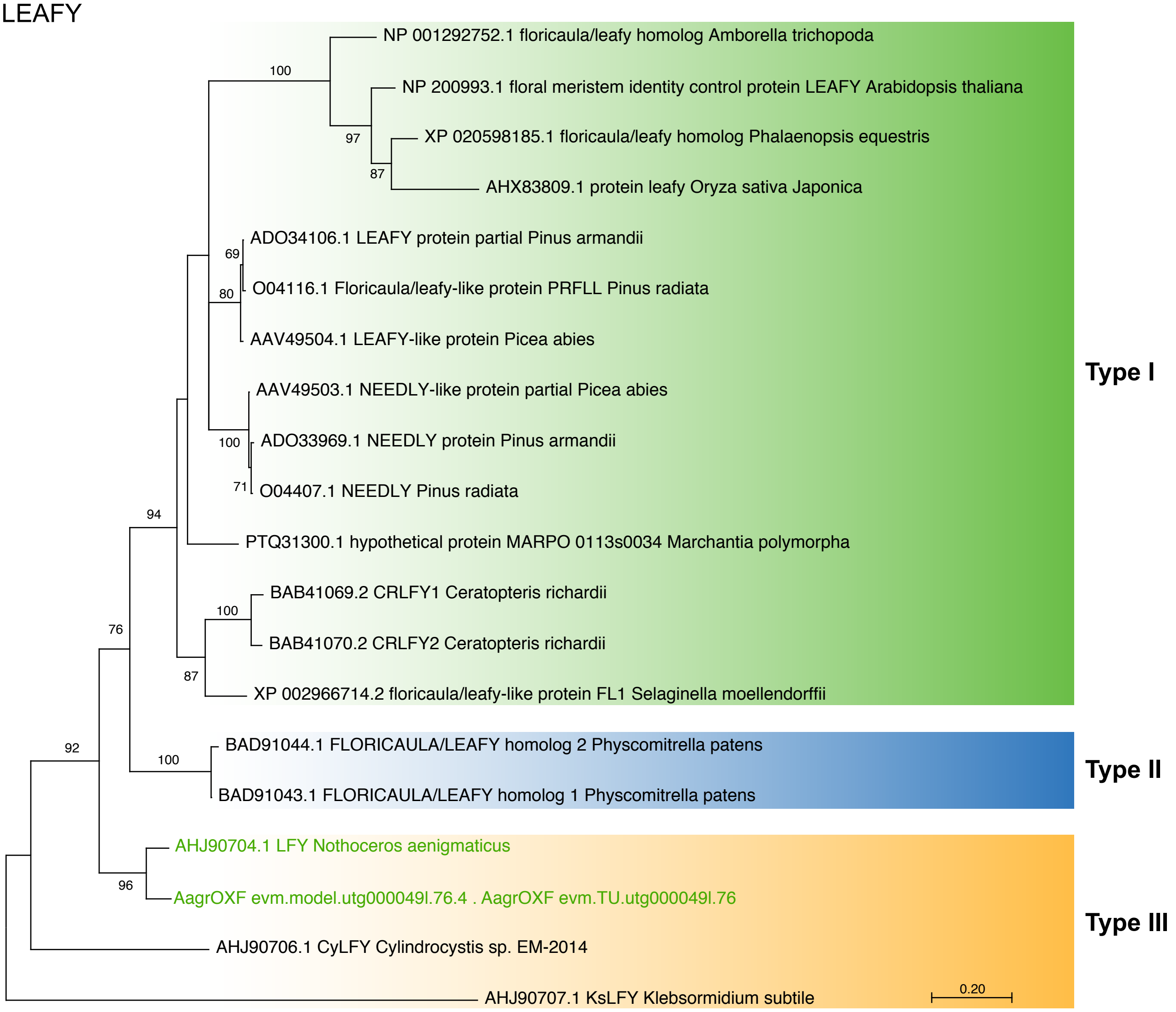

E

```
                                                240       250       260       270       280       290        300       310       320        330       340
                                                  .         .         .         .         .         .          .         .         .          .         .
NP_200993.1 LFY [Arabidopsis thaliana]           RQREHPFIVTEPGEVARGKKNGLDYLFHLYEQCREFLLQVQTIAKDRGEKCPT-KVTNQVFRYAKKSGASYINKPKMRHYVHCYALHCLDEEASNALRRAFKERGE-----
XP_020598185.1 [Phalaenopsis equestris]          RQREHPFIVTEPGEVARAKKNGLDYLFHLYEQCHEFLLQVQAVAKDRGDKCPT-KVTNLVFRYAKKVGASYINKPKMRHYVHCYALHVLDEEASNALRRAFKERGE-----
AHX83809.1 leafy [Oryza sativa]                  RQREHPFVVTEPGEVARAKKNGLDYLFHLYEQCRLFLLQVQSMAKLHGHKSPT-KVTNQVFRYAKKVGASYINKPKMRHYVHCYALHCLDEEASDALRRAYKAPGE-----
NP_001292752.1 flo/leafy [Amborella trichopoda]  RQREHPFIVTEPGEVARGKKNGLDYLFHLYDECRNFLIQVQALAKERGEKCPN-KVTNQVFRHAKRAGASHINKPKMRHYVHCYALHCLDEEASNALRRAFKERGE-----
O04116.1 PRFLL [Pinus radiata]                   RQREHPFIVTEPGELARGKKNGLDYLFDLYEQCGKFLLDVQHIAKERGEKCPT-KVTNQVFRHAKHSGAGYINKPKMRHYVHCYALHCLDIEQSNRLRRAYKERGE-----
O04407.1 NEEDLY [Pinus radiata]                  RPREHPFIVTEPGELARGKKNGLDYLFDLYEQCGKFLLEVQRIAKEKGEKCPT-KVTNQVFRHAKHNGAVYINKPKMRHYVHCYALHCLDSEQSNHLRRLYKERGE-----
BAB41069.2 CRLFY1 [Ceratopteris richardii]      RTREHPFIVTEPGEVARGKKNGLDYLFDLYEQCARFLDEVQQMARERGEKCPT-KVTNQVFRHAKLKGASYINKPKMRHYVHCYALHCLDKEKSNFLRKQFKERGE-----
BAB41070.2 CRLFY2 [Ceratopteris richardii]      RTREHPFIVTEPGEVARGKKNGLDYLFDLYEQCARFLDEVQQMARERGEKCPT-KVTNQVFRHAKLKGASYINKPKMRHYVHCYALHCLDKDKSNFLRKQFKERGE-----
XP_002966714.2 FL1 [Selaginella moellendorffii] RPREHPFIVTEPGELARGKKNGLDYLFDLYEQCGRYLDEVQQQCKERGEKCPT-KVTNQVFRHAKHRGASYINKPKMRHYVHCYALHCLDLEQSNHLRKVFKDRGE-----
PTQ31300.1 MARPO_0113s0034 [Marchantia polymorpha] RPREHPFIVTEPGEAARGKKNGLDYLFDLYEQCGKFLEQVQQLAREKGEKCPT-KVTNQVFRHAKHTGAGYINKPKMRHYVHCYALHCLDIDQSNALRKLYKERGE-----
BAD91043.1 FLO/LEAFY 1 [Physcomitrella patens]  RPREHPFIVTEPGEPAKGKKNGLDYLFDLYEQCGKFLEEVQHIAKEKGEKCPS-KVTNEVFRHAKLTGAGYINKPKMRDYVHCYALHCLDVETSNNLRKEYKERGE-----
BAD91044.1 FLO/LEAFY 2 [Physcomitrella patens]  RPREHPFIVTEPGEPAKGKKNGLDYLFDLYEQCGKFLEEVQHIAKEKGEKCPS-KVTNEVFRHAKLTGAGYINKPKMRDYVHCYALHCLDVETSNNLRKEFKERGE-----
AHJ90704.1 LFY [Nothoceros aenigmaticus]        RPREHPFVVTEPGELARGKKNGLDYLFNLYEQAGKFLEEVQHIAREKGEKCPT-KVTNQVFRHAKVQGAGYINKPKMRQYVHCYALHCLASDKSDELRRCCKERGE-----
AagrOXF_evm.TU.utg0000491.76                     RPREHPFVVTEPGELARGKKNGLDYLFNLYEQAGKYLEEVQLIAREKGEKCPT-KVTNQVFRHAKLQGAGYINKPKMRQYVHCYALHCLASAESDELRRSSKERGE-----
AHJ90706.1 CyLFY [Cylindrocystis sp. EM-2014]   RPREHPFQVTERGEEARGKRNGLDHLFELYEEAGQLLVELQQTCSQTGAKWPT-KVNNQVFRYAKAKGMGHINKPKMRQYVHCYALHCLDVVRSDDLRREFKFKSEAHPRP
AHJ90707.1 KsLFY [Klebsormidium subtile]        TSRGPAFQVTAFGEEGHGKKQGLDYLFELYGEAGRILEDIRAQERATGKK-PSAKVNNLVYRYAKQRGMGFINKPKMRQYLHCYALHCLDVGTSNAIRMTCRDRNK-----
                                                                                                                      ▲

                                                   350       360       370       380       390
                                                     .         .         .         .         .
NP_200993.1 LFY [Arabidopsis thaliana]           NVGSWRQACYKPLVNIA-CRHGWDIDAVFNAHPRLSIWYVPTKLRQLCHLERNNA--
XP_020598185.1 [Phalaenopsis equestris]          NVGAWRLACYKPLVAIS-AAHSFDIDAVFNAHPRLSIWYVPTKLRQLCHLARSSSQP
AHX83809.1 leafy [Oryza sativa]                  NVGAWRQACYAPLVDIS-ARHGFDIDAVFAAHPRLAIWYVPTRLRQLCHQARSSH--
NP_001292752.1 flo/leafy [Amborella trichopoda]  NVGAWRQACYHPLVAMSRHHHAWDVDALFASHPSLAIWYVPTKLRQLCHADRHSVT-
O04116.1 PRFLL [Pinus radiata]                   NVGAWRQACYYPLVAMA-KDNGWDIEGVFNKHEKLRIWYVPTKLRQLCHLEK-----
O04407.1 NEEDLY [Pinus radiata]                  NVGAWRQACYYPLVAIA-RENNWDIEGIFNRNEKLKIWYVPTKLRQLCHMER-----
BAB41069.2 CRLFY1 [Ceratopteris richardii]      NVGAWRQACYYPLVDMA-RDNGWDIEGVFARNEKLRIWYVPTRLRQLCHLEK-----
BAB41070.2 CRLFY2 [Ceratopteris richardii]      NVGAWRQACYFPLVDMA-RDNGWDIEGVFVRNEKLRIWYVPTKLRQLCHFEK-----
XP_002966714.2 FL1 [Selaginella moellendorffii] NVGAWRQACYYPLVEMA-RNLNWDIEGVFSRNDKLRIWYVPTRLRQLCHLEK-----
PTQ31300.1 MARPO_0113s0034 [Marchantia polymorpha] NVGAWRGACYYPLVAMA-RDNNWDIEGLFNRNEKLRIWYVPTKLRQLCHVEK-----
BAD91043.1 FLO/LEAFY 1 [Physcomitrella patens]  NVGAWCQACYFPLVKLA-RQNEWDIDDLFNRNDKLRIWYVPTKLRQLCHIER-----
BAD91044.1 FLO/LEAFY 2 [Physcomitrella patens]  NVGAWCQACYFPLVTLA-RQNEWDIDDLFNQNDKLRIWYVPTKLRQLCHIER-----
AHJ90704.1 LFY [Nothoceros aenigmaticus]        NVGAWCQACYLPLIKMA-KEKSWDIEGLFNSHDKLKIWYVPKKLIQLCHQEK-----
AagrOXF_evm.TU.utg0000491.76                     NVGAWCQACYQPLVKMA-REKAWDIEGLFNSHEKLRIWYVPKKLIQLCHAEK-----
AHJ90706.1 CyLFY [Cylindrocystis sp. EM-2014]   NVGTWSDACYAPLVQMS-RDNNWDVDSLFENNEKLRIWYVPKK--------------
AHJ90707.1 KsLFY [Klebsormidium subtile]        TLQAWAECCYEPLLQMA-RVRGYNLESLFQLSPALAIWNVPKQLEKLCEEEK-----
                                                     ▲
```

Supplementary Figure 8. **continued.**

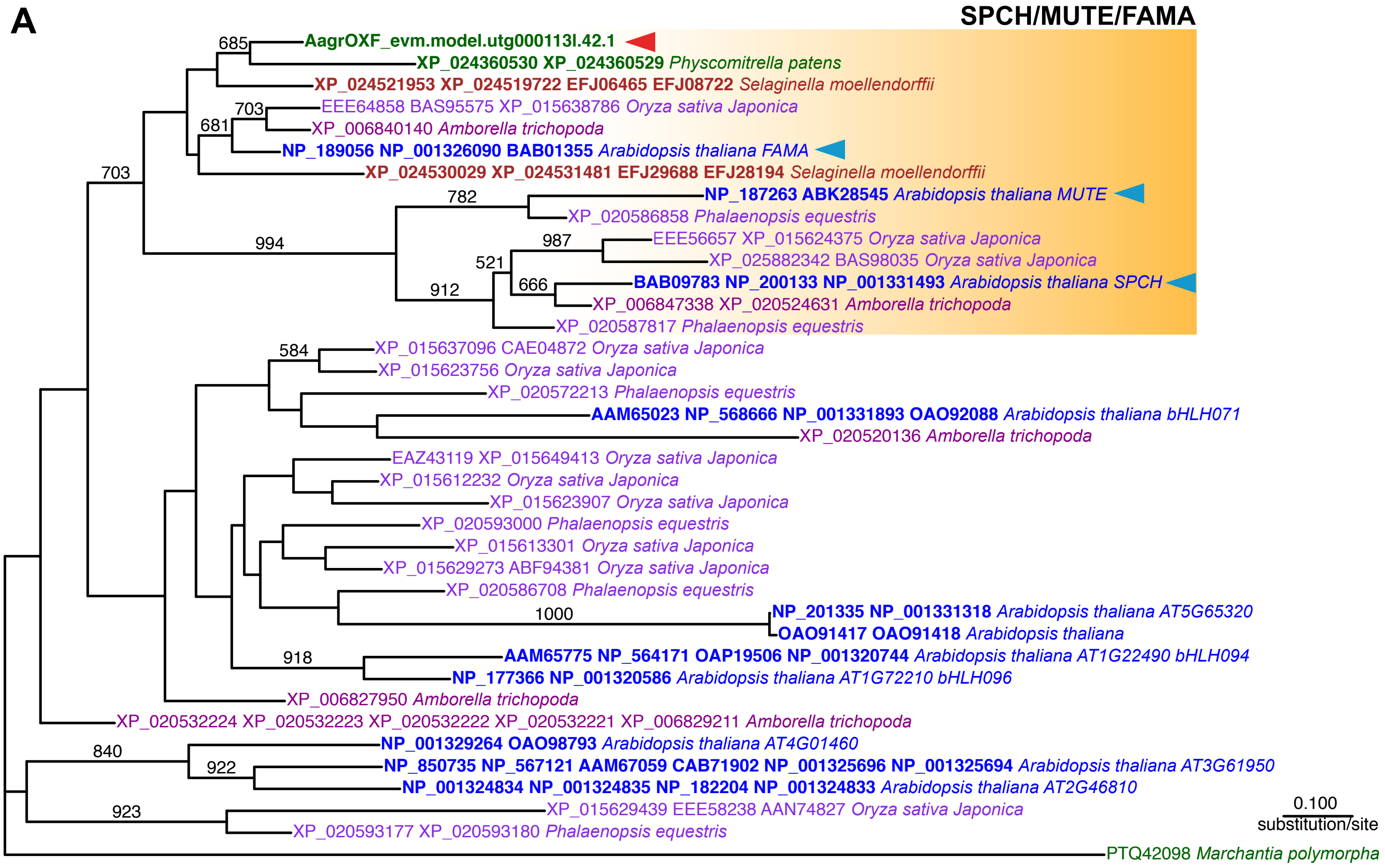

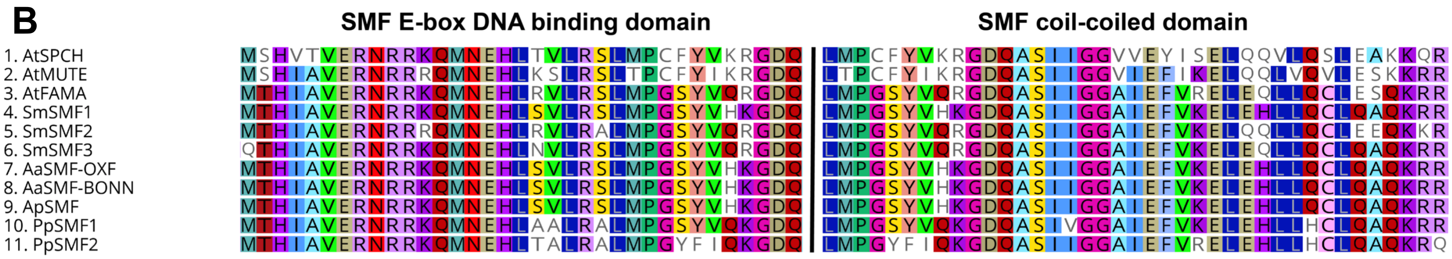

Supplementary Figure 9. **Phylogeny of stomata-related genes**. **a,** SPCH/MUTE/FAMA (SMF). **b**, Key SMF domain sequences are conserved in the hornwort homologs. **c**, ICE/SCRM. **d**, Key ICE/SCRM domain sequences are conserved in the hornwort homologs. **e**, ERECTA. **f**, TMM. **g**, EPF-like. Bootstrap values per 1000 replication are indicated on branches with bootstrap value > 500. Red and blue arrowheads mark the hornwort and Arabidopsis orthologs, respectively.

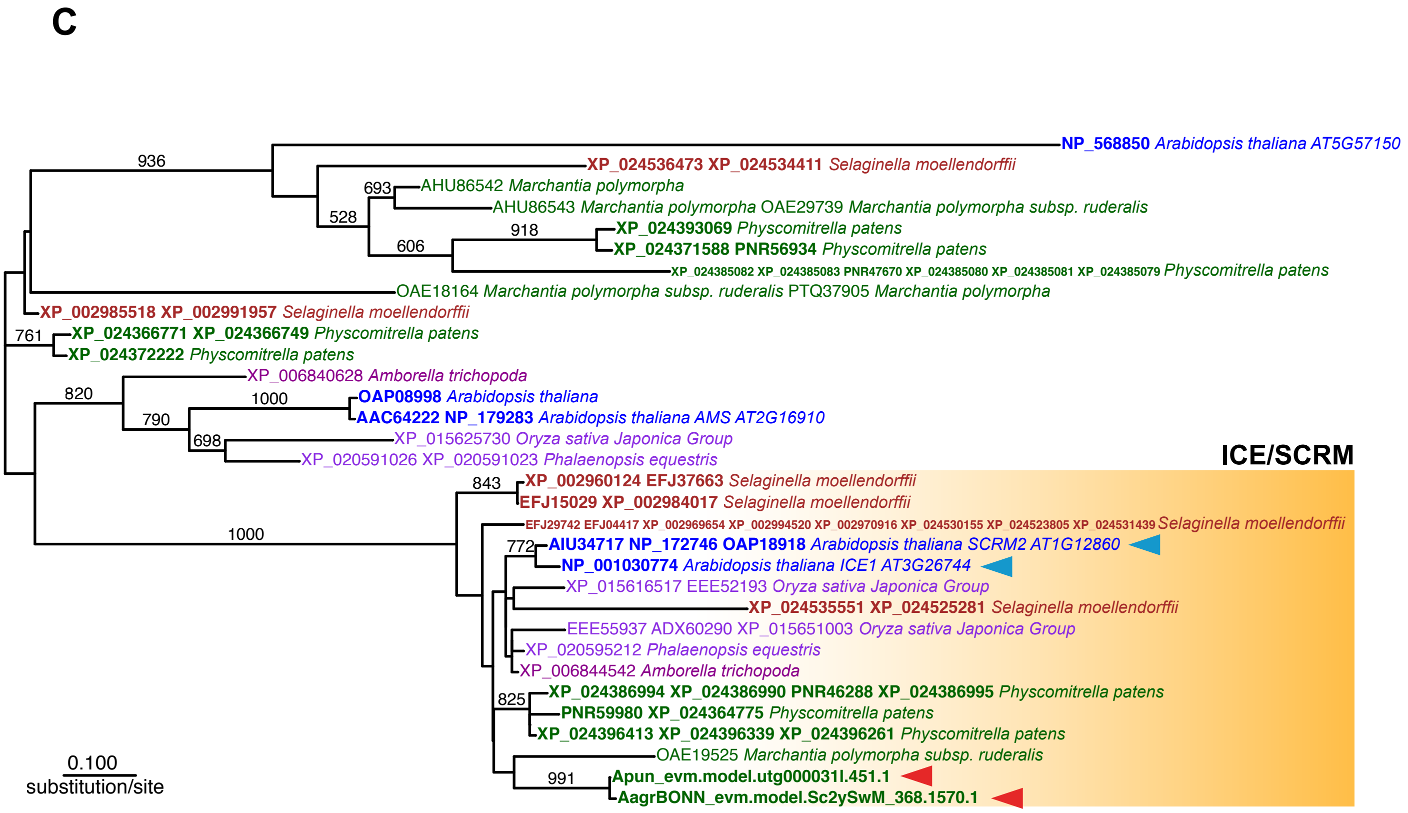

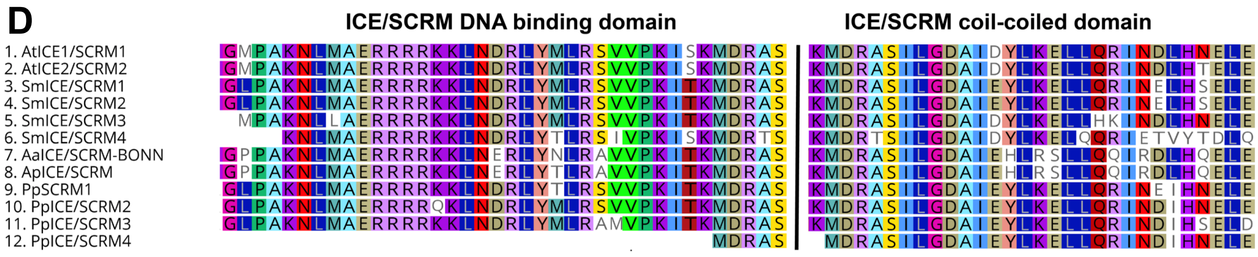

Supplementary Figure 9. **Phylogeny of stomata-related genes**. continued.

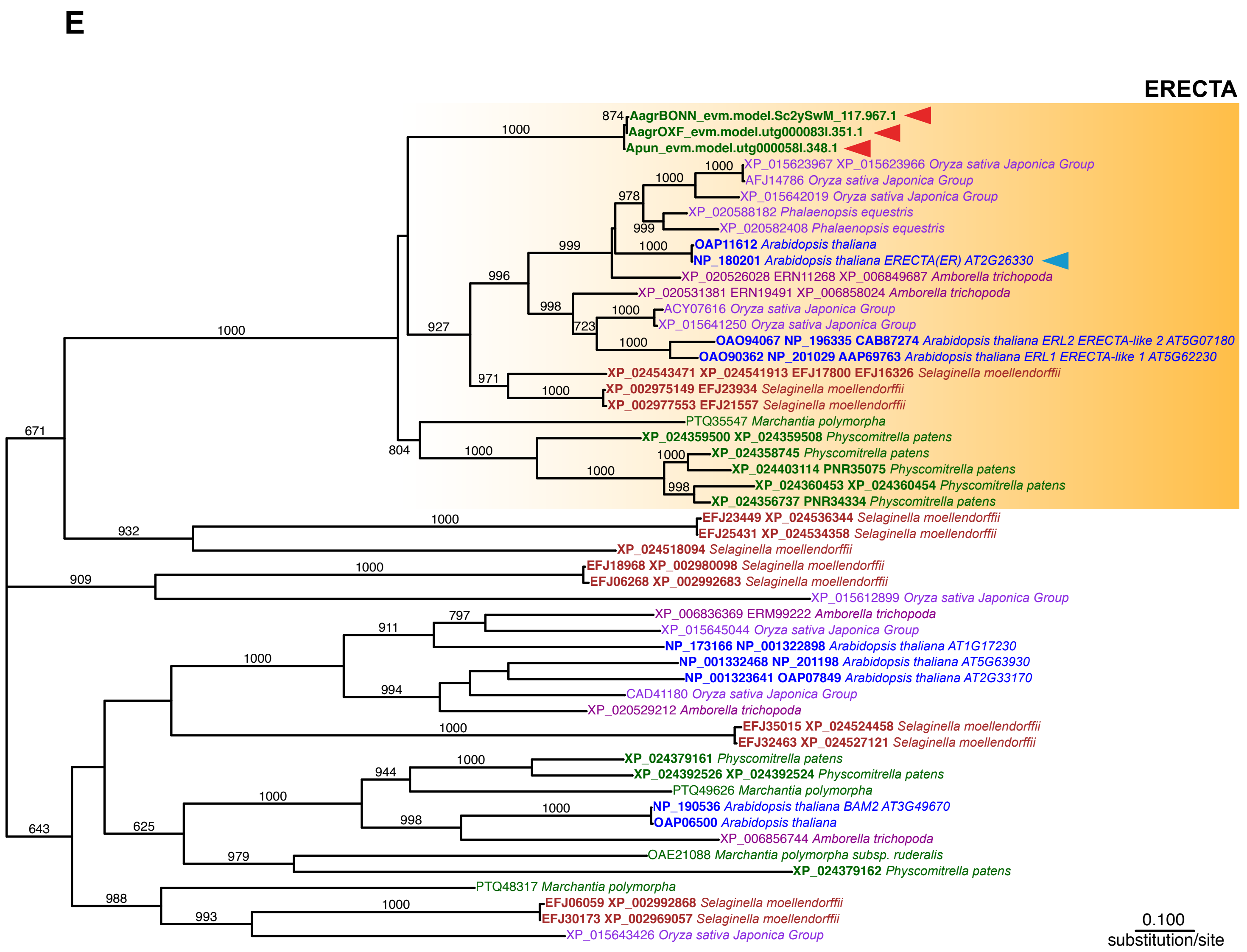

Supplementary Figure 9. **Phylogeny of stomata-related genes**. continued.

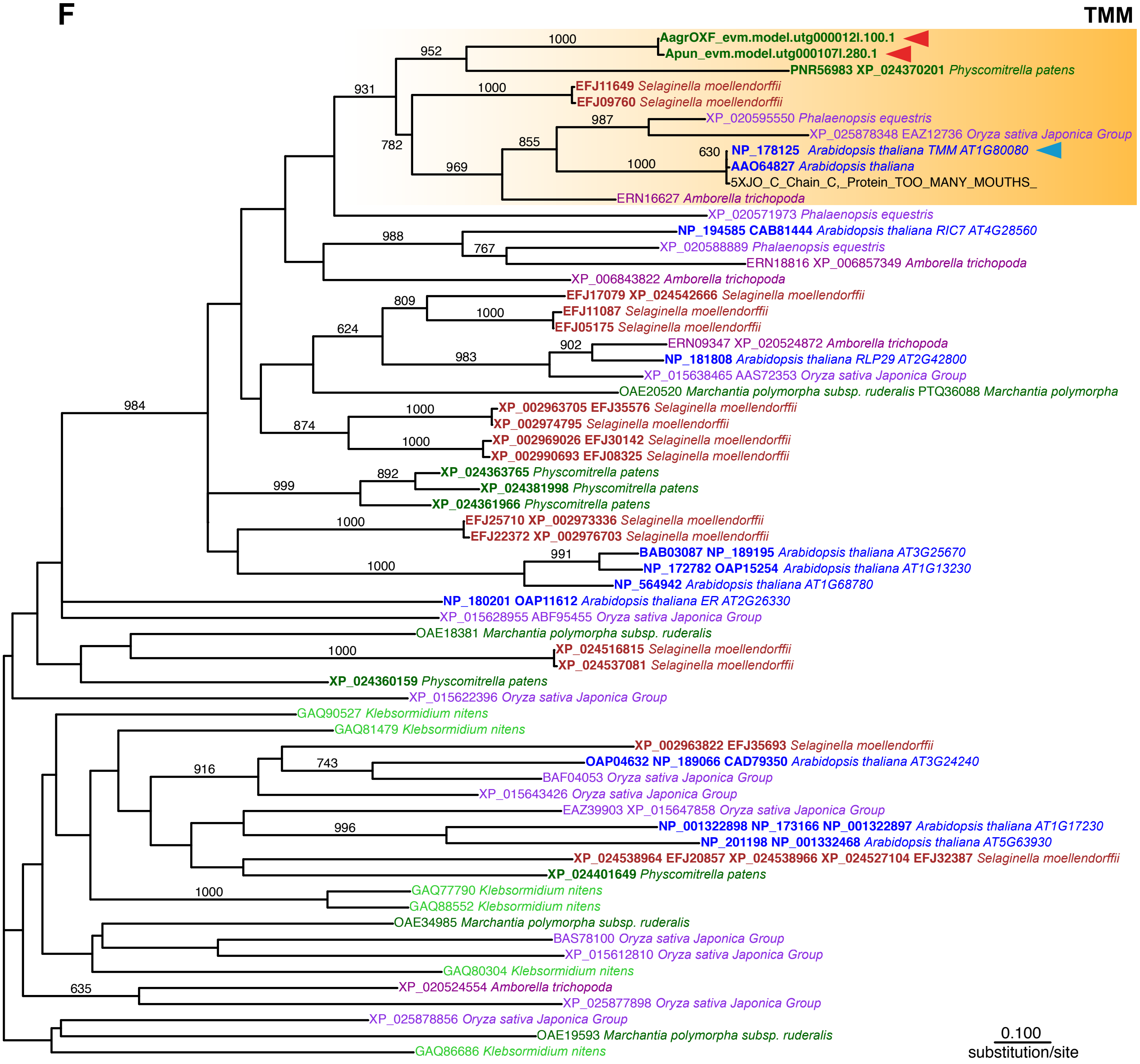

Supplementary Figure 9. **Phylogeny of stomata-related genes**. continued.

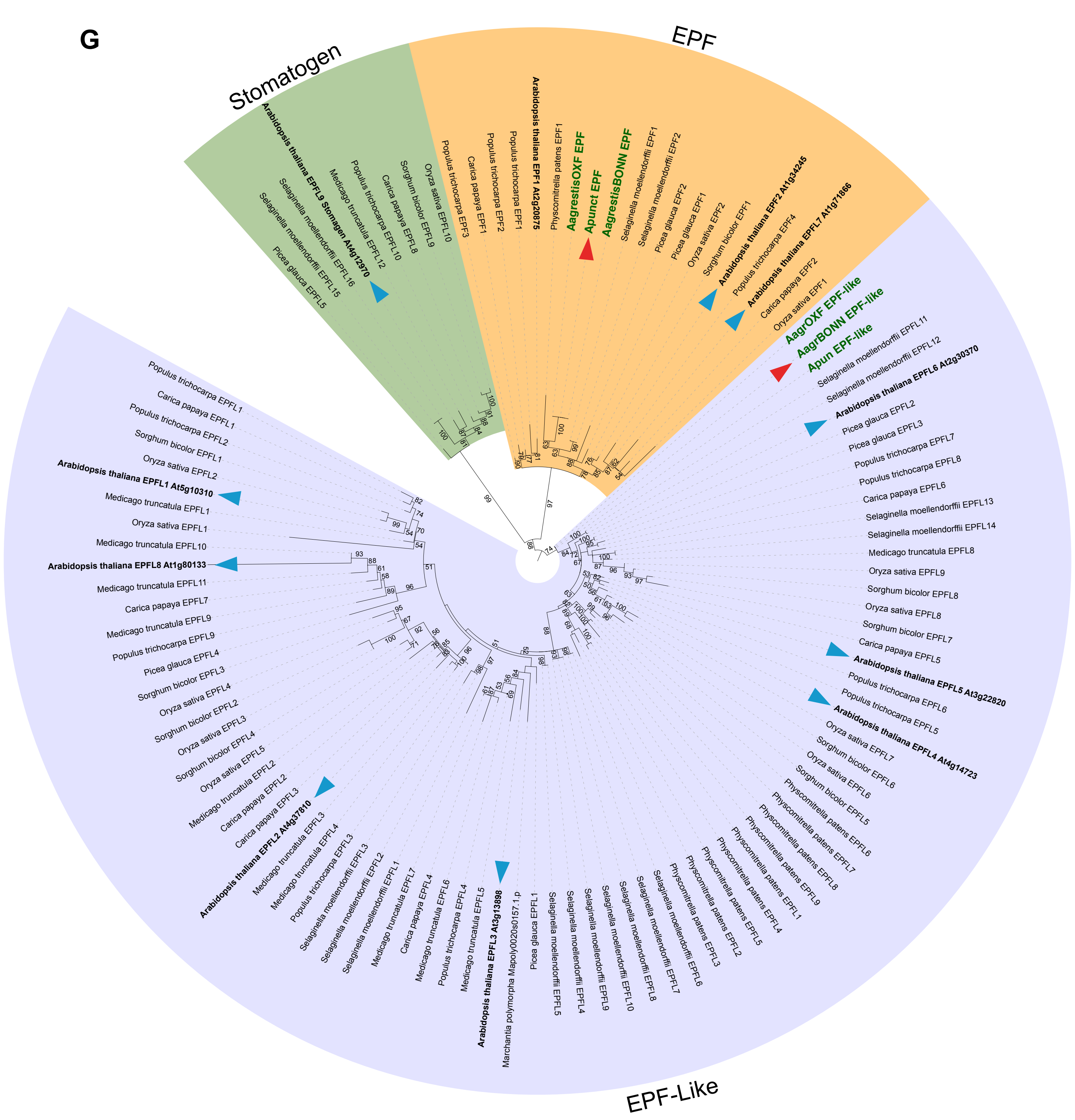

Supplementary Figure 9. **Phylogeny of stomata-related genes**. continued.

A

CO1

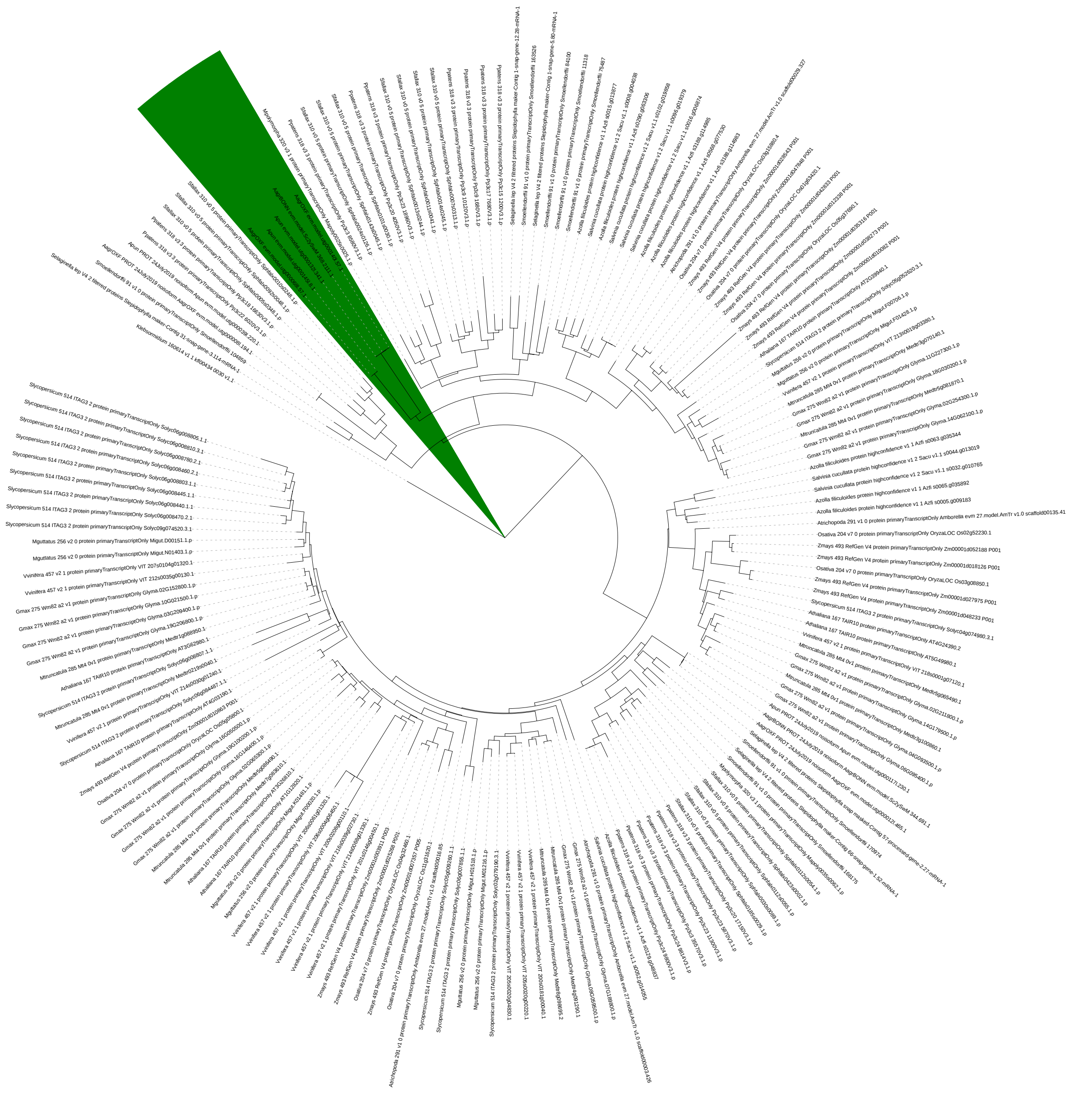

Supplementary Figure 10. **Phylogeny of jasmonate signaling components**. **a**, COI1 (orthogroup OG0000273). **b**, JAZ (orthogroup OG0000262). **c**, MYC (orthogroup OG0000558). d, NINJA (orthogroup OG0000816). **e**, TPL (orthogroup OG0000602). Green colour indicates the *Anthoceros* sequences.

B

JAZ

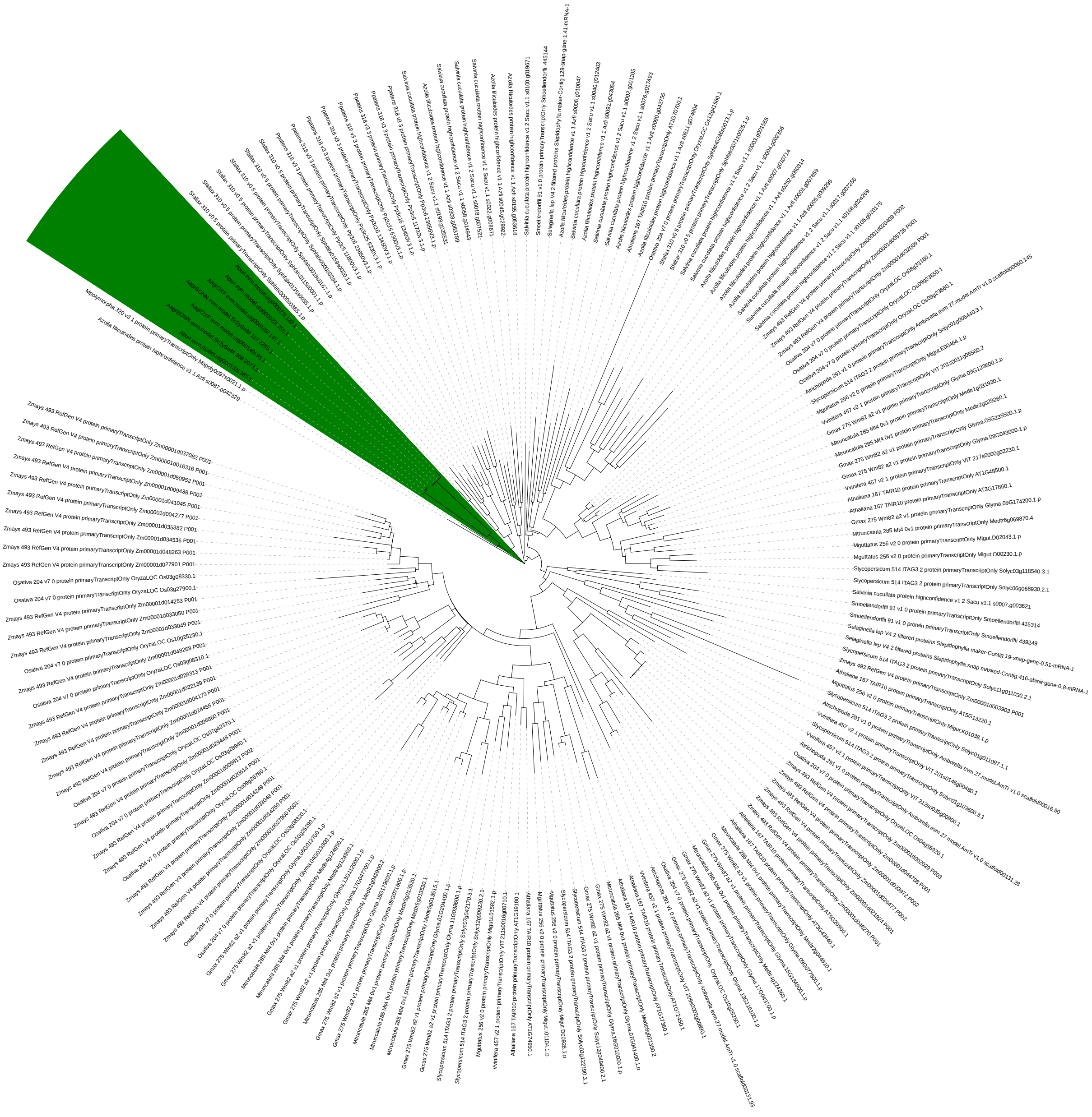

Supplementary Figure 10. **Phylogeny of jasmonate signaling components**. continued.

c

MYC

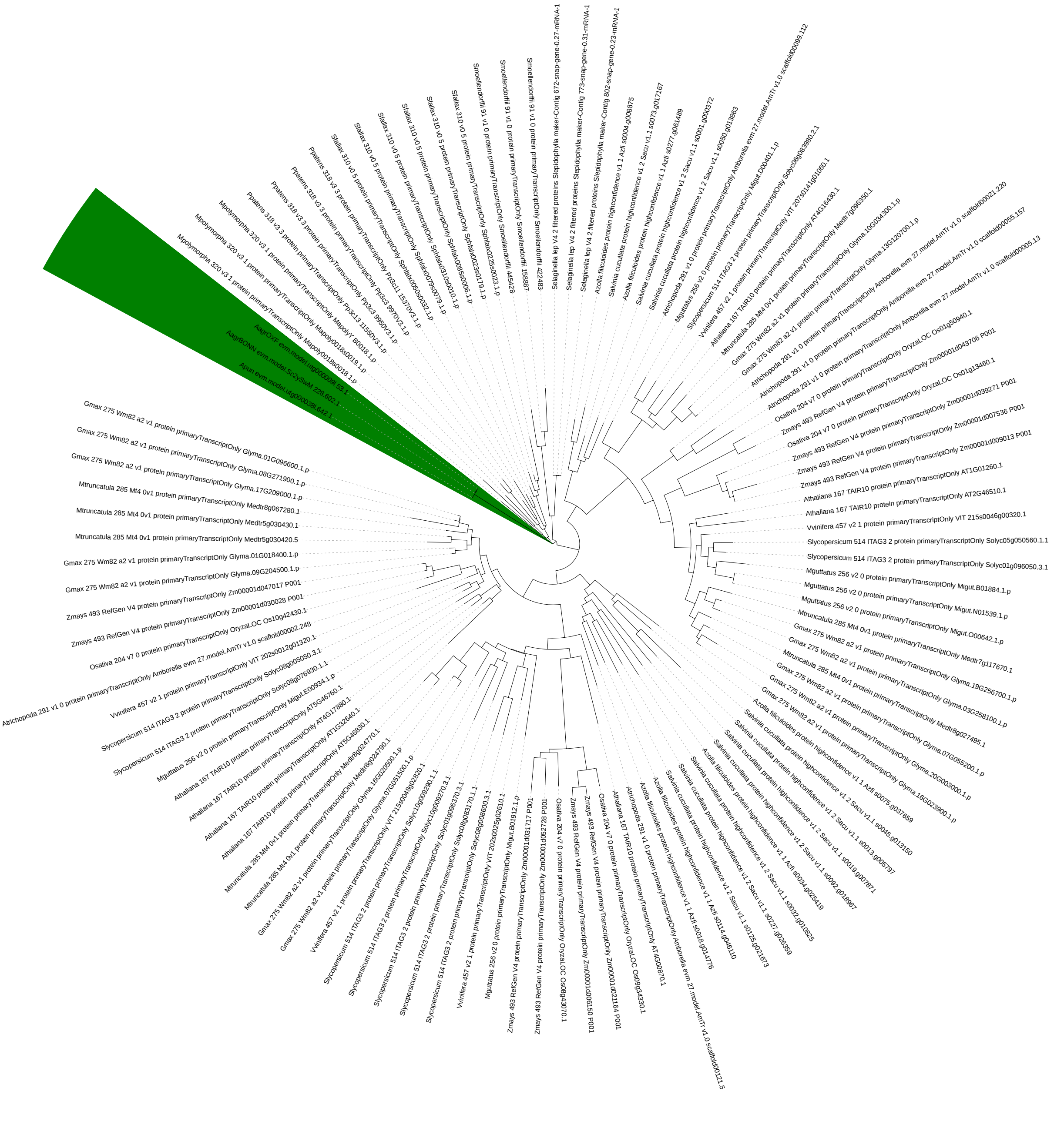

Supplementary Figure 10. **Phylogeny of jasmonate signaling components.** continued.

D

NINJA

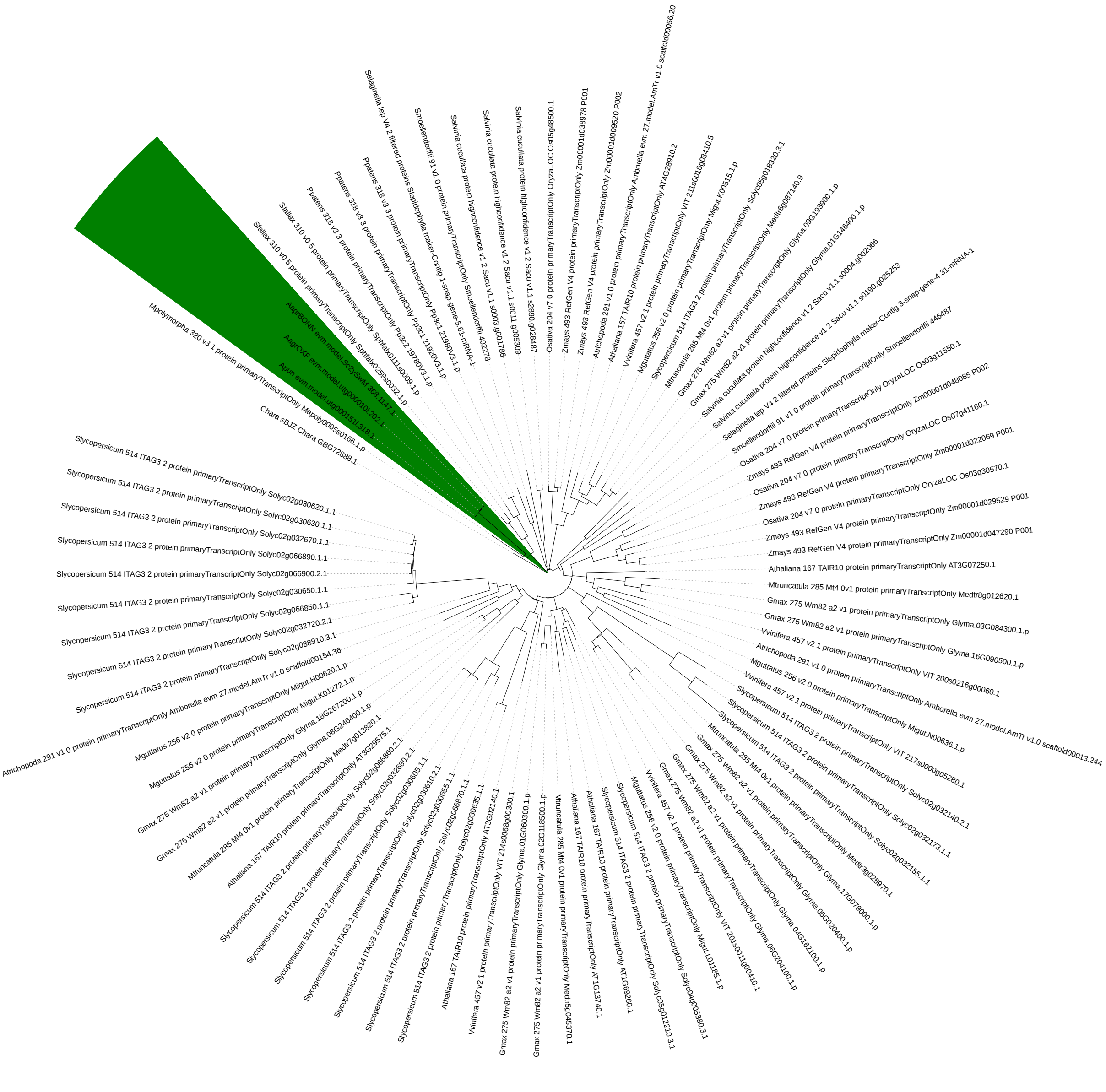

Supplementary Figure 10. **Phylogeny of jasmonate signaling components.** continued.

E

TPL

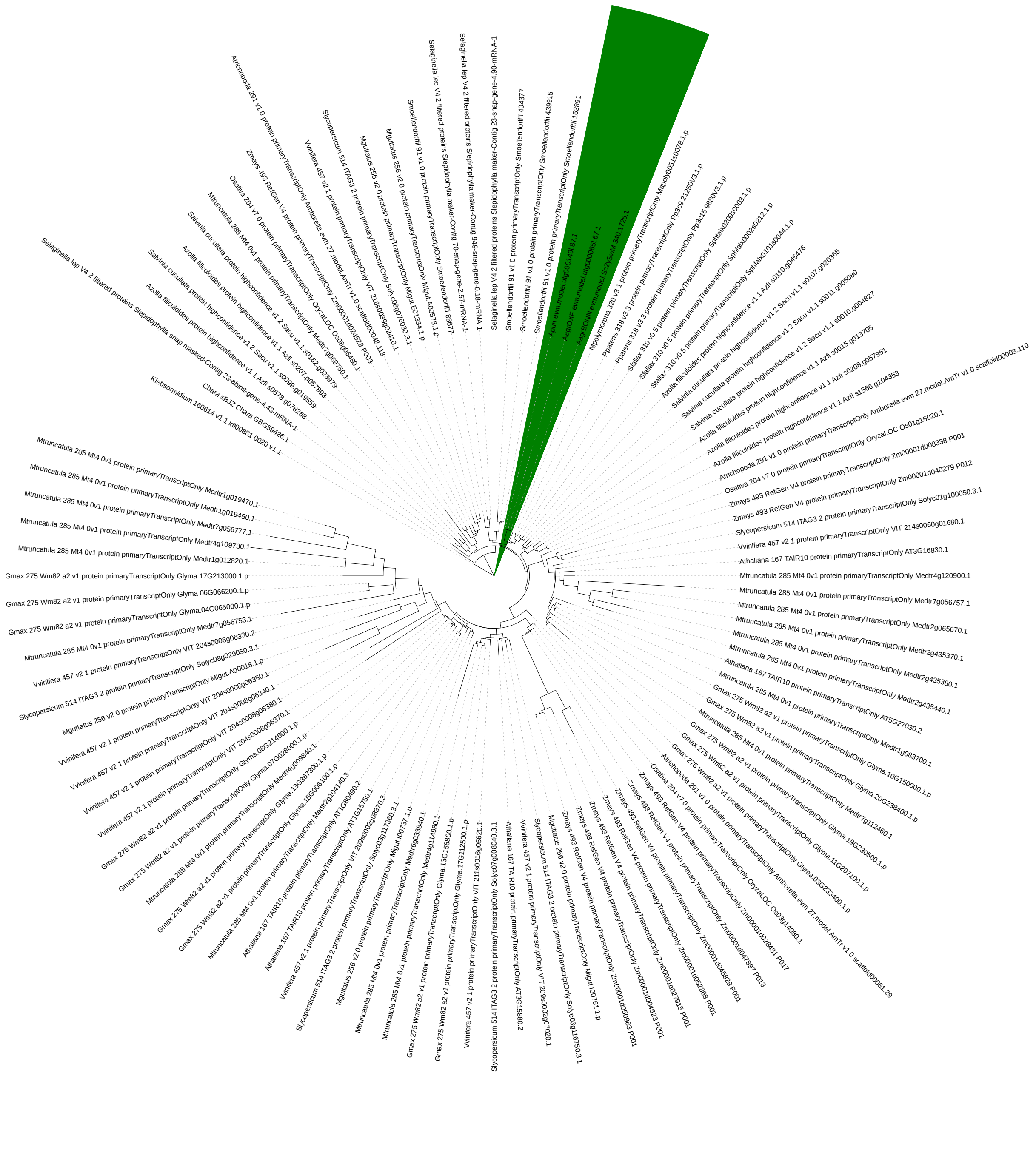

Supplementary Figure 10. **Phylogeny of jasmonate signaling components.** continued.

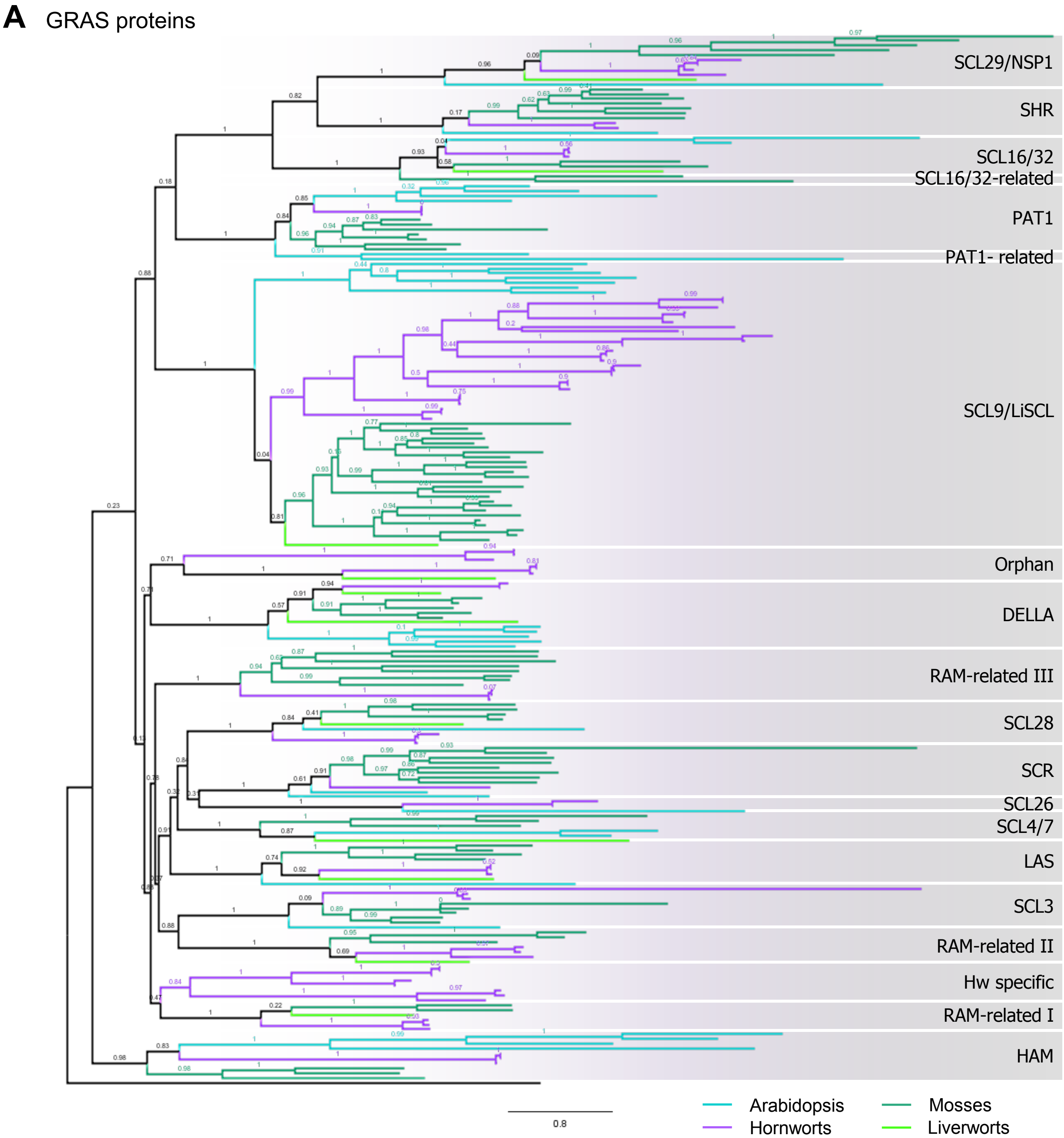

B

	M. polymorpha	S. fallax	P. patens	A. agrestis Bonn	A. agrestis Oxf	A. punctatus	A. thaliana
SCL9/LISCL	1	18	7	8	8	10	7
PAT1	0	5	2	1	1	1	4
SCL16/32	1	1	1	1	1	1	2
SCL29/NSP1	1	1	4	1	1	2	1
SHR	0	5	2	1	0	1	1
SCL3	0	2	3	1	1	1	1
DELLA	2	3	2	1	0	1	5
SCL28	1	2	2	1	1	1	1
SCR	0	4	3	0	0	1	2
SCL26	0	0	0	1	0	1	1
LAS	1	2	2	1	1	1	1
SCL4/7	1	1	1	0	0	0	2
HAM	0	2	1	1	1	1	4
Hw-specific	0	0	0	3	2	3	0
RAM-related I	1	0	2	1	1	1	0
RAM-related II	1	1	2	1	1	1	0
RAM-related III	0	4	4	1	1	1	0
Ungrouped	1	0	2	2	2	2	2

Supplementary Figure 11. **Gibberellin-related genes**. **a,** Phylogeny of the GRAS gene family with DELLA. **b,** Summary of GRAS gene family in the *Anthoceros* genomes. The *Anthoceros* genomes apparently lack GID1 (**c**) and GID2 (**d**).

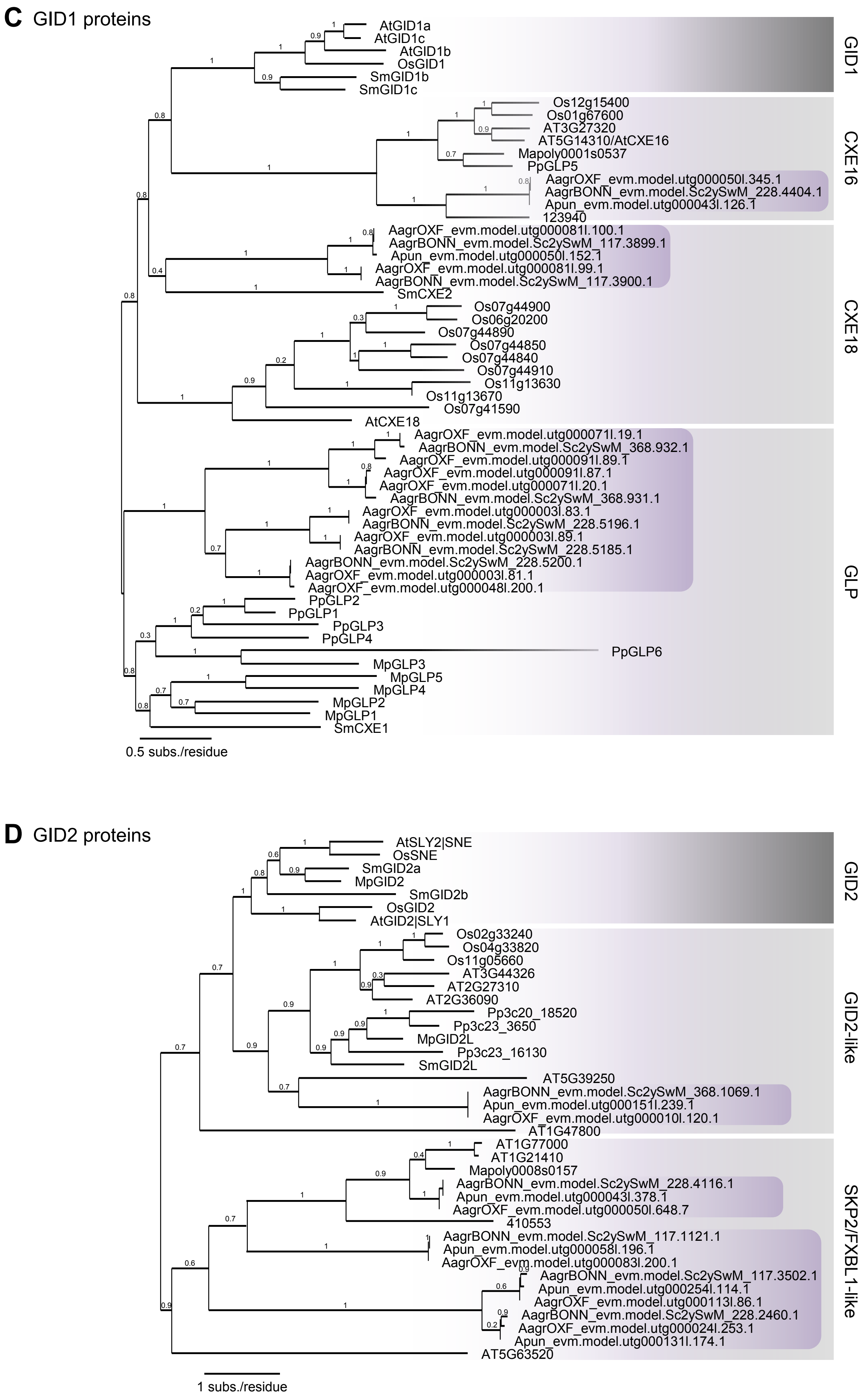

Supplementary Figure 11. **Gibberellin-related genes**. continued.

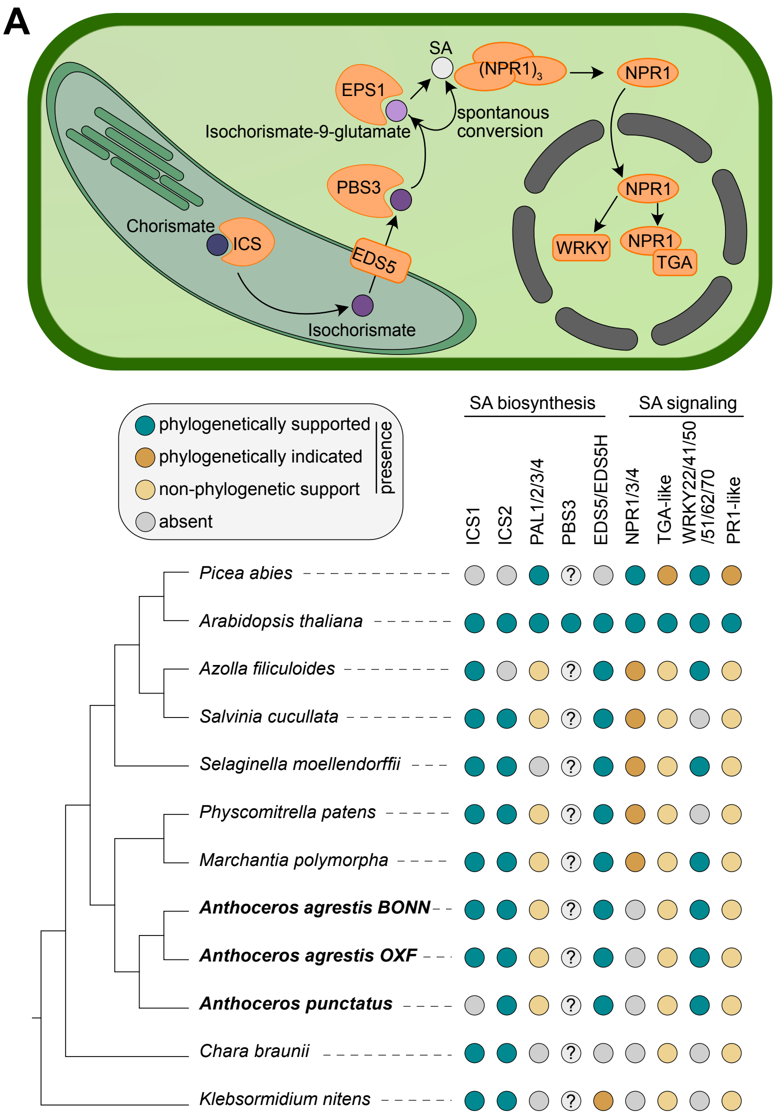

Supplementary Figure 12. **Salicylic acid (SA) biosynthesis and signaling in hornworts**. **a**, Identification of SA biosynthesis and signaling components in *Anthoceros* genomes. “Phylogenetically supported” means the hornwort homologs clustered with the respective *Arabidopsis* sequences with a bootstrap support ≥70, whereas “phylogenetic indicated” means with a bootstrap support ≥50. “Non-phylogenetic support” refers to having evidence from one of the additional analyses (e.g. presence of the required domain or high sequence similarity). Phylogeny of **b**, NPR, **c**, TGA, **d**, PR1, **e**, WRKY, **f**, ICS1/2, **h**, PAL, **g**, PBS3, and **i**, EDS5.

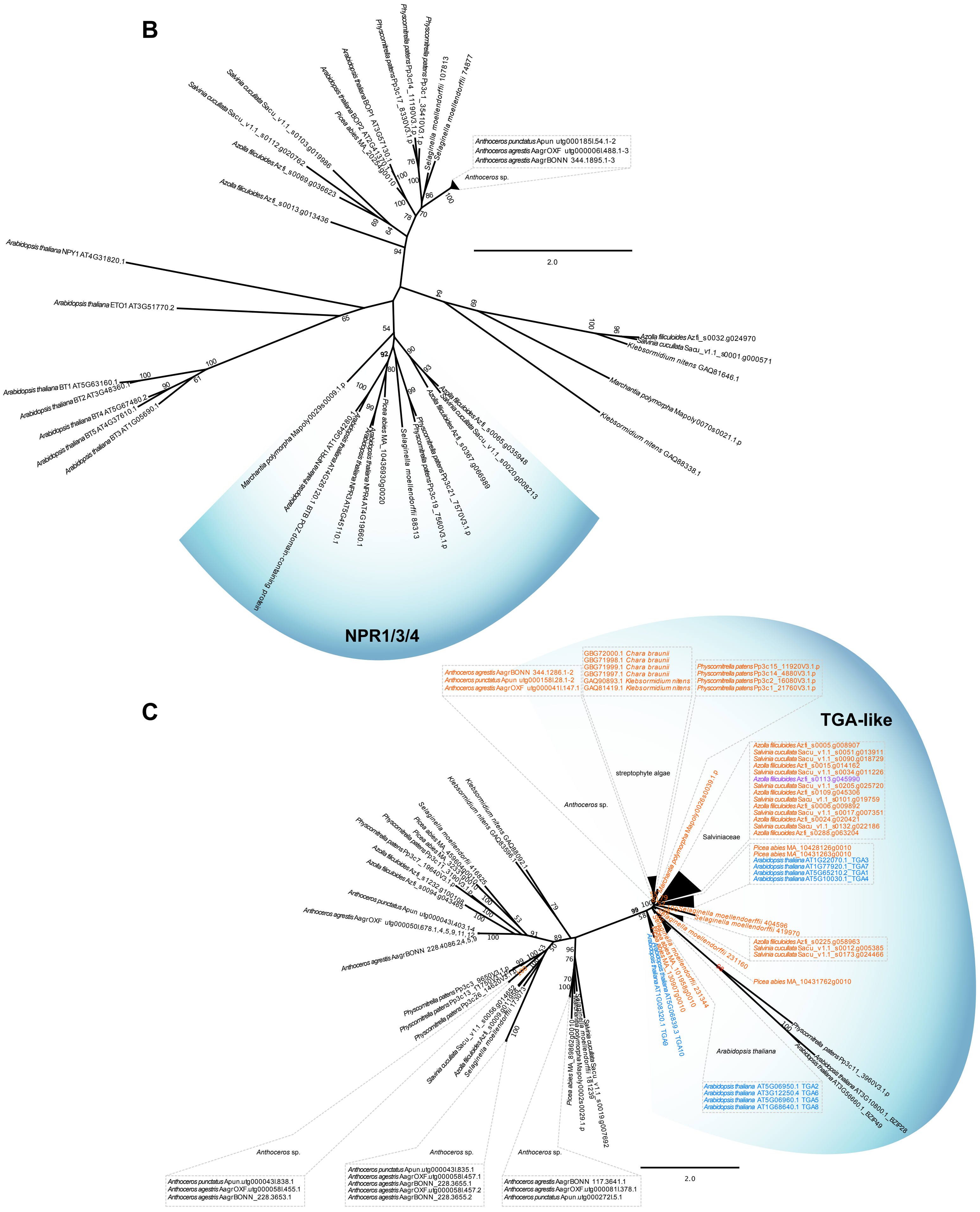

Supplementary Figure 12. **Salicylic acid (SA) biosynthesis and signaling in hornworts.** continued.

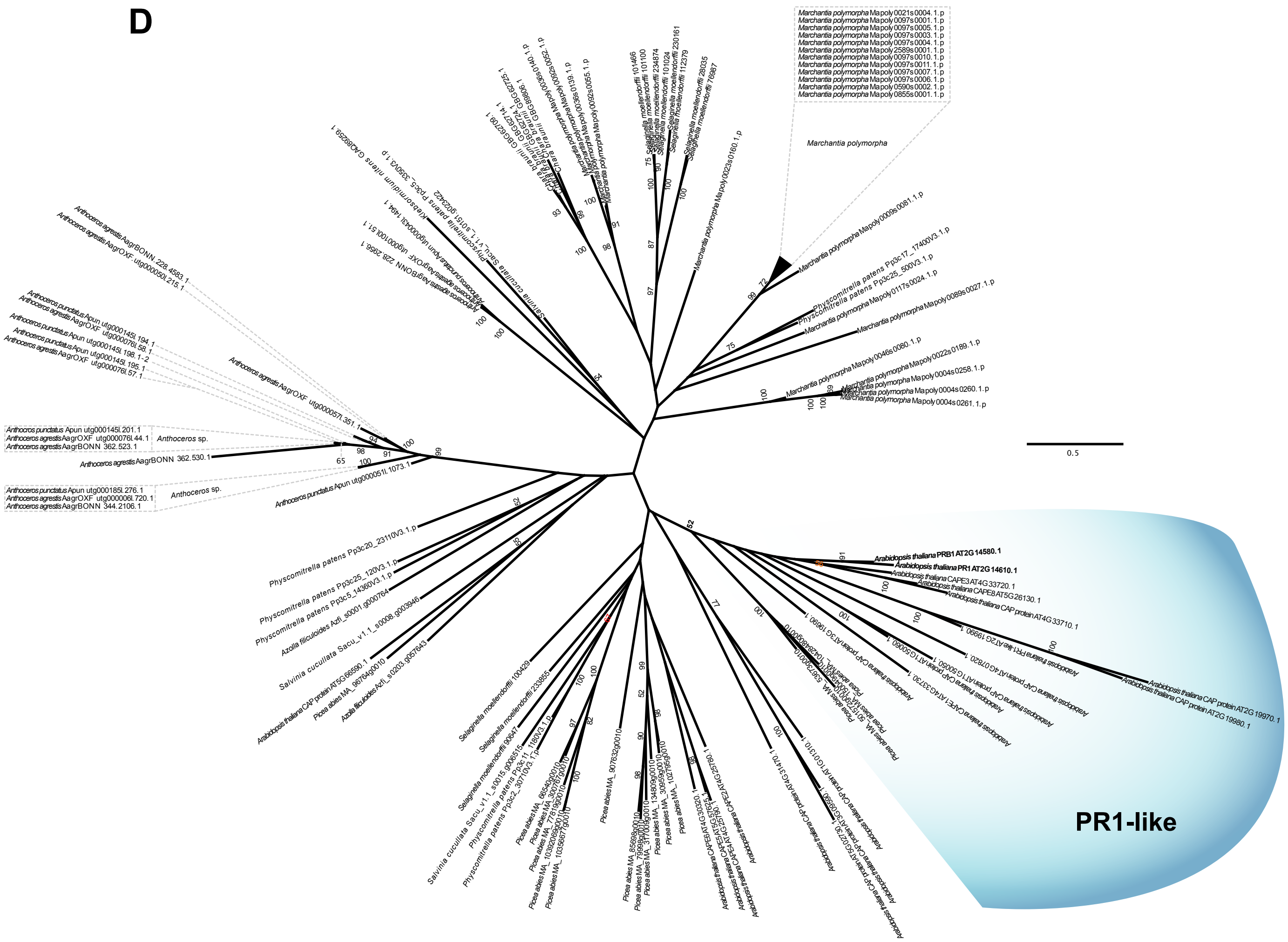

Supplementary Figure 12. **Salicylic acid (SA) biosynthesis and signaling in hornworts.** continued.

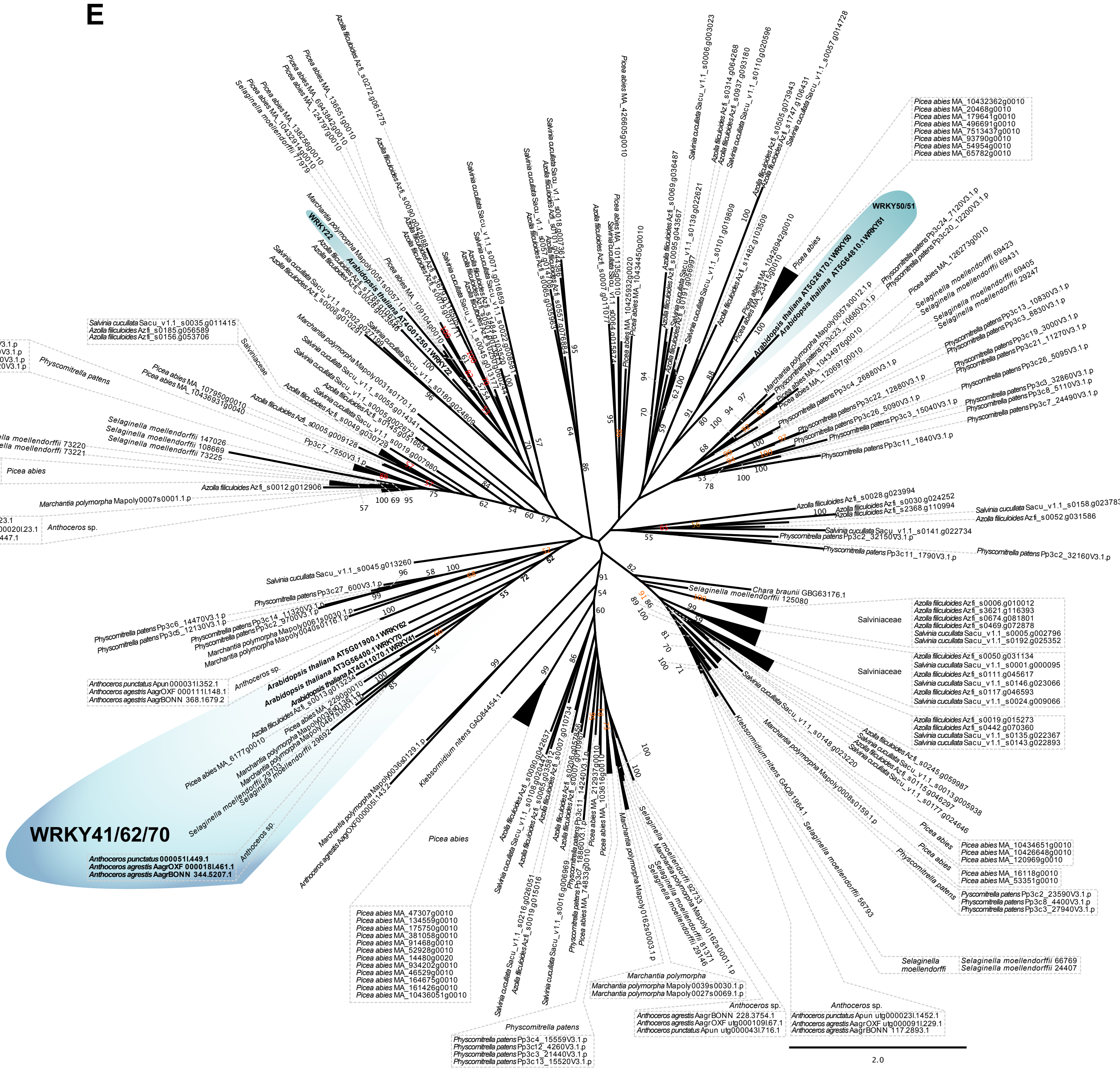

Supplementary Figure 12. **Salicylic acid (SA) biosynthesis and signaling in hornworts.** continued.

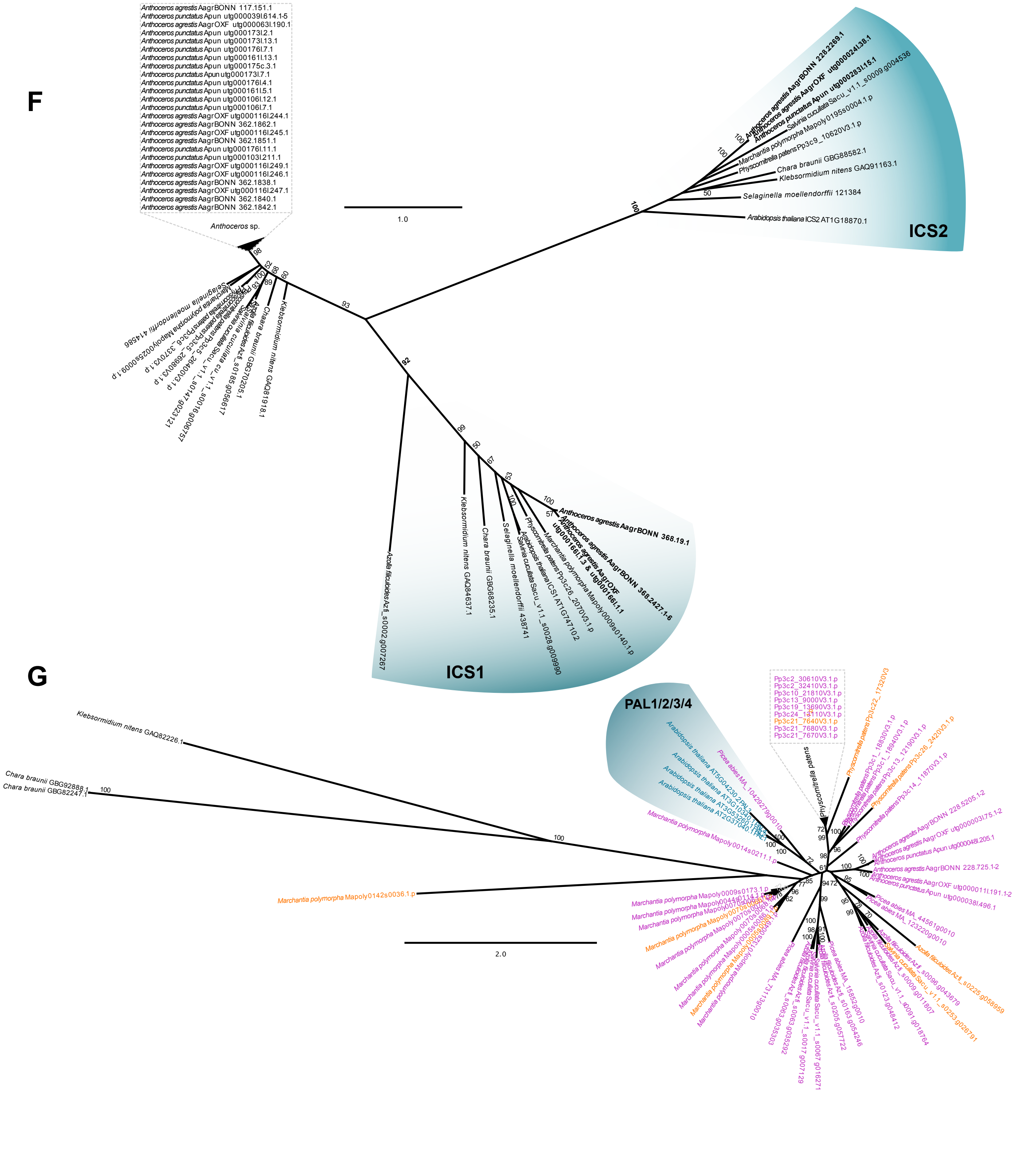

Supplementary Figure 12. **Salicylic acid (SA) biosynthesis and signaling in hornworts.** continued.

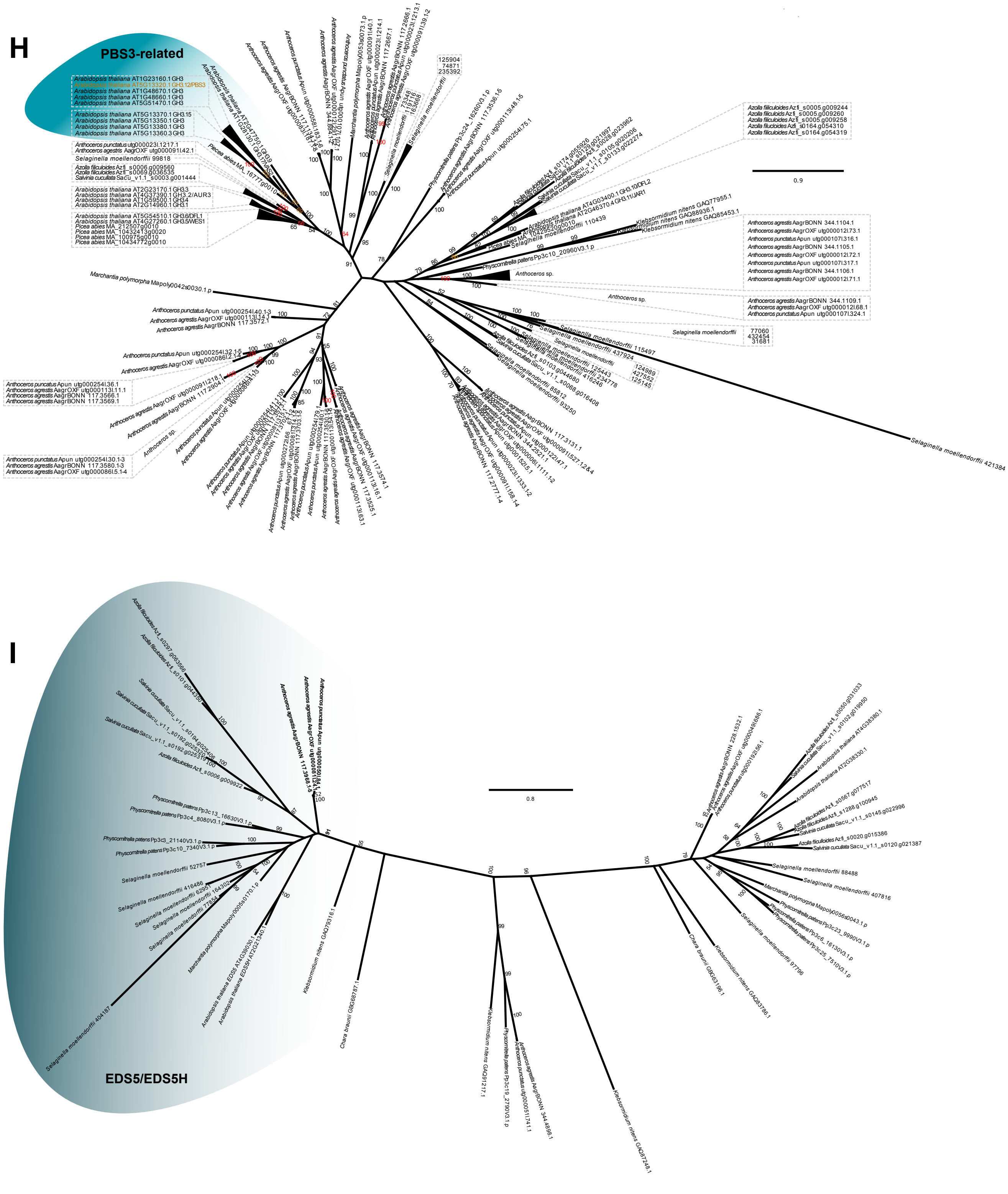

Supplementary Figure 12. **Salicylic acid (SA) biosynthesis and signaling in hornworts.** continued.

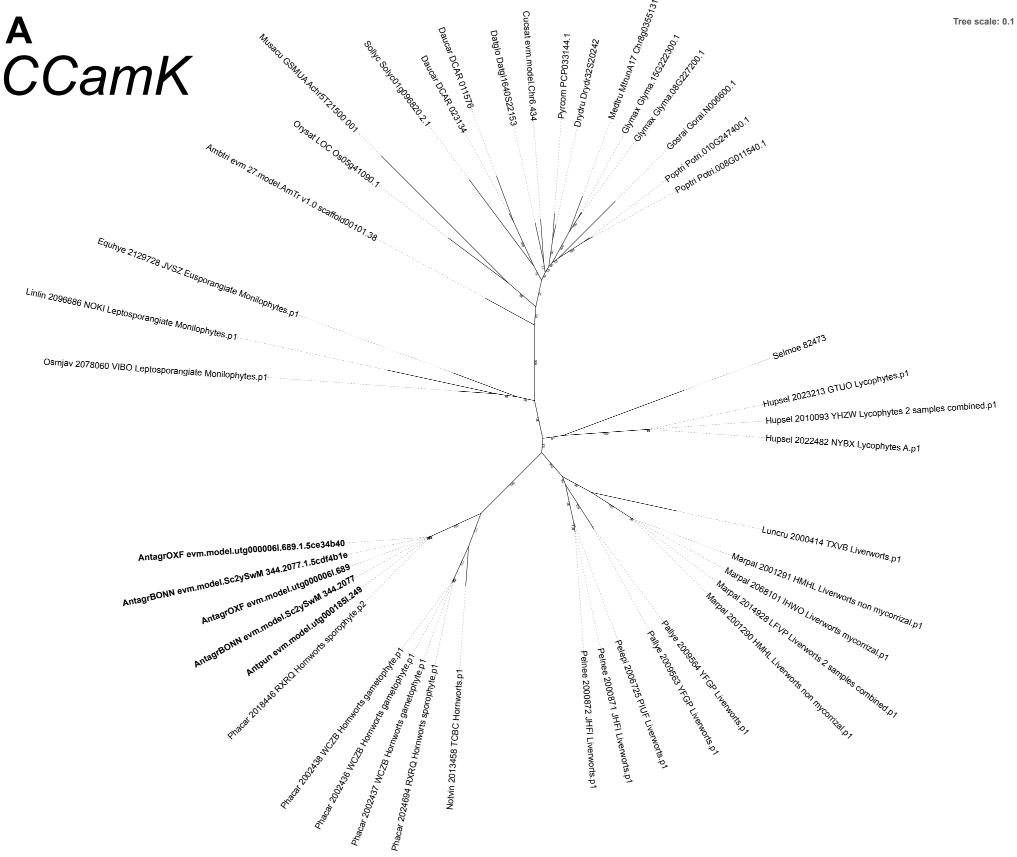

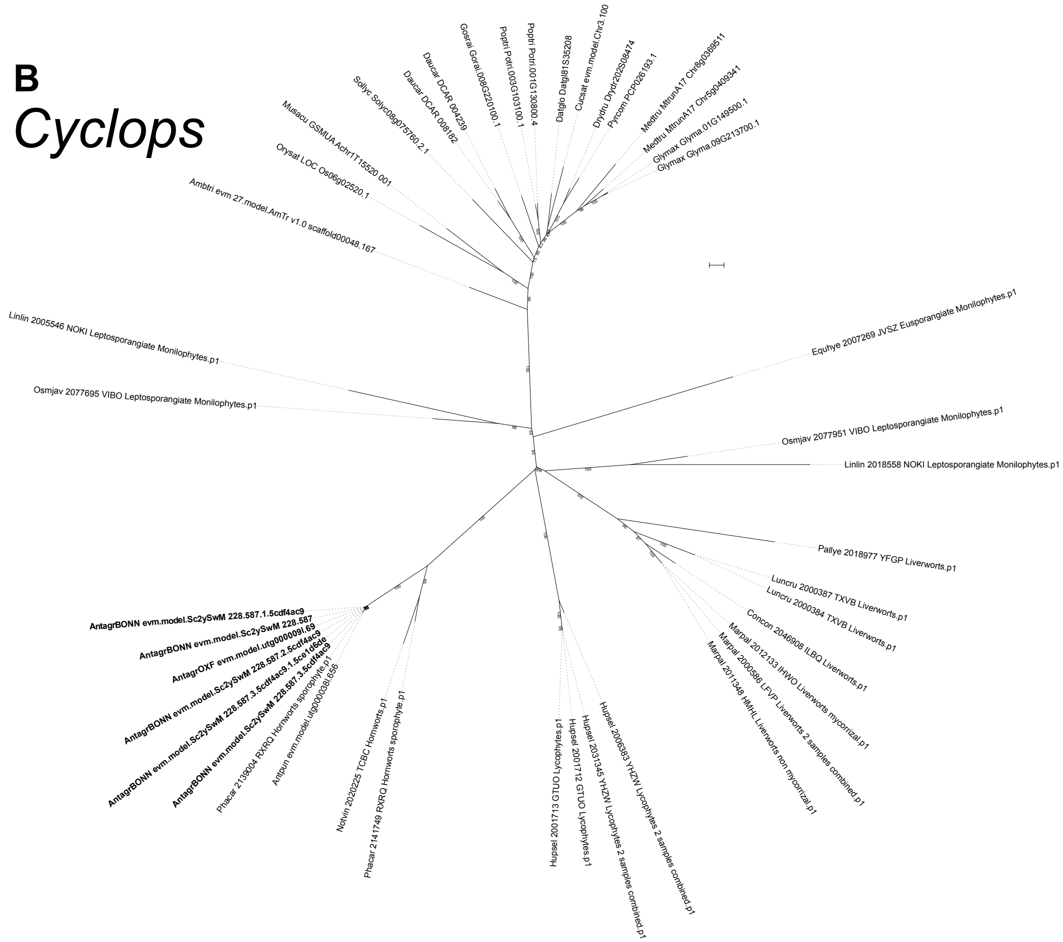

Supplementary Figure 13. **Phylogeny of arbuscular mycorrhizal symbiosis genes**. **a**, CCamK. **b**, Cyclops. **c**, Castor. **d**, SymRK. **e**, STR1/2. **f**, VAPYRIN. **g**, RAD1. **h**, RAM1.

C

Castor

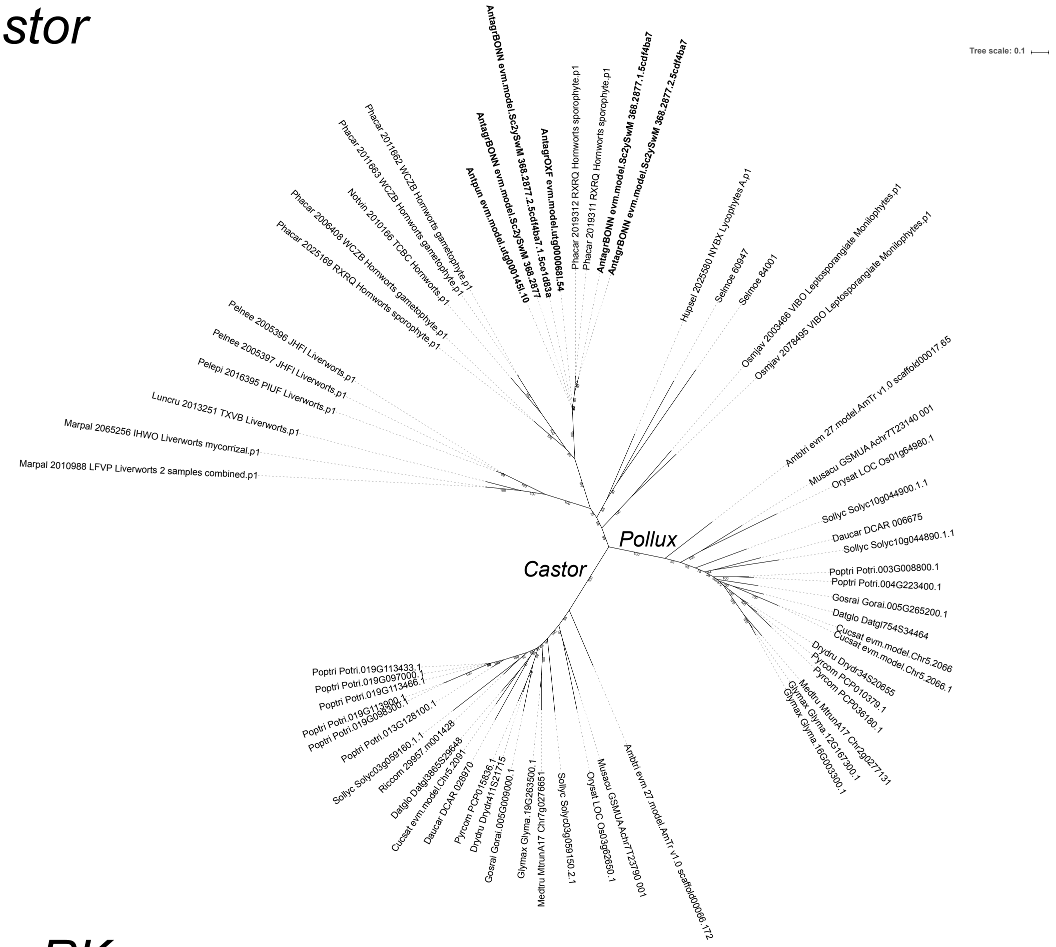

D

SymRK

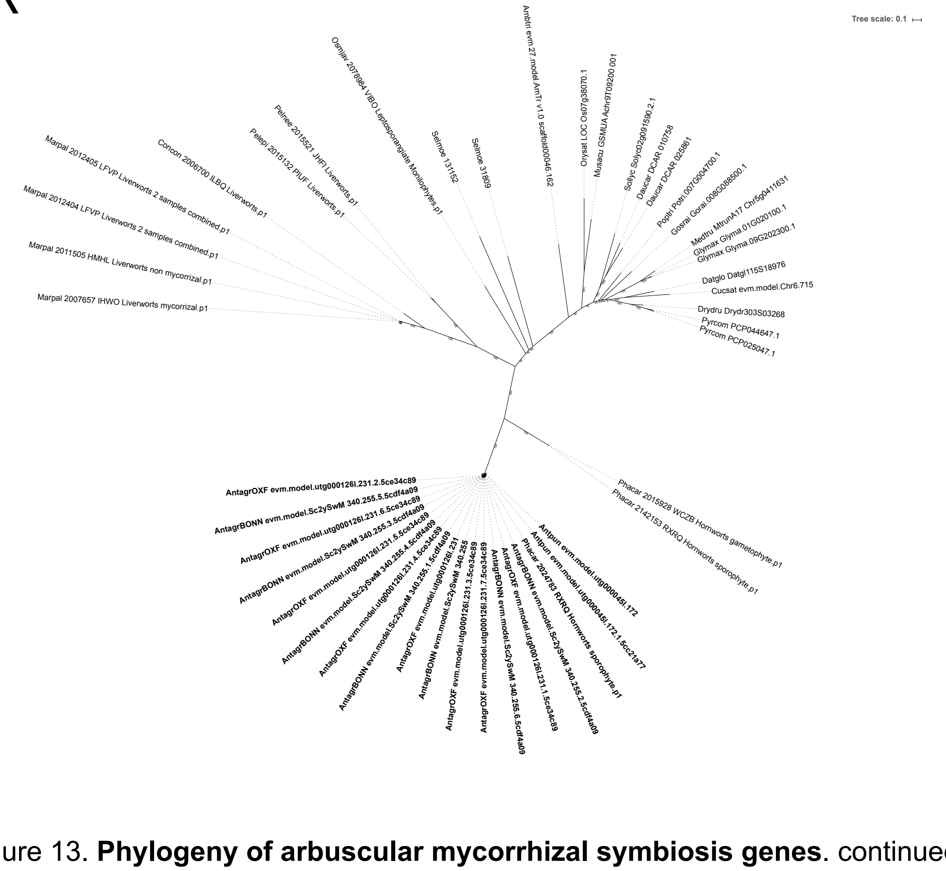

Supplementary Figure 13. **Phylogeny of arbuscular mycorrhizal symbiosis genes**. continued.

E

STR1/STR2

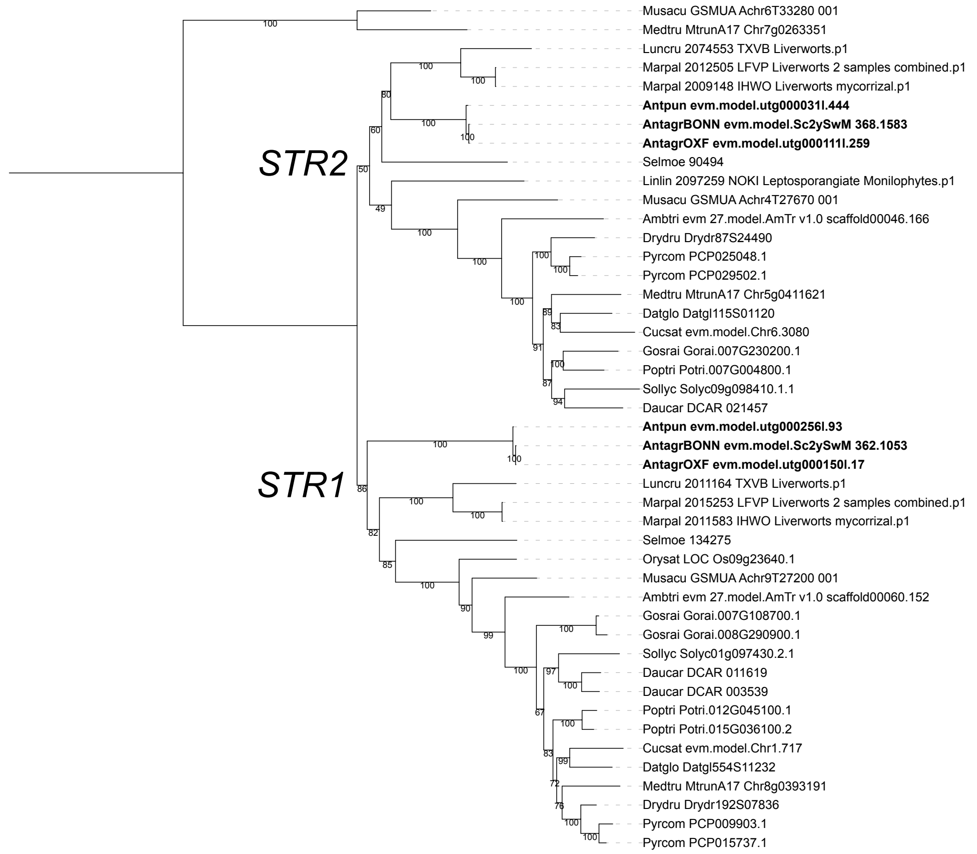

F

VAPYRIN

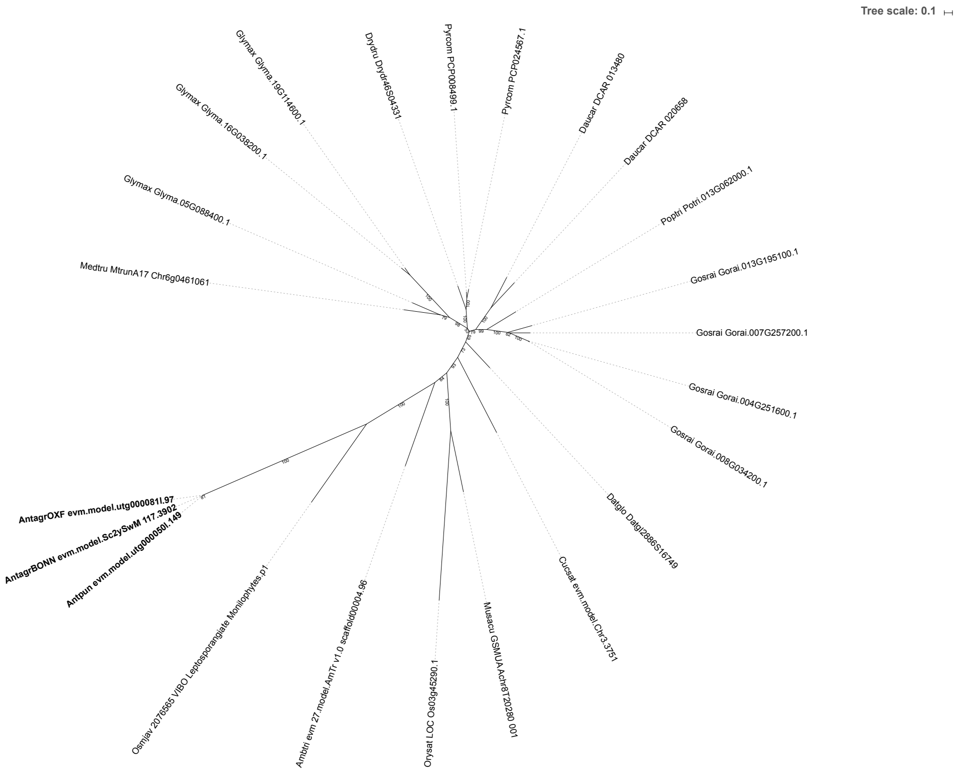

Supplementary Figure 13. **Phylogeny of arbuscular mycorrhizal symbiosis genes**. continued.

G

RAD1

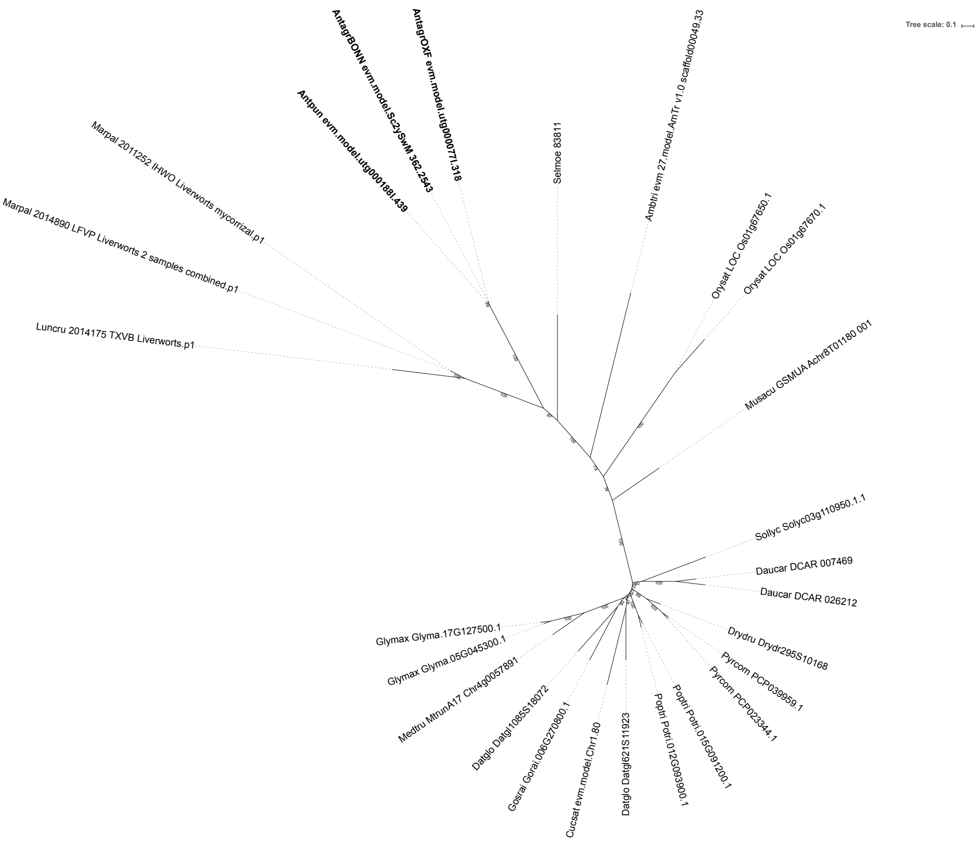

H

RAM1

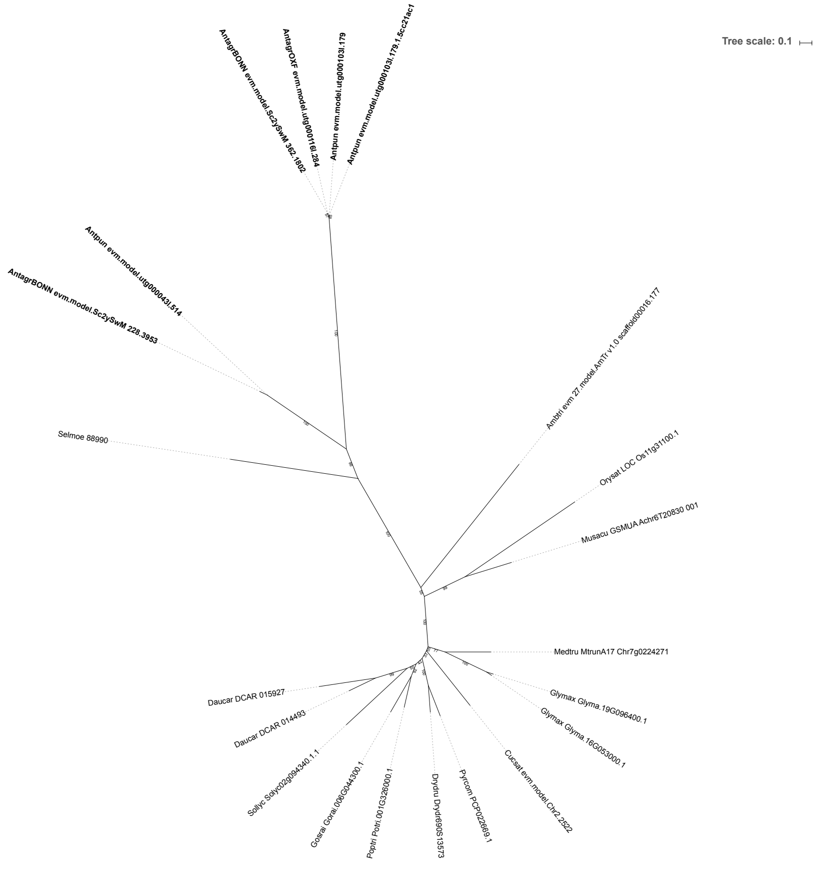

Supplementary Figure 13. **Phylogeny of arbuscular mycorrhizal symbiosis genes**. continued.

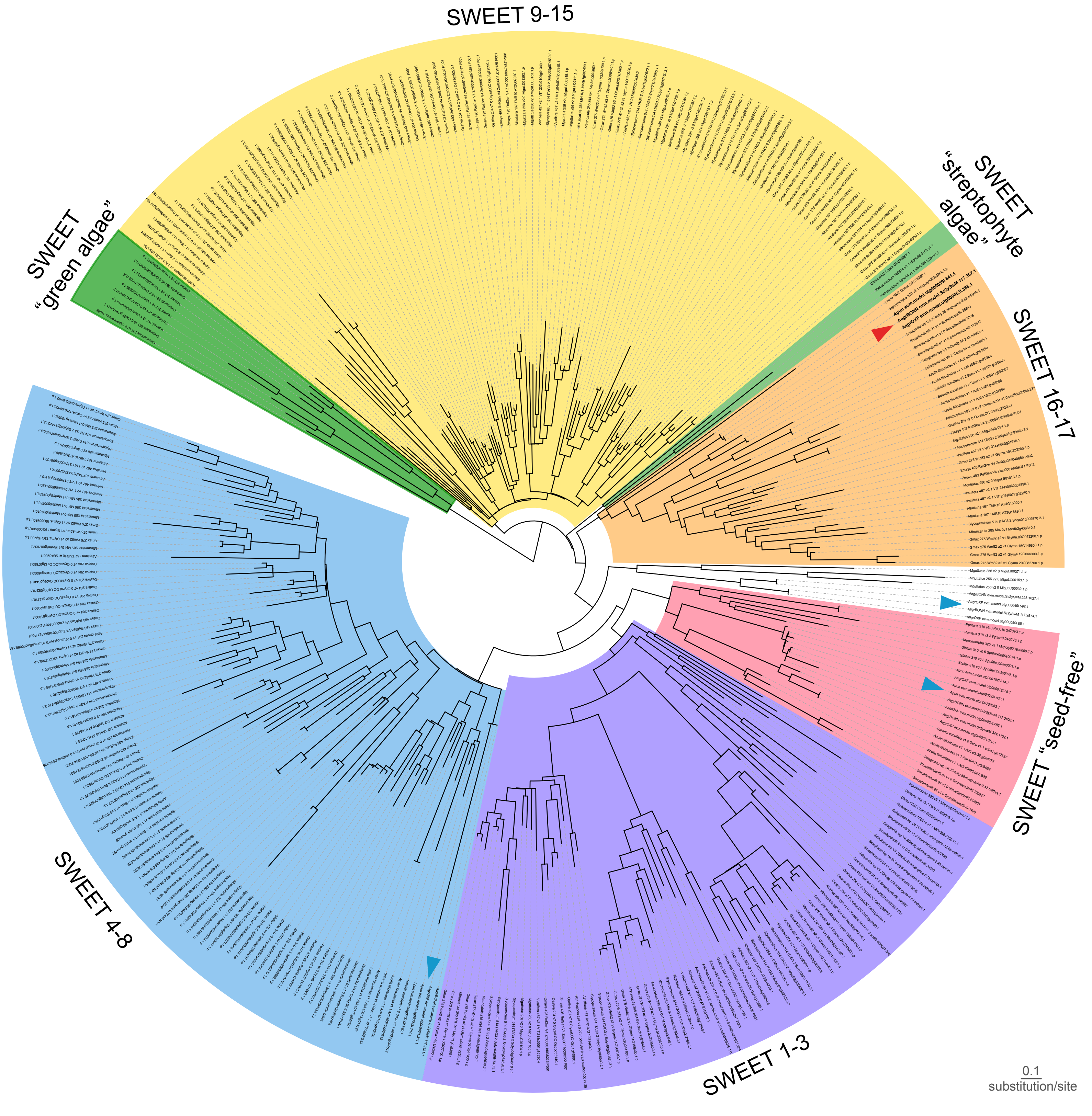

Supplementary Figure 14. **Phylogeny of SWEET**. Hornwort SWEET homologs are marked by arrowheads. Cyanobacteria symbiosis-induced SWEET homologs are located in the SWEET 16/17 clade (red arrowhead). The phylogeny is based on the orthogroup OG0000068.

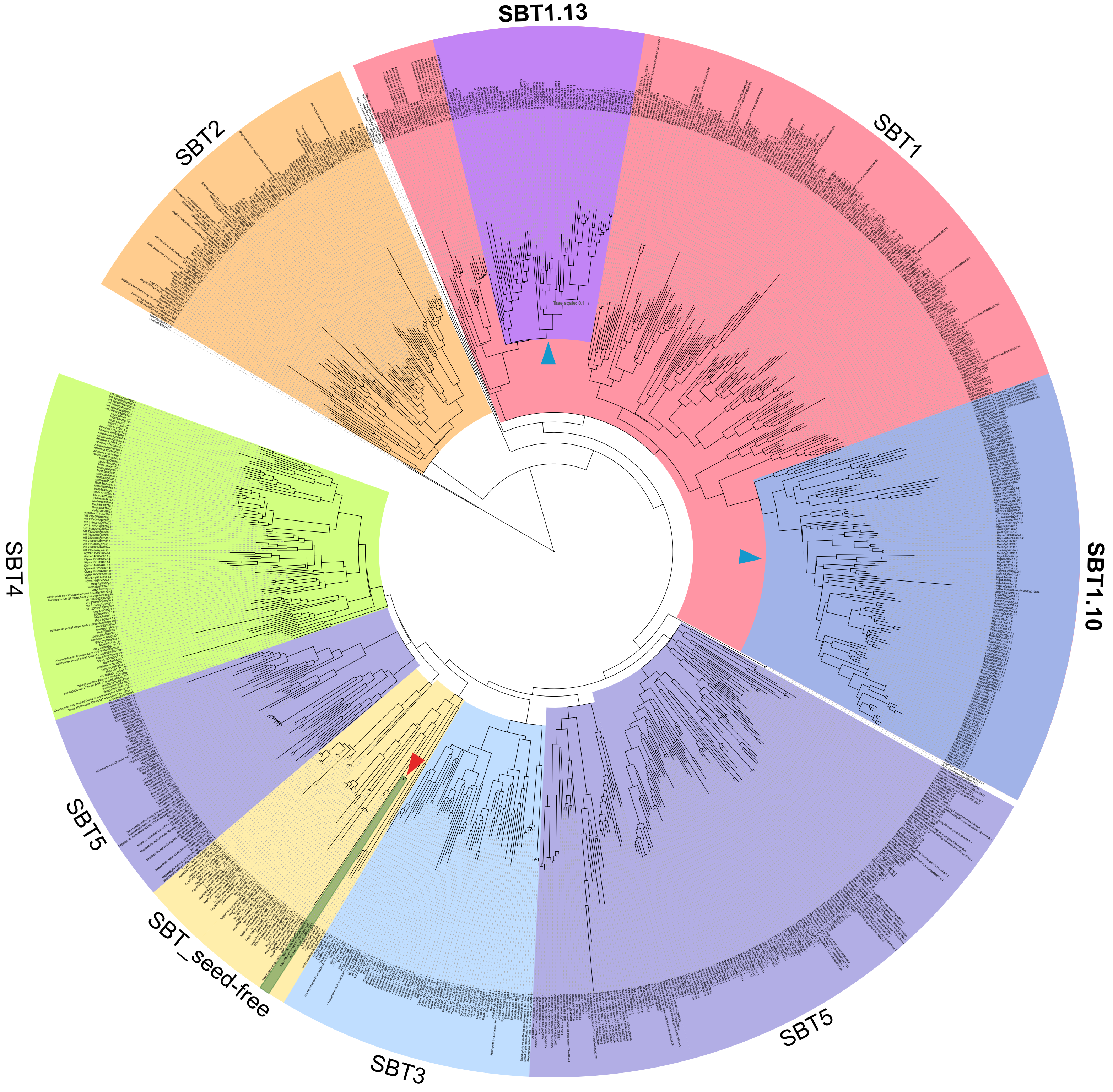

Supplementary Figure 15. **Phylogeny of subtilases (SBT)**. Cyanobacteria symbiosis-induced SBT homologs are marked by red arrowheads. SBT involved in rhizobial, mycorrhizal, and actinorhizal interactions are from the SBT1.10 and SBT1.13 clades (marked by blue arrowheads). The phylogeny is based on the orthogroup OG0000008 and clade designations followed Taylor and Qiu (2017; doi.org/10.1094/MPMI-10-16-0218-R).

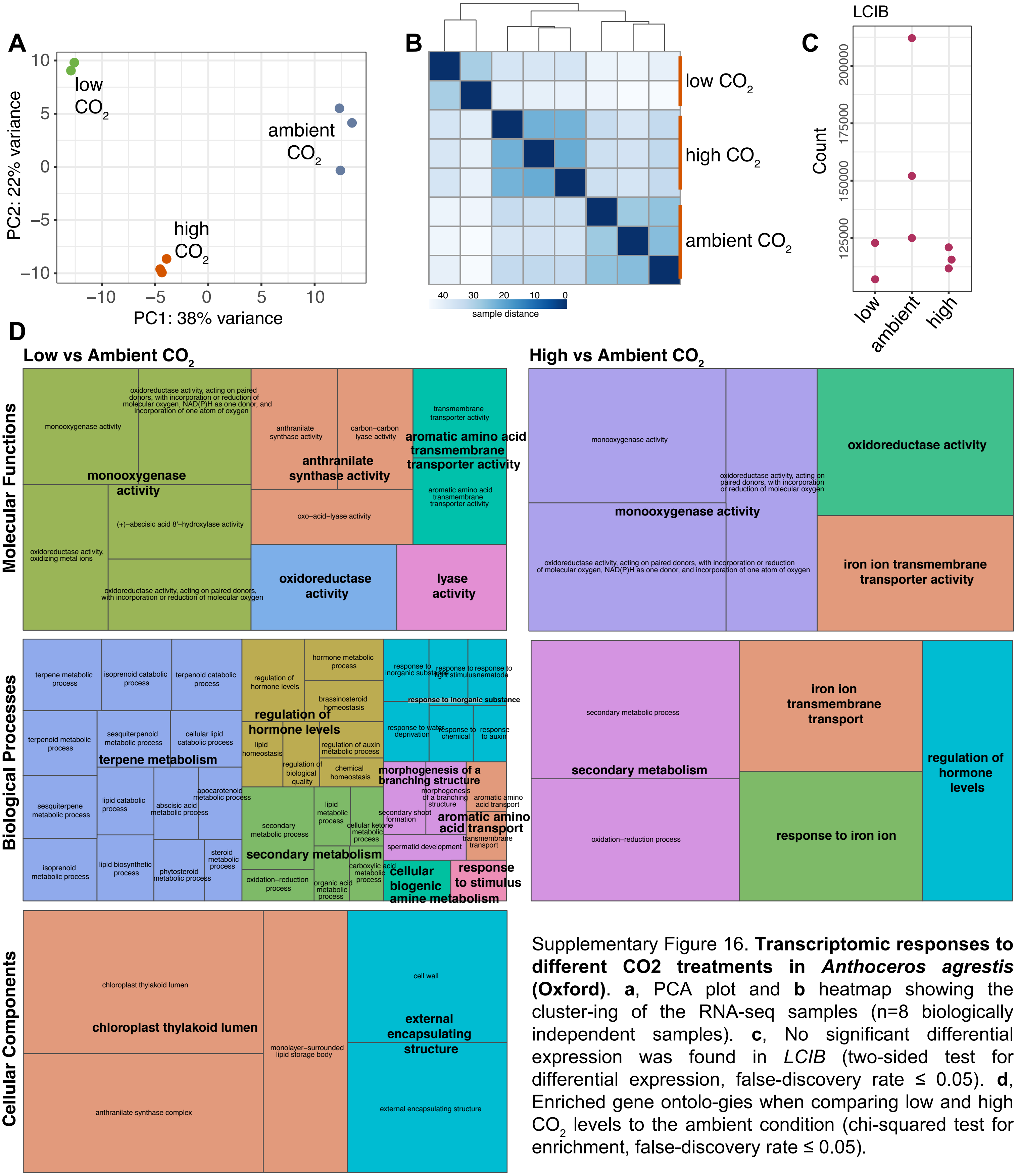

Supplementary Figure 16. **Transcriptomic responses to different CO2 treatments in *Anthoceros agrestis* (Oxford)**. **a**, PCA plot and **b** heatmap showing the cluster-ing of the RNA-seq samples (n=8 biologically independent samples). **c**, No significant differential expression was found in *LCIB* (two-sided test for differential expression, false-discovery rate ≤ 0.05). **d**, Enriched gene ontolo-gies when comparing low and high CO_2 levels to the ambient condition (chi-squared test for enrichment, false-discovery rate ≤ 0.05).

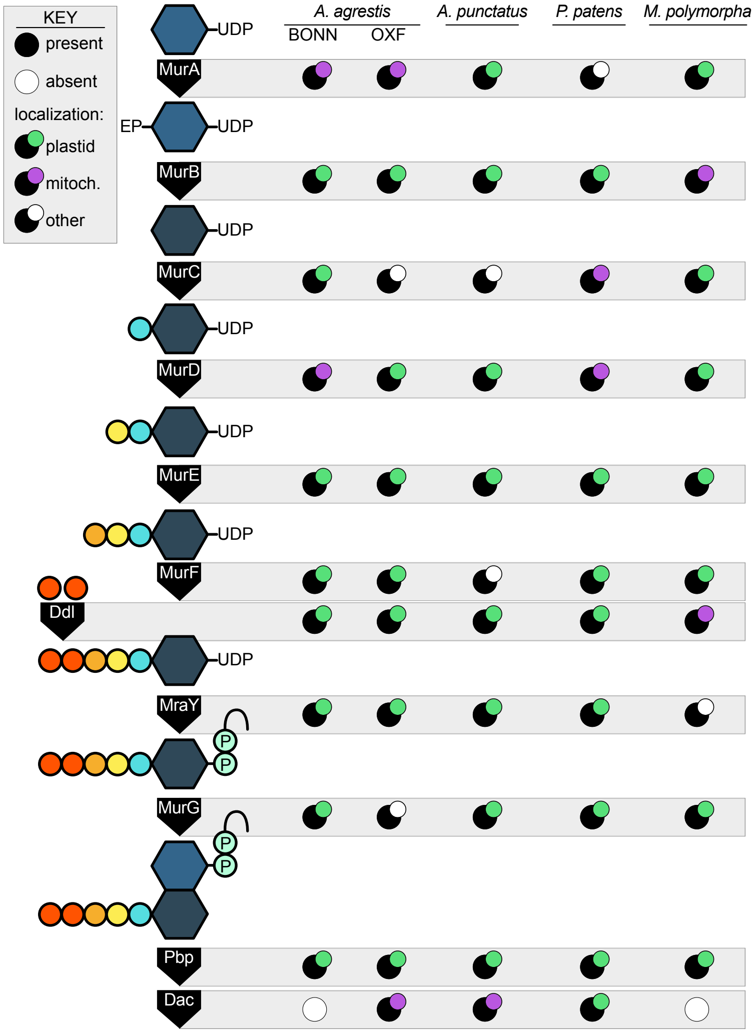

Supplementary Figure 17. **Genes involved in the peptidoglycan (PG) biosynthesis**. The *Anthoceros* genomes have the full homologous genetic chassis for PG biosynthesis, similar to *Physcomitrella patens* and *Marchantia polymorpha*.

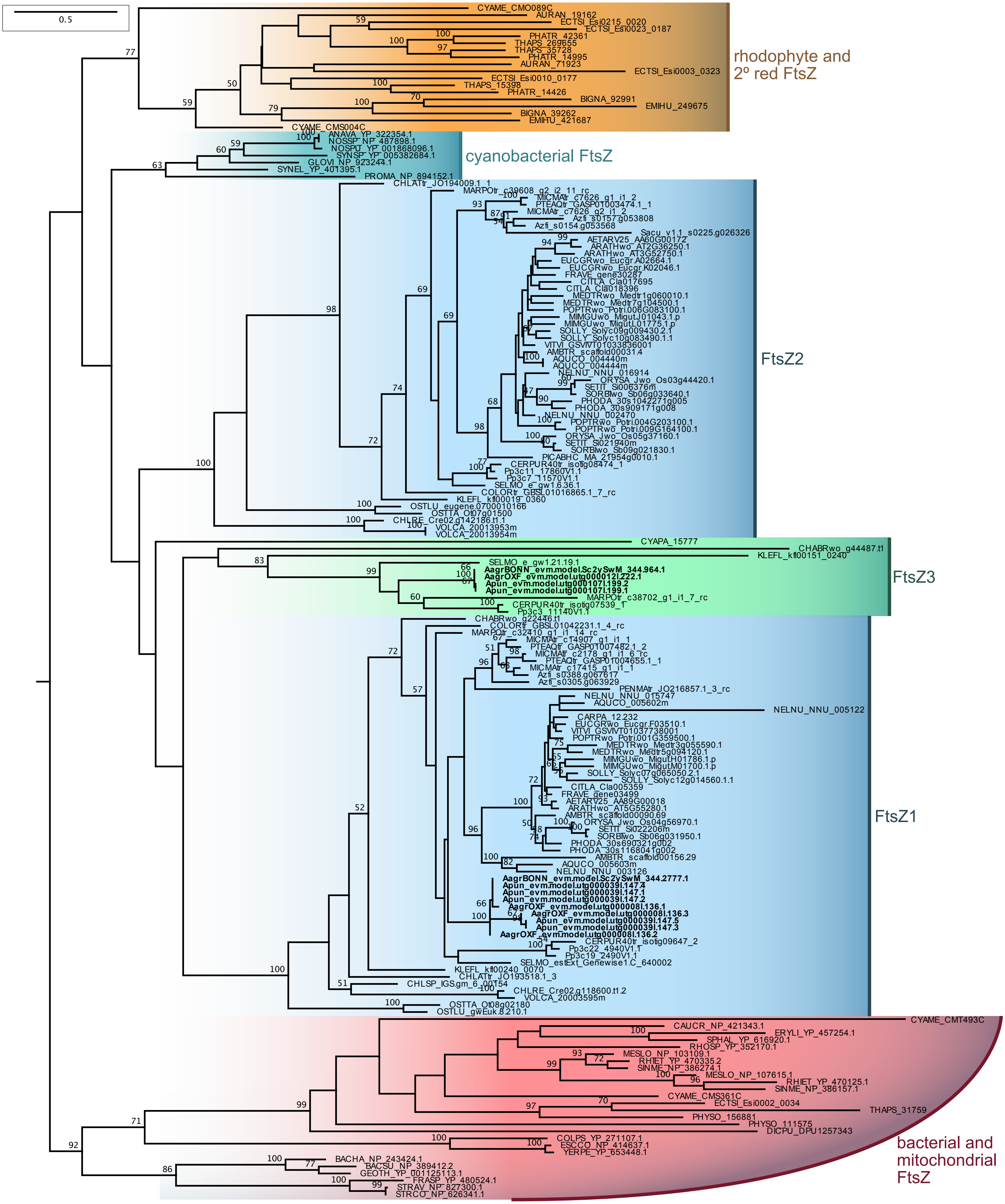

Supplementary Figure 18. **Phylogeny of FtsZ genes**. The *Anthoceros* genomes lack the FtsZ2 homolog.
