# Supplementary material for: Anthoceros genomes illuminate the origin of land plants and the unique biology of hornworts
Source: Nat Plants. 2020 Mar 13;6(3):259–72. doi: 10.1038/s41477-020-0618-2 (PMC8075897; doi:10.1038/s41477-020-0618-2)
Supplement: Supplementary file 2 — Reporting Summary [file 41477_2020_618_MOESM2_ESM.pdf]

nature research

Corresponding author(s): Peter Szovenyi

Last updated by author(s): 01/31/2020

Reporting Summary

Nature Research wishes to improve the reproducibility of the work that we publish. This form provides structure for consistency and transparency in reporting. For further information on Nature Research policies, see Authors & Referees and the Editorial Policy Checklist.

Statistics

For all statistical analyses, confirm that the following items are present in the figure legend, table legend, main text, or Methods section.

n/a	Confirmed	
☐	☒	The exact sample size (*n*) for each experimental group/condition, given as a discrete number and unit of measurement
☐	☒	A statement on whether measurements were taken from distinct samples or whether the same sample was measured repeatedly
☐	☒	The statistical test(s) used AND whether they are one- or two-sided<br>*Only common tests should be described solely by name; describe more complex techniques in the Methods section.*
☒	☐	A description of all covariates tested
☐	☒	A description of any assumptions or corrections, such as tests of normality and adjustment for multiple comparisons
☐	☒	A full description of the statistical parameters including central tendency (e.g. means) or other basic estimates (e.g. regression coefficient) AND variation (e.g. standard deviation) or associated estimates of uncertainty (e.g. confidence intervals)
☐	☒	For null hypothesis testing, the test statistic (e.g. *F*, *t*, *r*) with confidence intervals, effect sizes, degrees of freedom and *P* value noted<br>*Give P values as exact values whenever suitable.*
☒	☐	For Bayesian analysis, information on the choice of priors and Markov chain Monte Carlo settings
☒	☐	For hierarchical and complex designs, identification of the appropriate level for tests and full reporting of outcomes
☒	☐	Estimates of effect sizes (e.g. Cohen's *d*, Pearson's *r*), indicating how they were calculated

Our web collection on statistics for biologists contains articles on many of the points above.

Software and code

Policy information about availability of computer code

Data collection	No software was used. We constructed paired-end DNA sequencing libraries with approx. 400 bp insert sizes for standard WGS sequencing using Illumina HiSeq and Novaseq machines. We also prepared RNA-seq libraries and sequenced them on Hiseq and Novaseq machines. We generated long-reads using high-molecular weight DNA on the Oxford Nanopore Minion machine using R9 flow cells.
Data analysis	Trimmomatic v0.39, samtools v1.9, bwa v0.7.17, Albacore, v2.3.3, Jellyfish v1.1.12, Genomescope, MaSuRCAv3.2.8, pilon v1.22, HiRise assembler v2.1.2, minimap2 v2.14-r883, miniasm v0.3-r179, racon v1.3.1, blobtools v1.1.1, Salmon v0.13.1, Deseq2 v3.9, HiSat2 v2.1.0, stringtie v2.0.3, BINGO v3.0.3, REVIGO, RepeatModeler v1.0.11, LTR_retriever v2.0, RepeatMasker v4.0.8, portcullis v1.1.2, Trinity v2.8.4, PASA v2.3.3, Mikado v1.5, exonerate v2.2.0, BRAKER v2.1.2, augustus v3.3.2, EVidenceModeler v1.1.1, Mugsy v1.2.3, D-GENIES dot plot v1.2.0, MUMmer v3.0 and v4.0, nucmer v3.0, Assemblytics, OrthoFinder v2.3.3, i-AdHoRe v3.0, Tandem Repeats Finder v4.09, DupPipe, Genewise v2.4.1, PAML v1.3.1, MCscanX v1.0, MAFFT v7.427, Mesquite v3.6, MEGA X, RAxML v8.2.12, PHYLIP v3.697, MAFFT v7.450, BLAST v2.9.0+, TrimAl v1.4, IQ-TREE v1.6.1 and v1.6.1.2, ModelFinder v1.6.1, iTOL platform v4.4.2, MUSCLE v3.8.31, fastp v0.19.10, qualimap v2.2.1, blat v35, picard v2.21.4, BUSCO v3, TranslatorX, ASTRAL-III v5.6.3, DiscoVista v1.0, TAPscan, InterPro 77.0, Pfam 32.0, CDD v3.17, HMMER v3.3

For manuscripts utilizing custom algorithms or software that are central to the research but not yet described in published literature, software must be made available to editors/reviewers. We strongly encourage code deposition in a community repository (e.g. GitHub). See the Nature Research guidelines for submitting code & software for further information.

Data

Policy information about availability of data

All manuscripts must include a data availability statement. This statement should provide the following information, where applicable:

- Accession codes, unique identifiers, or web links for publicly available datasets
- A list of figures that have associated raw data
- A description of any restrictions on data availability

All the raw sequences are deposited to NCBI SRA under the BioProject PRJNA574424, PRJNA574453, and to ENA under the study accessions PRJEB34763,

PRJEB34743 (Supplementary Tables 2-3) and will become public upon publication.

The genome assemblies, annotations ("Submitted.zip") as well as alignment matrices and tree files ("phylogeny_dataset.zip") can be found on Figshare (private link: https://figshare.com/s/e3ebfc9104663c5d08de). A future genome browser will be available for the public upon publication.

Field-specific reporting

Please select the one below that is the best fit for your research. If you are not sure, read the appropriate sections before making your selection.

☐ Life sciences ☐ Behavioural & social sciences ☒ Ecological, evolutionary & environmental sciences

For a reference copy of the document with all sections, see nature.com/documents/nr-reporting-summary-flat.pdf

Ecological, evolutionary & environmental sciences study design

All studies must disclose on these points even when the disclosure is negative.

Study description

Here we provide three high-quality genome assemblies and their annotations for the genus Anthoceros. We use these data to refine our inferences on the nature of land plant MRCA and to gain new insights into hornwort biology.

Research sample

Cultures of Anthoceros agrestis (Oxford and Bonn strains) and A. punctatus were all derived from a single spore, (haploid gametophyte tissue) and axenically propagated and maintained on either BCD or Hatcher's medium. Supplementary Table 13 shows the origin and specimen voucher for each of the three strains. We have been developing the three Anthoceros isolates as model systems for multiple years. Our selection was tailored by the potential of these strains to become model species for hornworts.

Sampling strategy

No statistical test was used to determine sample size. In gene expression studies three or two biological replicates were used to estimate differential gene expression (significance and fold change). The number of biological replicates used was tailored by the difficulty in obtaining tissue samples and extracting high-quality RNA from Anthoceros tissues.

Data collection

DNA was derived from axenic isolates of the three Anthoceros accessions. For gene expression studies RNA was extracted from tissues of the very same isolates after vegetative propagation. Data was recorded and analyzed as described in the Authors Contribution section of the main text.

Timing and spatial scale

Samples for DNA-sequencing were collected when available. Samples for RNA-seq experiments followed well-defined developmental stages described in the manuscript.

For the CO2 response experiment, we subjected the plant cultures to one of the three CO2 environments at 150 (low), 400 (ambient), and 800 (high) ppm in a CO2-controlled growth chamber for 10 days (12/12hr day/night cycle). These CO2 concentrations match up with those used in previous experiments investigating hornwort pyrenoid function. Therefore, our results are directly comparable with observations of previous investigations. Sampling intervals also followed previous experiments to ensure comparability.

For the cyanobacterial symbiosis experiment, plants were transferred from solid BCD plates to flasks with 100 ml BCD media solution, and placed on an orbital shaker with 130 rpm for two weeks. For the cyano-/N+ and cyano-/N- conditions, plants were transferred to fresh new BCD solution with and without KNO3, respectively and grown for 10 days before harvest. These conditions and time intervals correspond to those that were previously applied in studies investigating hornwort-cyanobacteria symbiosis.

Data exclusions

Raw sequence data was quality filtered and trimmed using either fastp or trimmomatic (default parameters). Genome assemblies were filtered for contaminant scaffolds with blobtools and were excluded. Our data exclusion strategy was not pre-established. We used well accepted thresholds to filter out low-quality sequence data.

Reproducibility

RNA-seq experiments were carried out using two or three biological replicates. Genome assemblies were done with and without using long-reads and their collinearity compared. Bootstrap analyses were estimated for all nodes in gene trees.

Randomization

Bootstrap support for nodes in gene trees were estimated in a standard fashion through random resampling of columns in sequence alignments.

Blinding

No blinding was done for any of our analyses.

Did the study involve field work? ☐ Yes ☒ No

Reporting for specific materials, systems and methods

We require information from authors about some types of materials, experimental systems and methods used in many studies. Here, indicate whether each material, system or method listed is relevant to your study. If you are not sure if a list item applies to your research, read the appropriate section before selecting a response.

Materials & experimental systems

n/a	Involved in the study
☒	☐ Antibodies
☒	☐ Eukaryotic cell lines
☒	☐ Palaeontology
☒	☐ Animals and other organisms
☒	☐ Human research participants
☒	☐ Clinical data

Methods

n/a	Involved in the study
☒	☐ ChIP-seq
☒	☐ Flow cytometry
☒	☐ MRI-based neuroimaging
